# Spatial segregation of the biological soil crust microbiome around its foundational cyanobacterium, *Microcoleus vaginatus*, and the formation of a nitrogen-fixing cyanosphere

**DOI:** 10.1186/s40168-019-0661-2

**Published:** 2019-04-03

**Authors:** Estelle Couradeau, Ana Giraldo-Silva, Francesca De Martini, Ferran Garcia-Pichel

**Affiliations:** 1 School of Life Sciences, Arizona State University, 427 E. Tyler Mall, Tempe, AZ 85287 USA; 2 Laboratoire Biogéosciences, Université de Bourgogne, Dijon, France; 3 Present Address: Joint Genome Institute (DOE), Lawrence Berkeley National Lab (LBNL), 2800 Mitchell Drive, Walnut Creek, CA 94598 USA; 4 Center for Fundamental and Applied Microbiomics, Biodesign Institute, Arizona State University, 1001 S. McAllister Ave., Tempe, AZ 85282 USA

**Keywords:** Biocrust, Cyanosphere, *Microcoleus vaginatus*, Diazotrophs

## Abstract

**Background:**

Biological soil crusts (biocrusts) are a key component of arid land ecosystems, where they render critical services such as soil surface stabilization and nutrient fertilization. The bundle-forming, filamentous, non-nitrogen-fixing cyanobacterium *Microcoleus vaginatus* is a pioneer primary producer, often the dominant member of the biocrust microbiome, and the main source of leaked organic carbon. We hypothesized that, by analogy to the rhizosphere of plant roots, *M. vaginatus* may shape the microbial populations of heterotrophs around it, forming a specialized cyanosphere.

**Results:**

By physically isolating bundles of *M. vaginatus* from biocrusts, we were able to study the composition of the microbial populations attached to it, in comparison to the bulk soil crust microbiome by means of high-throughput 16S rRNA sequencing. We did this in two *M. vaginatus*-dominated biocrust from distinct desert biomes. We found that a small, selected subset of OTUs was significantly enriched in close proximity to *M. vaginatus*. Furthermore, we also found that a majority of bacteria (corresponding to some two thirds of the reads) were significantly more abundant away from this cyanobacterium. Phylogenetic placements suggest that all typical members of the cyanosphere were copiotrophs and that many were diazotrophs (Additional file 1: Tables S2 and S3). Nitrogen fixation genes were in fact orders of magnitude more abundant in this cyanosphere than in the bulk biocrust soil as assessed by qPCR. By contrary, competition for light, CO_2,_ and low organic carbon concentrations defined at least a part of the OTUs segregating from the cyanobacterium.

**Conclusions:**

We showed that *M*. *vaginatus* acts as a significant spatial organizer of the biocrust microbiome. On the one hand, it possesses a compositionally differentiated cyanosphere that concentrates the nitrogen-fixing function. We propose that a mutualism based on C for N exchange between *M*. *vaginatus* and copiotrophic diazotrophs helps sustains this cyanosphere and that this consortium constitutes the true pioneer community enabling the colonization of nitrogen-poor soils. On the other hand, a large number of biocrust community members segregate away from the vicinity of *M*. *vaginatus*, potentially through competition for light or CO_2_, or because of a preference for oligotrophy.

**Electronic supplementary material:**

The online version of this article (10.1186/s40168-019-0661-2) contains supplementary material, which is available to authorized users.

## Background

Biological soil crusts (biocrusts) are soil-surface microbial communities based on microbial or cryptogamic phototrophs that develop in areas where light can penetrate directly to the soil surface unimpeded by a layer of plant litter ([1] for a primer and [2, 3] for monographs). They are prominent in arid lands, where they contribute several important ecosystem properties, including the protection of soils against erosion and nutrient fertilization of the areas they cover.

Most studies on the biology and ecology of biocrust organisms have centered on the primary producers (largely cyanobacteria, but also sometimes microalgae, lichens, and mosses), and much has been learned about their particular adaptations and ecology. And yet, biocrusts represent miniature ecosystems that are phylogenetically diverse, in which a variety of ecological functions are expressed. They constitute a particular type of soil microbiome, one in which the primary producers are an essential but certainly far from exclusive part [4, 5]. Pioneering filamentous, bundle-forming cyanobacteria, such as *Microcoleus vaginatus* and *M*. *steenstrupii*, initiate biocrust formation by stabilizing the surface of loose soils [6], allowing a succession that involves other cyanobacteria [7], bacteria [8], archaea [9], and fungi [10], as well as the lichens [11] and mosses [12] that are typical of the best developed crusts of milder environments. Most of the bacteria and archaea appear to be heterotrophs [9, 13], although crusts do contain significant populations of bacterial and archaeal chemolithotrophs that are crucial for nitrogen cycling [14, 15]. Under unusually long periods of wetness, spore-forming bacteria [16] or even methanogenic archaea [17] may develop sizeable biocrust populations. Microbial diversity and population density increase as succession proceeds [18]. Even in successionally young biocrusts, biomass (estimated as total cell counts, or DNA content) is orders of magnitude larger than those typical of desert soils, and the microbial communities within them show evidence of vertical stratification similar to those of microbial mats or biofilms [1]. At a larger, landscape scale, varying soil properties influence the biocrust microbiome composition [19], as do climatic variations at a continental scale [14, 20].

Biocrust microbes remain desiccated, and hence inactive, most of the time but, upon wetting, become quickly hydrated and active [21]. During pulses of activity, high metabolic rates constrained within small spaces result in the rapid formation of steep chemical gradients and microenvironments, which include oxygen-supersaturated zones close to the surface and anoxic zones some 1–3 mm deep [22]. Biocrusts are not only locally, but also globally relevant. They cover some 12% of the Earth’s continental area [23] and are major players in the global N cycle, as some ~ 31% of the biological nitrogen fixation on land can be attributed to their activity [24, 25]. Their global standing stocks have been estimated to reach in the order of 54 × 10^12^ g C [26]. The oldest fossil remains of biocrust communities date back to the Proterozoic [27], and it is thought that these systems were determinant for the global ecology of early continents before the advent of land plants [28].

In a large proportion of biocrusts worldwide, *M*. *vaginatus* plays a central role by being both a foundational species and a metabolic pivot to the biocrust community. Uniquely, *M*. *vaginatus* does not only fix carbon but also excrete a large fraction of its photosynthate directly into the soil [29, 30]. In using a plant analogy, *M*. *vaginatus* would serve both as a leaf and a root. However, *M*. *vaginatus* does not have the capacity to fix nitrogen [31, 32], so it remains somewhat surprising that a non-diazotroph be the main colonizer of such typically N-limited, bare arid soils. In mature crusts, most of the nitrogen fixation is attributed to heterocystous cyanobacteria [7] and, in early crusts that lack the latter, to the activity of heterotrophic diazotrophs [33].

We hypothesized that *M*. *vaginatus* may rely on the N_2_ fixation of other bacteria for their nitrogen needs and that such metabolic interaction may result in an enrichment of certain bacterial types in the proximity of its bundles within the biocrusts. By analogy to a plant rhizosphere [34], this sphere of influence would be the basis of a spatial “cyanosphere” (contraction of the words “cyanobacterium” and “sphere”) based on functional interactivity. We tested this hypothesis directly taking advantage of the large size of *M*. *vaginatus* bundles, which makes it possible to physically excise and isolate them from the rest of the biocrust community, enabling the characterization and comparative analyses of the microbial communities found close and away from its bundles.

## Results

### A cyanosphere composed of a selected subset of the biocrust microbiome exists around *M*. *vaginatus*

**Fig. 1:**
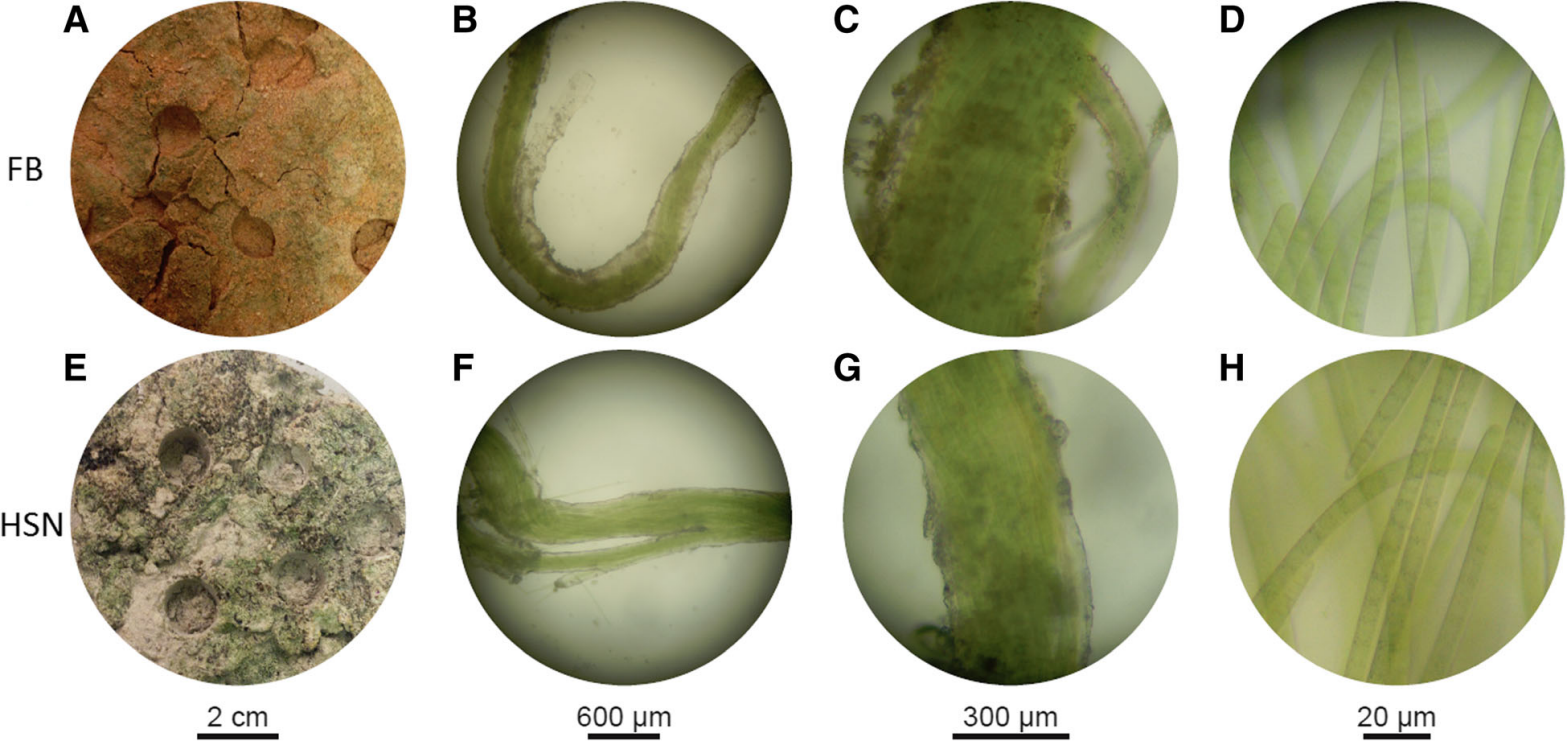
Biocrust samples from the Chihuahuan and the Great Basin deserts. **a**, **e** Top views of Chihuahuan (**a**) and Great Basin (**e**) biocrusts before bundle picking. Depressions are from coring for the bulk soil samples. **b**, **f** Examples of cyanobacterium bundles picked from the biocrust. Each bundle comprised the cyanobacterium and the exopolysaccharide sheath that bundles the filaments together and hosts the cyanosphere community. **c**, **g** A closer look at the bundles. **d**, **h** Single *M. vaginatus* thricomes under the compound microscope (× 100) for preliminary identification, before corroborating their identity by 16S rRNA gene typing. FB, Fort Bliss—hot desert; HSN, Hill Sandy soil—cold desert

**Fig. 2:**
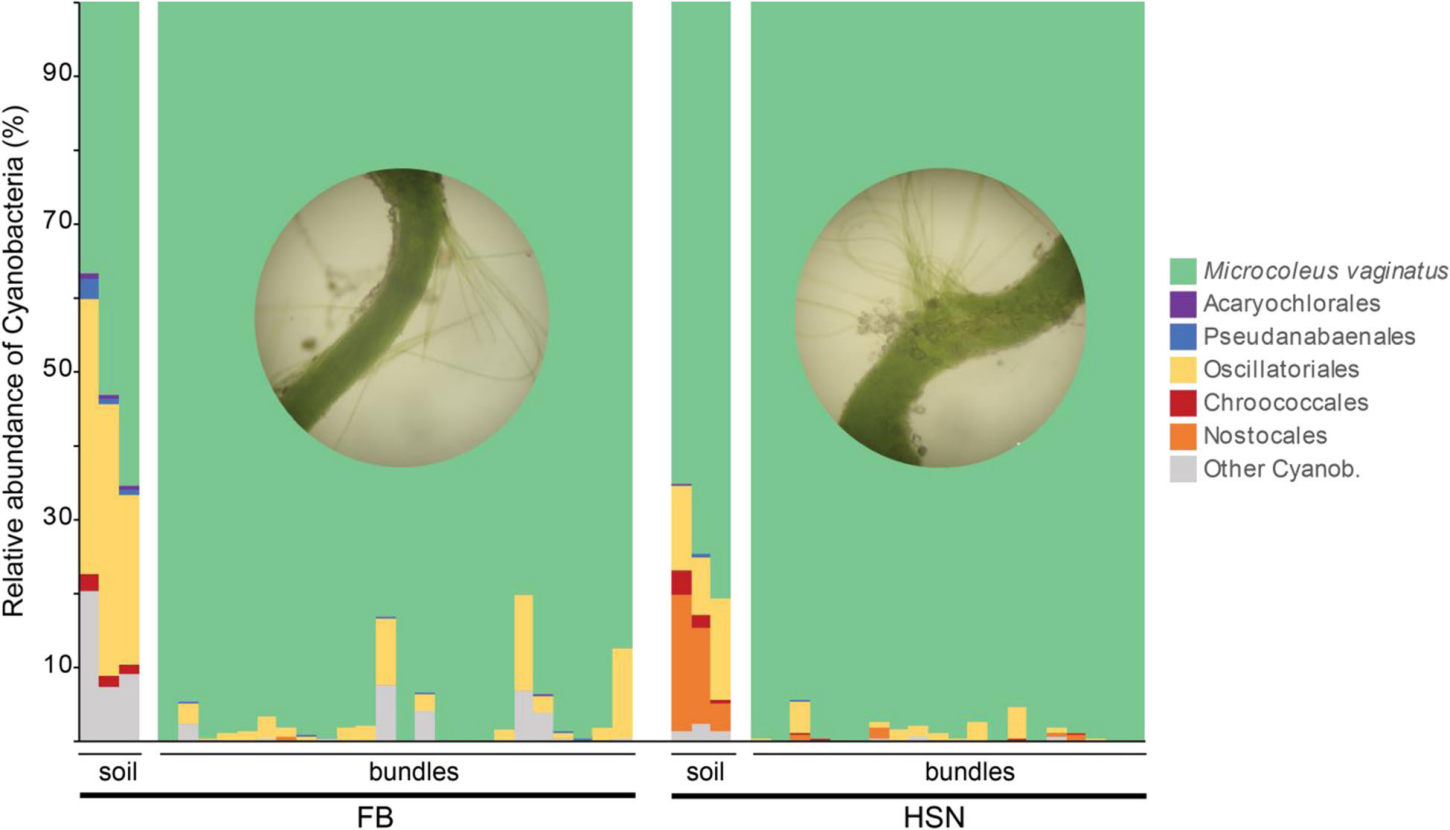
Cyanobacterial community structure and bundle identification. Relative abundance of cyanobacteria based on high-throughput sequence of 16S rRNA genes and bioinformatics analysis in *M*. *vaginatus* bundles and bulk biocrust soil from each location. Three OTUs belonging to *M*. *vaginatus* constituted the most abundant cyanobacterium in the community and the overwhelming majority of the cyanobacteria in the excised bundles. FB, Fort Bliss—hot desert; HSN, Hill Sandy soil—cold desert

We carried out our analyses in samples from two contrasted geographical locations, one from the warm Chihuahuan Desert (Fort Bliss or FB) and one from the cold Great Basin Desert (Hill Sandy or HSN) (Fig. 1). The two sites and their soils and biocrusts are fully described elsewhere [35]. After excising and isolating single bundles of *M. vaginatus* from the soil, we analyzed the microbiome tightly associated with them using high-throughput 16S rRNA gene amplicon sequencing and compared using bioinformatics the composition of the microbial community intimately associated with these bundles (*n* = 44) to the total biocrust community analyzed separately (*n* = 6) (Additional file 2: Table S1), as the simplest assessment of spatial organization: close to and away from *M*. *vaginatus*. In a first check, we made sure that our original microscopic assignment of the bundles to *M*. *vaginatus* was correct, as other bundle-forming cyanobacterial species populate biocrusts (Fig. 2). This was indeed the case. We then compared the composition of the rest of the microbiomes (to the exclusion of all OTU’s attributable to *M*. *vaginatus*). We found that overall the bundle OTU richness (average chao1 202 ± 97) was an order of magnitude lower than the richness of the total biocrust community (average chao1 2107 ± 320). While the OTU richness of bundles was not different between locations, the HSN site biocrust community was significantly more diverse (average chao1 2432 ± 56) than that of the FB site (average chao1 1801 ± 115) (Additional file 2: Table S1).

**Fig. 3:**
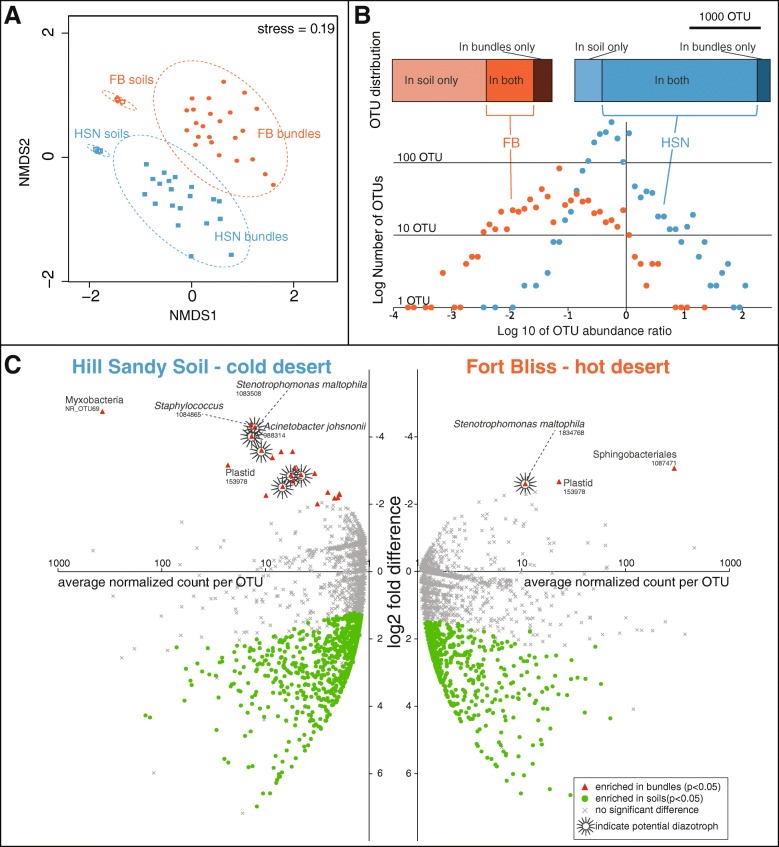
Spatial separation of microbial types close to, and away from, *M*. *vaginatus* in soil crusts. **a** NMDS ordination of Bray-Curtis pairwise distance computed on the Hellinger-transformed OTU composition in bulk soil or *M*. *vaginatus* cyanospheres (sans *M*. *vaginatus*), with 95% confidence ellipses drawn for each with a stress value of 0.19. In each setting, bulk soil communities differ in composition from their respective *M*. *vaginatus* cyanosphere (bundle communities). FB, Chihuahuan Desert (hot desert); HSN, Great Basin Desert (cold desert). **b** Frequency distribution of the ratios in relative abundance for microbial OTUs that co-occurred in the cyanospheres of *M*. *vaginatus* and in the bulk soil crusts, showing a skewed distribution towards segregation. **c** Differential abundance of microbial OTUs (sans *M. vaginatus*) in the cyanosphere vs. bulk soil crust community assessed with the DESeq2 method for cold and hot desert locations. For each OTU, the average normalized counts are plotted against their differential abundance. OTUs that were differentially abundant (*p* < 0.05) are represented as solid triangles and circles, while cross symbols denote those with non-significant preference. Negative values indicate enrichment in the cyanosphere and positive in the bulk soil crust

A non-metric multidimensional scaling (NMDS) ordination of the beta diversity Bray-Curtis metric on the Hellinger-transformed OTU table (Fig. 3a) revealed that the composition of the bundle communities was distinct from those of their respective biocrust soil community of origin (Adonis, *F* = 4.7, *p* value = 0.001), forming a compositional “cyanosphere” (by analogy to the plant rhizosphere). The cyanosphere composition was also differentiated according to the sampling location (Great Basin or Chihuahuan Desert).

In order to further probe the factors driving the differentiation between cyanosphere and biocrust microbiome, we calculated the ratio of abundance of each operational taxonomic unit (OTU) in the bundles vs. the bulk soil, for those OTUs that were detected in both settings (669 shared OTUs at FB, and 2177 shared OTUs at HSN). The frequency distribution of these ratios was clearly skewed towards negative values (Fig. 3b), implying that many more microbial types tended to segregate away from *M*. *vaginatus* than tended to aggregate within its cyanosphere. In order to identify the OTUs involved in this spatial organization, we used the DESeq2 method [36], which computes statistical significance for differential distributions of OTUs between two possible outcomes. Twenty OTUs in the cold desert cyanospheres (HSN) and two OTUs in those from the hot desert (FB) could be classified with statistical confidence (*p* < 0.05; listed in Additional file 1: Tables S2 and S3, respectively), as consistent *M*. *vaginatus* close neighbors across different bundles, while 758 OTUs (HSN) and 592 OTUs (FB) were statistically more abundant away from it (Fig. 3c; listed in Additional file 3: Table S4). This analysis confirmed that the significant difference between the cyanosphere and the total soil community is driven by a small number of bacteria associated with *M*. *vaginatus* bundles (aggregating OTUs), while there are large numbers of bacteria (segregating OTUs) that were preferentially found away from them, as part of the bulk soil*.* Accounting for the relative contribution of each OTU, we could compute that altogether more than two thirds of all the biocrust bacteria were significantly affected in their spatial distribution by the presence of *M*. *vaginatus* (Table 1, Fig. 3c), the large majority segregating away from the cyanosphere.

**Table 1:** Bacterial population size (as percentage of total 16S rRNA gene reads) of bacteria that show spatial responsiveness to *M*. *vaginatus*. Aggregating and segregating OTUs were determined statistically as per Fig. 1c, each OTU was then weighed by its relative abundance, and all contributions added

	FB soils	HSN soils
Aggregating	0.22	0.13
Segregating	52.55	69.97
Non-significant	47.23	29.89

From the 5 negative control samples (sterilized sewing cotton thread) that we analyzed in the same way in an effort to account for any external contamination (i.e., operator or environmental source) during our handling of bundle samples, we recovered a total of 92 OTUs, among which 4 matched (> 99% sequence similarity) 1 of our aggregating OTUs (Additional file 4: Table S5). A conservative take of this result is that they are all contaminants. However, 1 out of these 4 OTUs has been detected by other methods as one of the most common heterotrophic nitrogen fixers in early biocrust stages [33]. The same OTU matches (100%) a culture recently isolated from *M*. *vaginatus* bundles in nitrogen-free media (Nelson et al., unpublished data). This suggests that we may not have the taxonomic resolution to resolve the true status of these OTUs and therefore decided not to filter out these 4 OTUs, but rather to flag them in Additional file 4: Table S5.

### The *M*. *vaginatus* cyanosphere is enriched in nitrogen-fixing members

**Fig. 4:**
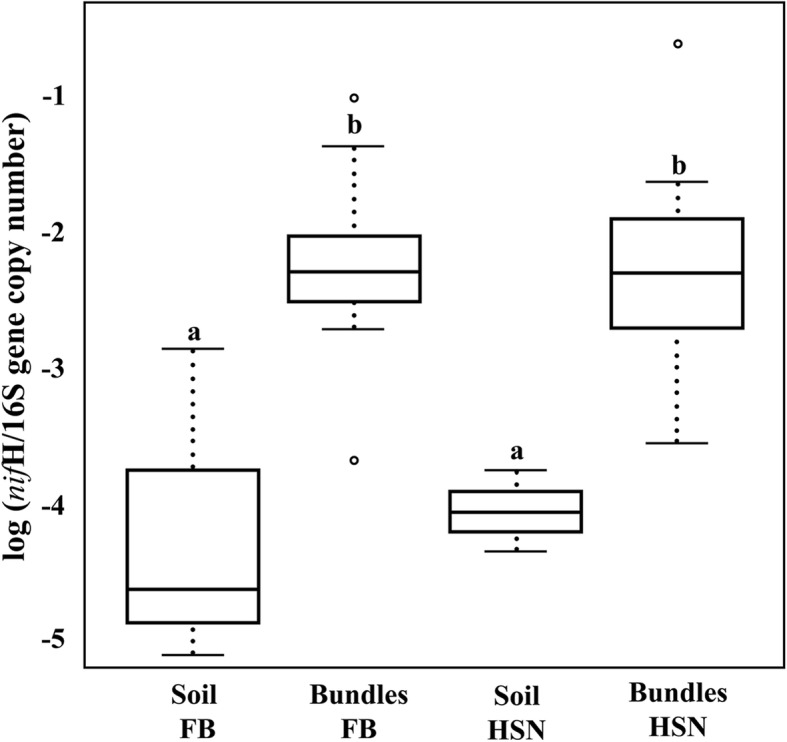
Ratio of the *nif*H to 16S rRNA gene copy number in bulk biocrust soil and *M*. *vaginatus* bundle (cyanosphere) communities. The *nifH*/16S rRNA ratio was obtained by quantitative PCR assays of each and was two to three orders of magnitude higher in the cyanosphere than in the bulk soil crust. A one-way ANOVA test showed that differences between groups (*M*. *vaginatus* bundles vs. bulk biocrust soil) were significant (*p* value < 0.005)

We further analyzed the identity of the 21 OTUs that were statistically bona fide cyanosphere members using a refined phylogenetic placement in search for functional inference (the “Methods” section, Additional file 1: Tables S2 and S3, and Additional file 3: Table S4). We found that all taxonomically assignable OTUs could be inferred to be from copiotrophic bacteria, which are rather uncommon in organic-poor desert soils and otherwise typical of organic-rich rhizospheres, animal microbiomes, or dung (among them several enterobacteria, pseudomonads, *Streptococcus*, *Bacteroides*, and *Myxobacteria*; Additional file 1: Tables S2 and S3 and Additional file 2). We also found that at least 6 OTUs from those 21 could be inferred by phylogenetic placement to be likely members of N_2_-fixing clades (Additional file 1: Tables S2 and S3 and Additional file 2). Three of these OTUs (assigned to *Escherichia/Shigella*, *Acinetobacter*, and *Stenotrophomonas*) matched (> 99%) 3 of the phylotypes identified elsewhere as important heterotrophic diazotrophs of biocrusts through ^15^N-DNA SIP and genomic analyses [33]. This suggests that diazotrophic capacity may be a common denominator of the cyanosphere community. In order to gauge the relative potential for N_2_ fixation of the cyanosphere community more directly, we performed quantitative PCR to determine the ratio of *nif*H genes (coding for a nitrogenase subunit) to 16S rRNA copy numbers existing in the bundle cyanosphere vs. that in the bulk biocrust microbiome. We found that the *nif*H gene was some 100-fold more abundant in the cyanosphere of *M*. *vaginatus* bundles (Fig. 4) than in the bulk soil crusts, regardless of geographic origin.

### Oligotroph, phototroph, and autotroph members among those segregated from *M*. *vaginatus*

We again used phylogenetic placement on the 1350 soil OTUs that were significantly more abundant away from *M*. *vaginatus* bundles, in an attempt to refine their potential function. Since most microbial taxonomic diversity is not well described functionally, we could not find relevant inferences for the majority of these OTUs, which prevented us from carrying out a fully quantitative estimation. Instead, we asked specific hypotheses based on logical predictions. A simple such prediction would be that competition for light may drive other phototrophs away from the dominant *M*. *vaginatus*. Indeed, no other known phototrophs were found among aggregating bacteria and all bona fide phototrophs were among the segregating OTUs, including other cyanobacteria, proteobacterial purple non-sulfur phototrophs, and several *Chloroflexi*. In a similar manner, one could predict that competition for CO_2_ would tend to segregate other autotrophs from *M*. *vaginatus*, which was again the case (including all other photoautotrophs like cyanobacteria, purple non-sulfurs, some Chloroflexales, as well as nitrifying chemolithoautotrophic Archaea and Bacteria, such as *Nitrososphaera* and *Nitrospira*). A final case could be made on the basis of the fact that bacteria in the cyanosphere tend to gather uncommon copiotrophs (such as enterobacteria, pseudomonads, *Streptococcus*, *Bacteroides*, and *Myxobacteria*; Additional file 1: Tables S2 and S3), so it is possible that oligotrophs grow better away from the sources of leaking photosynthate that *M*. *vaginatus* represents. Our analysis revealed that members of well-known oligotrophic bacterial genera (*Caulobacter*, *Asticcacaulis*, *Brevundimonas*, and *Sphingomonas* in the Proteobacteria; *Modestobacter*, *Blastococcus*, *Geodermatophilus*, *Nocardioides*, and *Arthrobacter* in the Actinobacteria; *Fimbriimonas*, *Chthonomonas*, and *Armatimonas* in the Armatimonadetes; and *Longimicrobium* in the Gemmatimonadetes) were preferentially represented among the segregating microbiome fraction, but absent from the cyanosphere (Additional file 1: Tables S2 and S3 and Additional file 3: Table S4).

## Discussion

### The cyanosphere as a differentiated compartment of the biocrust microbiome

We could show that the community closely associated to *M*. *vaginatus* bundles, while containing many of the same microbial OTUs found in the bulk biocrust soil, differs from it in that it attracts a specific set of bacteria that are otherwise quite rare. This phenomenon is not unlike microbial hotspots that are found around plant roots in the soil [37], and so we called this specialized community the *cyanosphere*. This is consistent with the developing notion of an evolutionarily deeply rooted continuum of specific interconnections between phototrophic and heterotrophic systems, from “algal spheres” to root microbiomes [38]. Interestingly, all OTUs that define the *M*. *vaginatus* cyanospheres would belong to the “rare biosphere” [39] by virtue of their extremely low abundance in the biocrust microbiome (the median rank of aggregating OTUs in soils was 2549th), and yet they may be playing significant functional roles in biocrust systems.

The cyanosphere compartment possesses differential features that might explain why a specific set of bacteria thrive in it, compared to the rest of the biocrust soils. First, it is an organic carbon hotspot based on the high concentration of the extracellular polymeric substances (EPS) that make up a bundle’s sheath [40, 41] and by the dynamic excretion of a large variety of small molecular weight organics by *M*. *vaginatus* cells [30]. The EPS sheath likely offers means for physical anchoring of bacteria and might help retain hydration water during desiccation [42]. Altogether, the cyanosphere likely constitutes top real estate within the biocrust where occupancy might be determined by microbe-microbe competition for this resource-rich hotspot [43].

### *M*. *vaginatus*’ cyanosphere may be at least partly based on a mutualistic C for N exchange

Clearly, the abundance of nitrogenase *nif*H gene in the cyanosphere is roughly 100-fold higher than that in the bulk crust soil, which strongly suggests that nitrogen fixation “concentrates” there, a fact supported by the high abundance of typical nitrogen-fixing taxa among cyanosphere members. We therefore propose that there must exist an active mutualistic relationship established between the diazotrophic copiotrophic heterotrophs and *M*. *vaginatus* based on a C for N exchange. Proof of such a symbiotic relationship will necessitate the deployment of alternative approaches, which could include using ^13^CO_2_/^15^N_2_ stable isotope tracers in combination with NanoSIMS imaging for direct visualization of a coupled exchange [44], or, even more directly, the reconstitution of the mutualistic relationship from representative isolates of each partner. Unfortunately, no cultured representatives are yet available of these heterotrophic diazotrophs. Chemical characterization of the C-compound used by the N-fixing heterotrophs and their consumption spectrum by other biocrust organisms [40] would allow to determine how targeted and precisely controlled this C to N exchange might be.

In any event, the fact that nitrogen fixation rates do not differ significantly between early-stage and mature biocrusts [15] illustrates the critical role that these heterotrophic diazotrophs may play in the establishment and early development of biocrusts. That *M*. *vaginatus* carries its own built-in nitrogen fixation “microbiome module” must offer it very significant fitness value as a colonizer of N-depleted soils. In a way, it is *M*. *vaginatus* plus its cyanosphere that constitutes the true pioneer of biocrust. As such, it should prove interesting to target the use of mixed cultures in current efforts for arid land soil rehabilitation in which inoculation and survival of *Microcoleus vaginatus* is key [45].

### A spatially organized microbiome

It seems from our results that the powers for spatial organization of the biocrust microbiome by *M*. *vaginatus* may not be relegated to the formation of a cyanosphere, but potentially extend to a significant proportion of the community that segregates from it. Our effort to interrogate the putative function of those segregating OTUs showed that competitors for light and for CO_2_ predictably count among them, as did members of typically oligotrophic bacterial groups, as one would have expected. However, given that a large fraction of the segregating OTUs could not be confidently functionally assigned, it is premature to conclude that such distribution patterns based on competition could hold for all. Our knowledge of the principles of microbiome assembly has clearly lagged behind a bewildering advance of the technological ability to describe in detail their complex composition and potential capabilities through “omics” techniques [46]. The use of network theory and analysis has been at the forefront of such efforts [43, 47, 48]. At the base of network studies is the assumption that functional interactions among microbial types are the main drivers of spatial patterns of occurrence, such that detection of microbial co-occurrence can reveal essentially functional networks. This is of course true for cases of obligate, strong interactions like symbioses, which tend to promote the formation of tight, microscale consortial aggregates [49]. Theoretical and experimental work points to subtler nutrient gradients as crucial to the maintenance of spatially structured microbiomes [50, 51]. If this were correct, one would expect that microbial species that are functionally central in a microbiome will play an inordinately large role on the spatial structuring of the rest of the components (i.e., they will effectively landscape the microbiome) through metabolic interactivity. This is precisely what our results seem to imply. Our observations provide a first glimpse at the fact that spatial organization of microbiomes might further constrain and be constrained by metabolic interactivity.

## Conclusions

We physically isolated *M*. *vaginatus* bundles from the biocrusts they form, taking advantage of their large size, and analyzed the composition of the microbial communities that develop in its close proximity. We found that a diverse set of bacteria inhabit the cyanosphere compartment (202 ± 97 OTUs) among which a small fraction (21) are significantly more abundant aggregated with *M*. *vaginatus*, compared to the bulk soil, and that a large number of OTUs significantly tend to segregate from *M*. *vaginatus*. Phylogenetic placements suggest that all members of the cyanosphere are copiotrophs and many are diazotrophs. By contrary, competition for light, CO_2,_ and low carbon concentrations define at least a part of the OTU that segregate from it. The qPCR assay for nifH strongly suggests that the inhabitants of the cyanosphere also concentrate the nitrogen fixation function in the biocrust. We propose that there exist a mutualism between *M*. *vaginatus* and copiotrophic diazotrophs in its cyanosphere and that this consortium constitutes the true pioneer community enabling the colonization of nitrogen-poor soils.

## Methods

### Sample collection and bundle picking

We studied biocrusts from two locations in the Southwestern US: the Chihuahuan Desert (near El Paso, TX; 32.431069–105.984151°) and the Great Basin Desert (near Salt Lake City, UT; 32.54558–106.72324°). The sampling locations have been fully described in Velasco Ayuso et al. [35] and Giraldo Silva et al. [45]. Biocrusts were wetted in situ with distilled water for sampling, then dried, and stored in dark and dry conditions until experimentation, when they were wetted for 24 h prior to sampling. Using forceps under a dissection scope, we picked *M*. *vaginatus* bundles from each site (Additional file 2: Table S1, Additional file 5: Video S1), which were then individually washed in autoclaved Milli-Q water, and observed under the microscope to assign species. Five pieces of autoclaved sewing thread, used to mimic *M*. *vaginatus* bundles, were subjected to the same procedure and used as negative controls. For the respective bulk soil crusts, we sampled in triplicate (6 samples total) taking 0.5 cm deep and 1 cm (internal diameter) cores (Additional file 2: Table S1). Each bulk soil, bundle, or control (sewing thread) was transferred to 2-mL tubes containing SDS, and DNA was extracted immediately.


**Additional file 5: Video S1.** Manipulative isolation of *M. vaginatus* bundles under the dissection microscope. (MP4 17474 kb)


### DNA purification, 16S library preparation, and sequencing

DNA from all samples was isolated using a PowerSoil DNA Isolation Kit (MoBio, Carlsbad CA), following the manufacturer’s protocol. General prokaryotic primers targeting the 16S rRNA V4 region: 515F 5′GTGCCAGCMGCCGCGGTAA-3′ and, 806R 5′-GGACTACHVGGGTWTCTAAT-3′ [52] were used for library preparation. PCR was performed in triplicate, and products pooled for each sample, with an initial phase of denaturation at 94 °C for 3 min, followed by 35 cycles (denaturation 64 °C for 45 s, annealing 50 °C for 50 s, extension 72 °C for 90 s), followed by a final extension phase at 72 °C for 10 min. Determination of total DNA concentrations in PCR products was assessed by Quant-iT PicoGreen dsDNA Assay Kit (Life Technologies, New York, USA) and pooled to a total concentration of 240 ng of DNA per sample in the library. DNA was cleaned using the QIA Quick PCR Purification kit (QIAGEN, Valencia, CA, USA). The library DNA concentration was quantified using the Kit ABI Prism® (Kapa Biosystems, Wilmington, MA, USA), following the manufacturer’s instructions, diluted to a final concentration of 4 nM, then denatured and diluted to a final concentration of 4 pM, spiked with a 30% PhiX solution, and then loaded on the MiSeq Illumina Sequencer (Illumina, San Diego, CA, USA). The sequencing was performed in the Microbiome Analysis Laboratory at Arizona State University (Tempe, AZ, USA), using custom primers, paired ends sequencing, and default chemistry.

### Quantitative PCR

Real-time polymerase chain reaction (qPCR) was used to quantify gene copy numbers of 16S rRNA and *nif*H genes in bulk soil crust and *M*. *vaginatus* bundles, using appropriate standard primers (respectively: 338F 5′-ACTCCTACGGGAGGCAGCAG-3′ 518R 5′-GTATTACCGCGGCTGCTGG-3′ [53] and PolF 5′-TGCGAYCCSAARGCBGACTC-3′ PolR 5′-ATSGCCATCATYTCRCCGGA-3′) [54]. The expected size of the amplicon was ~ 180 bp and ~ 340 bp for the 16S rRNA gene and *nif*H gene respectively. Two standard curves were made using gBlocks® Gene Fragments from Integrated DNA Technologies. The 16S rRNA gene standard curve used a dilution series from 10^7^ to 10 gene copy numbers, while for the *nif*H gene, the dilution series was from 10^4^ to 1 copy. For both assays, the reactions were prepared in triplicate in a final volume of 20 μl. Each reaction contained 5 μl of template DNA, 10 μl of Sybr Mix Green (TaqMan®), 0.4 μl of primers (500 nM for each), and 4.6 μl of water. Two negative controls were used, one with no template and one with no primers. The samples were amplified and quantified using an ABI7900HT thermocycler. The protocol for the 16S rRNA PCR included an initial denaturation phase (98 °C for 2.00 min), followed by 40 cycles of a second phase (95 °C for 10 min and finally, 55 °C for 30 min), and then a dissociation stage (beginning at 55 °C and ending at 95 °C with a 2% ramp rate) [18]. For the *nif*H gene assay [55], PCR involved an initial denaturation stage (95 °C for 3 min), followed by 45 cycles of 95 °C for 10 min and 59 °C for 30 min, and then a dissociation stage beginning at 59 °C and ending at 95 °C with a 2% ramp rate*.* The *nif*H/16S rRNA gene ratio was calculated from values of copy number per nanogram of DNA*.* The final dataset was log transformed to comply with the normality (Shapiro-Wilk test) and variance homogeneity (Levene’s test) requirement of a one-way ANOVA test. This test was run to test whether the bundle and soil groups from both FB and HSN locations had different *nif*H/16S rRNA gene ratios.

### Bioinformatics analyses

The raw FastaQ file was multiplexed within the MiSeq Illumina workflow under default parameters. Retrieved sequences were paired using PANDAseq [56] with an alignment threshold score of 0.95. High-quality sequences (length > 200 bp, minimum average Phred score 25) were further assigned to individual samples, and barcodes were removed using the Qiime 1.8 [57] *split_librairies.py* script. The master file created was used to pick operational taxonomic units (OTUs) using the *pick_open_reference_otus.py* pipeline in Qiime under default parameters. More specifically, we used the UCLUST algorithm [58] to pick OTUs at a 97% similarity threshold and assigned taxonomy using the rdp [59] classifier against the Greengenes reference database release 13.5 [60] (Additional file 1: Table S5). The OTU table produced was filtered to remove rare OTUs including potential chimeras, and only OTUs shared by at least 3 samples in the dataset were kept. Overall, these steps filtered out 5% of the total sequence count and 70% of the OTU count. All sequences attributable to *Microcoleus vaginatus* (see the “Phylogenetic analyses” section for taxonomic assignments) were removed from the OTU table. The *M*. *vaginatus*-free table was Hellinger normalized using the decostand script of the R vegan package. Beta diversity Bray-Curtis pairwise distances were calculated on the Hellinger-transformed matrix and further ordinated using NMDS in Qiime (NMDS coordinates can be found in Additional file 6: Table S6). The significance of differential OTU distribution between bundles vs. bulk soil crust was assessed using an Adonis test on the Bray-Curtis distance matrix with the *compare_categories.py* Qiime script. We further determined which OTUs were differentially abundant in the bundles vs. total community using the DeSeq2 method [36]. After checking the good agreement between the fit line and the shrinked data on the dispersion plot, a Wald test was applied to each OTU to reject the null hypothesis (*p* value < 0.05) that the logarithmic fold change between communities (i.e., in our case bundle vs. bulk soil crust) for a given OTU is null. The 5 control samples (sewing thread) were analyzed the same way in an effort to account for any external contamination (i.e., operator or environmental source) in our bundle sample handling.

### Phylogenetic analyses

Phylogenetic placement of the 21 aggregating and 1160 segregating OTU sequences was resolved by constructing 16 trees encompassing their phylogenetic diversity. For all but the cyanobacteria tree, the dataset used was a combination of our sequences along with their first Blastn hit and the closest cultured relative downloaded from SILVA rRNA database project and the NCBI 16S ribosomal RNA sequences (see supplementary OTU_classifier.ipynb). Each phylum-level dataset was then treated independently. Sequences were aligned with SSU-ALIGN [61], using a profile-based alignment strategy, in which each target sequence is aligned independently to a covariance model that uses the 16S rRNA gene secondary structure. Poorly aligned columns were removed from the alignment based on a 95% confidence profile calculated within SSU-ALIGN. The alignment was trimmed to coordinates on Geneious version 8.0 [62], so all sequences in the alignment will begin and end at the same positions. Tree topology was inferred on the CIPRES high-performance computing cluster [63], using the RAxML-HPC2 [64] workflow on XSEDE with the ML + Thorough bootstrap (1000 bootstraps) method and the GTRGAMMA model. For the cyanobacteria tree, all 16S rRNA gene sequences of at least 1100 bp were manually downloaded from NCBI [65]. A reference alignment was built from these 1034 high-quality sequences using SSU-ALIGN [66]. The reference cyanobacteria tree (https://github.com/FGPLab/cydrasil/tree/0.22a) was constructed on the CIPRES high-performance computing cluster [63], using the RAxML-HPC2 [64] workflow on XSEDE with the ML + Thorough bootstrap (1000 bootstraps, GTRGAMMA model). Cyanobacteria OTU sequences were aligned to the reference alignment with PaPaRa [67] using a probabilistic gap model and then placed into the reference tree using the RaxML8 Evolutionary Placement Algorithm [68]. Additionally, the RaxML8 Evolutionary Placement Algorithm [68] was used for some of the previously constructed trees (Acidobacteria, Deinococcus-Thermus, Armatimonadetes, Chlorobi, Chloroflexi, Firmicutes, Planctomycetes, and Verrucomicrobia) in an effort to taxonomically assign as many OTUs as possible. The resulting trees were imported into the iTOL 3 server [69] and can be visualized at http://itol.embl.de/shared/microbiomelandscaper; aggregating sequences are shown in red while segregating sequences are in blue.

## Additional files

Additional file 1: **Table S2.** Taxonomic assignments and functional inference based on phylogenetic placement for aggregating (cyanosphere) OTUs in the cold desert (HSN). **Table S3.** Taxonomic assignments and functional inference based on phylogenetic placement for aggregating (cyanosphere) OTUs in the hot desert (FB). (DOCX 38 kb)

Additional file 2: **Table S1.** Summary of SSU rRNA gene libraries analyzed from HSN and FB sample set and associated coverage and α-diversity indices. (DOCX 23 kb)

Additional file 3: **Table S4.** Taxonomic assignment and functional inferences based on phylogenetic placement for segregating OTUs for both bulk soils (cold and hot deserts). Rows colored in yellow correspond to OTUs for which inferred function was consistent with segregation from *M. vaginatus*. (DOCX 192 kb)

Additional file 4: **Table S5.** Potential contaminants. Responding OTUs detected after amplification and sequencing of negative controls (*n* = 5) without target *Microcoleus* bundles. (DOCX 17 kb)

Additional file 6: **Table S6.** NMDS plot coordinates. The corresponding plot is displayed in Fig. 3a (DOCX 18 kb)
