# Supplementary material for: Spatial segregation of the biological soil crust microbiome around its foundational cyanobacterium, Microcoleus vaginatus, and the formation of a nitrogen-fixing cyanosphere
Source: Microbiome. 2019 Apr 3;7:55. doi: 10.1186/s40168-019-0661-2 (PMC6448292; doi:10.1186/s40168-019-0661-2)
Supplement: Supplementary file 2 — Table S2. Taxonomic assignments and functional inference based on phylogenetic placement for aggregating (cyanosphere) OTUs in the cold desert (HSN). Table S3. Taxonomic assignments and functional inference based on phylogenetic placement for aggregating (cyanosphere) OTUs in the hot desert (FB). (DOCX 38 kb) [file 40168_2019_661_MOESM1_ESM.docx]

**Supplementary Table 2. Taxonomic assignments and functional inference based on phylogenetic placement** **for** **aggregating** **(cyanosphere) OTUs in the cold desert (HSN)**.

| **Phylum** | **Deepest Taxonomic Assignment** | **OTU ID** | **Nutritional type / typical habitat** | **Reference** | **Diazotrophywithin genus** | **Reference** |
| --- | --- | --- | --- | --- | --- | --- |
| Firmicutes | *Staphylococcus* | 1084865 | Copiotrophs/ saprophytic fermentative | [1] | no |  |
| Firmicutes | *Streptoccoccus gordonii* | 1083194 | Copiotrophs/ saprophytic fermentative (largely animal, but also rhizospheres, manure) | [2] | no |  |
| Alphaproteobacteria | *Methylobacterium aerolaum* | 4323871 | Strict aerobe/ mostly copiotrophs and C1 facultative | [3] | yes | [4] |
| Betaproteobacteria | *Pelomonas saccharophila* | 1108275 | Copiotrophs/ saprophytic, aerobic, facultative lithoautotroph | [5] | yes | [5] |
| Betaproteobacteria | *Snodgrassella* | 933546 | Copiotrophs/ saprophytic microaerophillic | [6] | no | genome |
| Deltaproteobacteria | Myxobacteria (Chondromyces?) | 565046 | Aerobic copiotrophs/ saprophytic/ dung |  |  |  |
| Deltaproteobacteria | Myxobacteria | New.ReferenceOTU69 | Aerobic copiotrophs/ saprophytic/ dung | [7] | no | Kegg Pathways |
| Deltaproteobacteria | Myxobacteria | New.CleanUp.ReferenceOTU14037 | Aerobic copiotrophs/ saprophytic/ dung |  |  |  |
| Gammaproteobacteria | *Escherichia/Sighella* | 1111294 | Copiotrophs/ saprophytic facultative anaerobe | [8] | yes | ^15^ |
| Gammaproteobacteria | *Haemophilus parinfluenzae* | 865469 | Aerobic copiotrophs/animal commensal | [9] | no | KEGG Pathways |
| Gammaproteobacteria | *Acinetobacter johsnonii* | 988314 | Copiotrophs/ saprophytic obligate aerobe | [10] | yes | [11] |
| Gammaproteobacteria | *Stenotrophomonas maltophila* | 1083508 | Copiotrophs/ saprophytic/nosocomial | [12] | yes | [11] |
| Gammaproteobacteria | *Stenotrophomonas maltophila* | 1834768 | Copiotrophs/ saprophytic/nosocomial |  | yes |  |
| Gammaproteobacteria | *Moraxella* | 990864 | Aerobic copiotrophs/animal commensal | [13] |  |  |
| Bacteriodetes | *Bacteriodes vulgatus* | 589277 | Copiotrophs/ saprophytic/anaerobes, rare in soil | [14] | no | KEGG Pathways |
| Bacteriodetes | Cytophagaceae | New.CleanUp.ReferenceOTU1843 | Found in terrestrial, marine and freshwater environments | [15] |  |  |
| Bacteriodetes | Cytophagaceae | New.CleanUp.ReferenceOTU7233 |  |  |  |  |
| Actinobacteria | Actinomycetales | 937735 |  |  |  |  |
| (Cyanobacteria) | higher plant plastid | 153978 | NA (from plant roots or pollen) |  |  |  |
| (Cyanobacteria) | higher plant plastid | 1126072 | NA (from plant roots or pollen) |  |  |  |
| Unassigned |  | New.ReferenceOTU31 |  |  |  |  |
| Unassigned |  | New.CleanUp.ReferenceOTU8675 |  |  |  |  |

**Supplementary Table 3. Taxonomic assignments and functional inference based on phylogenetic placement** **for** **aggregating** **(cyanosphere) OTUs in the hot desert (FB)**.

| **Phylum** | **Deepest Taxonomic Assignment** | **OTU ID** | **Nutritional type/typical habitat** | **Reference** | **Diazotrophy within genus** | **Reference** |
| --- | --- | --- | --- | --- | --- | --- |
| Gammaproteobacteria | *Stenotrophomonas maltophila* | 1834768 | copiotrophs/ saprophytic/nosocomial | [12] | yes | [11] |
| Bacteriodetes | Sphingobacteriales | 1087471 |  |  |  |  |
| (Cyanobacteria) | higher plant plastid | 153978 | NA (from plant roots or pollen) |  |  |  |

**References**

1. Götz F, Bannerman T, Schleifer K-H. The Genera Staphylococcus and Macrococcus. In: Dworkin M, Falkow S, Rosenberg E, Schleifer K-H, Stackebrandt E, editors. Prokaryotes Vol 4 Bact Firmicutes, Cyanobacteria. New York, NY: Springer-Verlag Berlin Heidelberg; 2006. p. 5–75. Available from: https://doi.org/10.1007/0-387-30744-3_1

2. Smith DL, Lamont JR, Wilkins O, Bywater-ekeg M. From yogurt to yield : Potential applications of lactic acid bacteria in plant production. Soil Biol Biochem. 2017;111:1–9.

3. N. Green P. Methylobacterium. In: Dworkin M, Falkow S, Rosenberg E, Schleifer K-H, Stackebrandt E, editors. Prokaryotes Vol 5L Alpha Beta Subclasses. New York, NY: Springer-Verlag Berlin Heidelberg; 2006. p. 257–65.

4. Jourand P, Giraud E, Béna G, Sy A, Willems A, Gillis M, et al. Methylobacterium nodulans sp. nov., for a group of aerobic, facultatively methylotrophic, legume root-nodule-forming and nitrogen-fixing bacteria. Int J Syst Evol Microbiol. 2004;54:2269–73.

5. Barraquio WL, JR BCP, Watanabe I, Knowles R. Nitrogen Fixation by Pseudomonas saccharophila Doudoroff ATCC 15946. J Gen Microbiol. 1986;132:237–41.

6. Kwong WK, Moran NA. Cultivation and characterization of the gut symbionts of honey bees and bumble bees: Description of Snodgrassella alvi gen. nov., sp. nov., a member of the family Neisseriaceae of the betaproteobacteria, and Gilliamella apicola gen. nov., sp. nov., a memb. Int J Syst Evol Microbiol. 2013;63:2008–18.

7. Dawid W. Biology and global distribution of myxobacteria in soils. FEMS Microbiol Rev. 2000;24:403–27.

8. Octavia S, Lan R. The Family Enterobacteraceae. In: Rosenberg E, DeLong EF, Lory S, Stackebrandt E, Thompson F, editors. The Prokaryotes: Gammaproteobacteria. 4th ed. London: Springer-Verlag Berlin Heidelberg; 2014. p. 226–73.

9. Christensen H, Kuhnert P, Norskov-Lauritsen N, Planet PJ, Bisgaard M. The Family Pasteurellaceae. In: Rosenberg E, DeLong EF, Lory S, Stackebrandt E, Thompson F, editors. The Prokaryotes: Gammaproteobacteria. 4th ed. London: Springer-Verlag Berlin Heidelberg; 2013. p. 535–56.

10. J. Towner K. The Genus Acinetobacter. In: Dworkin M, Falkow S, Rosenberg E, Schleifer K-H, Stackebrandt E, editors. Prokaryotes Vol 6 ProteobacteriaGamma Subclass. New York, NY: Springer-Verlag Berlin Heidelberg; 2006. p. 746–58.

11. Liba CM, Ferrara FISS, Manfio GP, Fantinatti-Garboggini F, Albuquerque RC, Pavan C, et al. Nitrogen-fixing chemo-organotrophic bacteria isolated from cyanobacteria-deprived lichens and their ability to solubilize phosphate and to release amino acids and phytohormones. J Appl Microbiol. 2006;101:1076–86.

12. Ryan RP, Monchy S, Cardinale M, Taghavi S, Crossman L, Avison MB, et al. The versatility and adaptation of bacteria from the genus Stenotrophomonas. Nat Rev Microbiol. 2009;7:514–25. Available from: http://www.nature.com/doifinder/10.1038/nrmicro2163

13. Martinis Teiceira L, Carreira Merquior VL. The Family Moraxallaceae. In: Rosenberg E, DeLong EF, Lory S, Stackebrandt E, Thompson F, editors. The Prokaryotes: Gammaproteobacteria. London; 2014. p. 443–76.

14. Jeffrey Smith C, R. Rocha E, Paster B. The Medically Important Bacteroides spp. in Health and Disease. In: Dworkin M, Falkow S, Rosenberg E, Schleifer K-H, Stackebrandt E, editors. Prokaryotes Vol 7 Proteobacteria delta, esilon subclass. New York: Springer-Verlag Berlin Heidelberg; 2006. p. 381–427.

15. Mcbride MJ, Liu W, Xuemei L, Zhu Y, Zhang W, Lu X, et al. The Family Cytophagaceae. In: Rosenberg E, DeLong EF, Lory S, Stackebrandt E, Thompson F, editors. Prokaryotes Other Major Lineages Bact Archaea. 4th ed. London: Springer-Verlag Berlin Heidelberg; 2014. p. 577–93. Available from: http://link.springer.com/10.1007/978-3-642-38954-2
