# Supplementary material for: Spatial segregation of the biological soil crust microbiome around its foundational cyanobacterium, Microcoleus vaginatus, and the formation of a nitrogen-fixing cyanosphere
Source: Microbiome. 2019 Apr 3;7:55. doi: 10.1186/s40168-019-0661-2 (PMC6448292; doi:10.1186/s40168-019-0661-2)
Supplement: Supplementary file 3 — Table S1. Summary of SSU rRNA gene libraries analyzed from HSN and FB sample set and associated coverage and α-diversity indices. (DOCX 23 kb) [file 40168_2019_661_MOESM2_ESM.docx]

**Supplementary Table 1: Summary of SSU rRNA gene libraries** analyzed from HSN and FB sample set and associated coverage and α-diversity indices

|  |  |  | good's coverage | % of sequences kept through filtering | % of OTUs kept through filtering | total number of sequences analyzed | total number of OTUs | chao1 |
| --- | --- | --- | --- | --- | --- | --- | --- | --- |
| Chihuahuan desert | Bundles | FBsample01 | 99.0 | 96.1 | 75.5 | 1873 | 77 | 101.4 |
|  |  | FBsample04 | 99.5 | 98.3 | 82.2 | 9366 | 208 | 253.0 |
|  |  | FBsample06 | 97.6 | 93.6 | 83.3 | 757 | 65 | 82.0 |
|  |  | FBsample07 | 99.3 | 84.3 | 83.3 | 1030 | 45 | 48.5 |
|  |  | FBsample08 | 98.0 | 95.2 | 83.3 | 1622 | 75 | 130.1 |
|  |  | FBsample09 | 99.3 | 99.2 | 88.7 | 4180 | 141 | 160.8 |
|  |  | FBsample11 | 99.4 | 77.7 | 67.6 | 1406 | 46 | 52.0 |
|  |  | FBsample14 | 99.2 | 85.3 | 72.4 | 2624 | 89 | 136.5 |
|  |  | FBsample15 | 99.3 | 98.3 | 90.8 | 4195 | 158 | 183.2 |
|  |  | FBsample17 | 98.3 | 96.7 | 85.5 | 2439 | 141 | 189.2 |
|  |  | FBsample19 | 99.4 | 97.5 | 87.8 | 1435 | 43 | 49.0 |
|  |  | FBsample2 | 98.3 | 99.3 | 90.8 | 5493 | 227 | 348.4 |
|  |  | FBsample22 | 97.6 | 92.5 | 81.5 | 545 | 53 | 66.0 |
|  |  | FBsample24 | 99.2 | 98.4 | 86.6 | 5686 | 181 | 234.7 |
|  |  | FBsample25 | 98.5 | 98.4 | 88.2 | 1054 | 82 | 89.5 |
|  |  | FBsample26 | 99.0 | 94.9 | 84.7 | 2408 | 105 | 120.8 |
|  |  | FBsample28 | 99.2 | 98.9 | 88.0 | 6268 | 228 | 266.6 |
|  |  | FBsample29 | 98.8 | 97.1 | 84.3 | 2516 | 107 | 158.7 |
|  |  | FBsample3 | 99.6 | 99.3 | 86.8 | 12995 | 203 | 269.3 |
|  |  | FBsample30 | 96.7 | 98.6 | 93.2 | 1825 | 178 | 222.6 |
|  |  | FBsample33 | 98.3 | 96.3 | 86.3 | 2085 | 158 | 183.2 |
|  |  | FBsample35 | 98.7 | 98.0 | 86.0 | 3543 | 191 | 219.3 |
|  |  | FBsample4 | 99.4 | 95.7 | 84.0 | 3028 | 89 | 106.1 |
|  |  | FBsample5 | 99.2 | 97.6 | 85.7 | 4788 | 150 | 191.6 |
|  | Soils | FBsampleA | 98.4 | 95.2 | 68.5 | 25464 | 1293 | 1684.1 |
|  |  | FBsampleB | 95.4 | 94.0 | 72.9 | 10800 | 1200 | 1760.3 |
|  |  | FBsampleC | 98.0 | 89.3 | 56.1 | 23120 | 1616 | 1958.6 |
| Great Basin desert | Bundles | HSNsample01 | 98.9 | 99.3 | 88.5 | 6969 | 192 | 281.5 |
|  |  | HSNsample10 | 98.5 | 93.1 | 86.1 | 1744 | 118 | 147.5 |
|  |  | HSNsample11 | 98.6 | 98.7 | 87.9 | 5275 | 218 | 311.9 |
|  |  | HSNsample15 | 97.4 | 95.9 | 92.1 | 1361 | 128 | 206.8 |
|  |  | HSNsample18 | 97.2 | 99.0 | 93.7 | 2435 | 207 | 291.4 |
|  |  | HSNsample20 | 96.4 | 99.3 | 95.5 | 672 | 63 | 90.6 |
|  |  | HSNsample22 | 98.3 | 97.2 | 91.9 | 2399 | 147 | 192.6 |
|  |  | HSNsample23 | 98.1 | 98.6 | 92.3 | 3068 | 240 | 286.9 |
|  |  | HSNsample24 | 97.1 | 95.6 | 87.9 | 1387 | 131 | 209.0 |
|  |  | HSNsample25 | 99.3 | 91.0 | 73.2 | 4367 | 104 | 149.1 |
|  |  | HSNsample27 | 97.8 | 98.4 | 92.3 | 3251 | 203 | 291.1 |
|  |  | HSNsample28 | 99.4 | 97.7 | 75.6 | 9967 | 248 | 325.5 |
|  |  | HSNsample29 | 98.6 | 97.4 | 90.8 | 2841 | 108 | 154.3 |
|  |  | HSNsample34 | 99.1 | 98.6 | 89.8 | 6762 | 256 | 323.2 |
|  |  | HSNsample37 | 98.3 | 98.4 | 89.2 | 3811 | 223 | 290.3 |
|  |  | HSNsample42 | 99.4 | 98.8 | 88.0 | 17391 | 387 | 488.0 |
|  |  | HSNsample43 | 99.0 | 98.3 | 84.8 | 7662 | 291 | 343.4 |
|  |  | HSNsample44 | 99.3 | 86.4 | 72.1 | 10876 | 227 | 322.6 |
|  |  | HSNsample45 | 99.3 | 98.4 | 85.5 | 5408 | 141 | 187.3 |
|  |  | HSNsample46 | 99.6 | 99.4 | 90.3 | 6235 | 102 | 122.0 |
|  | Soils | HSNsampleA | 99.1 | 94.8 | 57.8 | 51549 | 1985 | 2336.6 |
|  |  | HSNsampleB | 99.2 | 94.3 | 55.9 | 58807 | 2113 | 2466.8 |
|  |  | HSNsampleC | 99.0 | 94.1 | 57.6 | 51940 | 2038 | 2438.2 |
