# Supplementary material for: Spatial segregation of the biological soil crust microbiome around its foundational cyanobacterium, Microcoleus vaginatus, and the formation of a nitrogen-fixing cyanosphere
Source: Microbiome. 2019 Apr 3;7:55. doi: 10.1186/s40168-019-0661-2 (PMC6448292; doi:10.1186/s40168-019-0661-2)
Supplement: Supplementary file 4 — Table S4. Taxonomic assignment and functional inferences based on phylogenetic placement for segregating OTUs for both bulk soils (cold and hot deserts). Rows colored in yellow correspond to OTUs for which inferred function was consistent with segregation from M. vaginatus. (DOCX 192 kb) [file 40168_2019_661_MOESM3_ESM.docx]

**Suplementary Table 4. Taxonomic assignment and functional inferences based on phylogenetic placement** **for segregating OTUs for both bulk soils (Cold and hot deserts).** Rows colored in yellow correspond to those OTUs for which inferred function was consistent with segregation from *M. vaginatus*.

| **Phylum** | **Deepest Taxonomic Assignment** | **OTU ID** | **Nutritional type/typical habitat** | **Reference** |
| --- | --- | --- | --- | --- |
| Acidobacteria | In the Blastocatellaceae clade (*Aridibacter*/*Blastocatella*/*Stenotrophobacter*) | 4359078 | Chemoorganoheterothrophs/Abundant in soils | [1] |
| Acidobacteria | In the Blastocatellaceae clade (*Aridibacter*/*Blastocatella*/*Stenotrophobacter*) | 830338 |  |  |
| Acidobacteria | In the Blastocatellaceae clade (*Aridibacter*/*Blastocatella*/*Stenotrophobacter*) | New.CleanUp.ReferenceOTU1120 |  |  |
| Acidobacteria | In the Blastocatellaceae clade (*Aridibacter*/*Blastocatella*/*Stenotrophobacter*) | New.CleanUp.ReferenceOTU23458 |  |  |
| Acidobacteria | In the Blastocatellaceae clade (*Aridibacter*/*Blastocatella*/*Stenotrophobacter*) | 809387 |  |  |
| Acidobacteria | In the Blastocatellaceae clade (*Aridibacter*/*Blastocatella*/*Stenotrophobacter*) | 4321498 |  |  |
| Acidobacteria | In the Blastocatellaceae clade (*Aridibacter*/*Blastocatella*/*Stenotrophobacter*) | New.CleanUp.ReferenceOTU9855 |  |  |
| Acidobacteria | In the Blastocatellaceae clade (*Aridibacter*/*Blastocatella*/*Stenotrophobacter*) | New.CleanUp.ReferenceOTU6851 |  |  |
| Acidobacteria | In the Blastocatellaceae clade (*Aridibacter*/*Blastocatella*/*Stenotrophobacter*) | 279384 |  |  |
| Acidobacteria | In the Blastocatellaceae clade (*Aridibacter*/*Blastocatella*/*Stenotrophobacter*) | 612580 |  |  |
| Acidobacteria | In the Blastocatellaceae clade (*Aridibacter*/*Blastocatella*/*Stenotrophobacter*) | 4297666 |  |  |
| Acidobacteria | In the Blastocatellaceae clade (*Aridibacter*/*Blastocatella*/*Stenotrophobacter*) | New.CleanUp.ReferenceOTU23705 |  |  |
| Acidobacteria | In the Blastocatellaceae clade (*Aridibacter*/*Blastocatella*/*Stenotrophobacter*) | New.CleanUp.ReferenceOTU583 |  |  |
| Acidobacteria | In the Blastocatellaceae clade (*Aridibacter*/*Blastocatella*/*Stenotrophobacter*) | New.CleanUp.ReferenceOTU14239 |  |  |
| Acidobacteria | In the Blastocatellaceae clade (*Aridibacter*/*Blastocatella*/*Stenotrophobacter*) | 1120231 |  |  |
| Acidobacteria | In the Blastocatellaceae clade (*Aridibacter*/*Blastocatella*/*Stenotrophobacter*) | 447341 |  |  |
| Acidobacteria | In the Blastocatellaceae clade (*Aridibacter*/*Blastocatella*/*Stenotrophobacter*) | 353816 |  |  |
| Acidobacteria | In the Blastocatellaceae clade (*Aridibacter*/*Blastocatella*/*Stenotrophobacter*) | New.CleanUp.ReferenceOTU549 |  |  |
| Acidobacteria | In the Blastocatellaceae clade (*Aridibacter*/*Blastocatella*/*Stenotrophobacter*) | 687206 |  |  |
| Acidobacteria | In the Blastocatellaceae clade (*Aridibacter*/*Blastocatella*/*Stenotrophobacter*) | 4399397 |  |  |
| Acidobacteria | In the Blastocatellaceae clade (*Aridibacter*/*Blastocatella*/*Stenotrophobacter*) | New.CleanUp.ReferenceOTU16177 |  |  |
| Acidobacteria | In the Blastocatellaceae clade (*Aridibacter*/*Blastocatella*/*Stenotrophobacter*) | New.CleanUp.ReferenceOTU13917 |  |  |
| Acidobacteria | In the Blastocatellaceae clade (*Aridibacter*/*Blastocatella*/*Stenotrophobacter*) | 726866 |  |  |
| Acidobacteria | In the Blastocatellaceae clade (*Aridibacter*/*Blastocatella*/*Stenotrophobacter*) | 4451552 |  |  |
| Acidobacteria | In the Blastocatellaceae clade (*Aridibacter*/*Blastocatella*/*Stenotrophobacter*) | 1125708 |  |  |
| Acidobacteria | In the Blastocatellaceae clade (*Aridibacter*/*Blastocatella*/*Stenotrophobacter*) | New.CleanUp.ReferenceOTU6201 |  |  |
| Acidobacteria | In the Blastocatellaceae clade (*Aridibacter*/*Blastocatella*/*Stenotrophobacter*) | New.CleanUp.ReferenceOTU1640 |  |  |
| Acidobacteria | In the Blastocatellaceae clade (*Aridibacter*/*Blastocatella*/*Stenotrophobacter*) | New.CleanUp.ReferenceOTU4511 |  |  |
| Acidobacteria | In the Blastocatellaceae clade (*Aridibacter*/*Blastocatella*/*Stenotrophobacter*) | New.CleanUp.ReferenceOTU1380 |  |  |
| Acidobacteria | In the Blastocatellaceae clade (*Aridibacter*/*Blastocatella*/*Stenotrophobacter*) | 551480 |  |  |
| Acidobacteria | In the Blastocatellaceae clade (*Aridibacter*/*Blastocatella*/*Stenotrophobacter*) | New.CleanUp.ReferenceOTU14817 |  |  |
| Acidobacteria | In the Blastocatellaceae clade (*Aridibacter*/*Blastocatella*/*Stenotrophobacter*) | 171397 |  |  |
| Acidobacteria | In the Blastocatellaceae clade (*Aridibacter*/*Blastocatella*/*Stenotrophobacter*) | 86097 |  |  |
| Acidobacteria | In the Blastocatellaceae clade (*Aridibacter*/*Blastocatella*/*Stenotrophobacter*) | 627902 |  |  |
| Acidobacteria | In the Blastocatellaceae clade (*Aridibacter*/*Blastocatella*/*Stenotrophobacter*) | 141861 |  |  |
| Acidobacteria | In the Blastocatellaceae clade (*Aridibacter*/*Blastocatella*/*Stenotrophobacter*) | 213767 |  |  |
| Acidobacteria | In the Blastocatellaceae clade (*Aridibacter*/*Blastocatella*/*Stenotrophobacter*) | 4297673 |  |  |
| Acidobacteria | In the Blastocatellaceae clade (*Aridibacter*/*Blastocatella*/*Stenotrophobacter*) | 512304 |  |  |
| Acidobacteria | In the Blastocatellaceae clade (*Aridibacter*/*Blastocatella*/*Stenotrophobacter*) | 113607 |  |  |
| Acidobacteria | In the Blastocatellaceae clade (*Aridibacter*/*Blastocatella*/*Stenotrophobacter*) | New.ReferenceOTU67 |  |  |
| Acidobacteria | In the Blastocatellaceae clade (Aridibacter/Blastocatella/Stenotrophobacter) | 211578 |  |  |
| Acidobacteria | In the Blastocatellaceae clade (Aridibacter/Blastocatella/Stenotrophobacter) | 151008 |  |  |
| Acidobacteria | In the Blastocatellaceae clade (*Aridibacter*/*Blastocatella*/*Stenotrophobacter*) | 279436 |  |  |
| Acidobacteria | In the Blastocatellaceae clade (*Aridibacter*/*Blastocatella*/*Stenotrophobacter*) | 212764 |  |  |
| Acidobacteria | *Vicinamibacter silvestris* | 811187 | Chemoorganoheterothroph/Abundant in soil | [2] |
| Acidobacteria | Sister clade to *Holophaga* | New.CleanUp.ReferenceOTU9452 | Anaerobes/Found in fresh water, sediments and soils | [3] |
| Acidobacteria | Sister clade to Solibacteraceae | 806959 |  |  |
| Acidobacteria | Sister clade to Solibacteraceae | New.CleanUp.ReferenceOTU24928 |  |  |
| Acidobacteria | Sister clade to Solibacteraceae | New.CleanUp.ReferenceOTU20608 |  |  |
| Acidobacteria | Sister clade to Solibacteraceae | New.CleanUp.ReferenceOTU616 |  |  |
| Acidobacteria | Sister clade to Solibacteraceae | 728545 |  |  |
| Acidobacteria | Sister clade to Solibacteraceae | 4339765 |  |  |
| Acidobacteria | Sister clade to Solibacteraceae | New.CleanUp.ReferenceOTU15359 |  |  |
| Deinococcus-Thermus | *Deinococcus navajonensis* | 1133399 | Aerobes/Radioresistant | [4] |
| Deinococcus-Thermus | Deinococcaceae | 1018538 |  |  |
| Deinococcus-Thermus | Sister clade to *Truepera* sp. | 2248445 | Aerobes, Chemoorganotrophs/Radioresistant | [4] |
| Deinococcus-Thermus | Sister clade to *Truepera* sp. | 266995 |  |  |
| Deinococcus-Thermus | Sister clade to *Truepera* sp. | 86556 |  |  |
| Deinococcus-Thermus | Sister clade to *Truepera* sp. | 4024547 |  |  |
| Deinococcus-Thermus | Sister clade to *Truepera* sp. | 274011 |  |  |
| Actinobacteria | Sister clade to Acidimicrobiales | 1639776 | Obligate acidophilic, oxidize ferrous iron or reduce ferric iron | [5] |
| Actinobacteria | Sister clade to Acidimicrobiales | New.CleanUp.ReferenceOTU22804 |  |  |
| Actinobacteria | Sister clade to Acidimicrobiales | New.CleanUp.ReferenceOTU15026 |  |  |
| Actinobacteria | Sister clade to Acidimicrobiales | 2345835 |  |  |
| Actinobacteria | Sister clade to Acidimicrobiales | New.CleanUp.ReferenceOTU13169 |  |  |
| Actinobacteria | Sister clade to Acidimicrobiales | New.CleanUp.ReferenceOTU18563 |  |  |
| Actinobacteria | Sister clade to Acidimicrobiales | 830889 |  |  |
| Actinobacteria | Sister clade to Acidimicrobiales | 223441 |  |  |
| Actinobacteria | Sister clade to Acidimicrobiales | 4313541 |  |  |
| Actinobacteria | Sister clade to Acidimicrobiales | 790420 |  |  |
| Actinobacteria | *Angustibacter* | New.CleanUp.ReferenceOTU17686 | Facultative anaerobes/Gram-positive | [6] |
| Actinobacteria | In the Kineosporiaceae (*Angustibacter*) | 153548 |  |  |
| Actinobacteria | *Angustibacter* | 726955 |  |  |
| Actinobacteria | *Saccharothrix* | 4417388 | Anaerobes/Gram-positive | [7] |
| Actinobacteria | *Cellulomonas* | 788268 | Anaerobes/Abundant in soils/Degrade cellulose/symbiosis with *Azotobacter* | [8] |
| Actinobacteria | *Pseudonocardia* | 829373 | Some species of *Pseudonocardia* are facultative autotrophs/ Common in soils, sediments and plant roots | [9] |
| Actinobacteria | In the Pseudonocardiaceae (*Pseudonocardia, Actinokineospora*) | 501584 |  |  |
| Actinobacteria | *Pseudonocardia* | 327290 |  |  |
| Actinobacteria | In the Pseudonocardiaceae (*Pseudonocardia, Actinokineospora*) | 918840 |  |  |
| Actinobacteria | In the Pseudonocardiaceae (*Pseudonocardia, Actinokineospora*) | 1079481 |  |  |
| Actinobacteria | In the Pseudonocardiaceae | 805717 |  |  |
| Actinobacteria | In the Pseudonocardiaceae | 823816 |  |  |
| Actinobacteria | In the Pseudonocardiaceae | 1039041 |  |  |
| Actinobacteria | In the Pseudonocardiaceae | 4332665 |  |  |
| Actinobacteria | In the Pseudonocardiaceae | 869089 |  |  |
| Actinobacteria | *Geodermatophilus* | 818388 | Oligotrophs/Found mainly in soils, water and stone surfaces | [10] |
| Actinobacteria | *Geodermatophilus* | 704830 |  |  |
| Actinobacteria | *Geodermatophilus* | 156477 |  |  |
| Actinobacteria | *Modestobacter* | 510174 |  |  |
| Actinobacteria | In the Geodermatophilaceae (*Blastococcus/Modestobacter*) | 202381 |  |  |
| Actinobacteria | In the Geodermatophilaceae (*Blastococcus/Modestobacter*) | 966091 |  |  |
| Actinobacteria | In the Geodermatophilaceae (*Blastococcus/Modestobacter*) | New.CleanUp.ReferenceOTU5044 |  |  |
| Actinobacteria | In the Geodermatophilaceae (*Blastococcus/Modestobacter*) | 2855257 |  |  |
| Actinobacteria | In the Geodermatophilaceae (*Blastococcus/Geodermatophilus*) | 11439 |  |  |
| Actinobacteria | In the Geodermatophilaceae (*Blastococcus/Geodermatophilus*) | 4299608 |  |  |
| Actinobacteria | In the Geodermatophilaceae (*Geodermatophilus*) | 11428 |  |  |
| Actinobacteria | *Oryzihumus* | 538111 | Abundant in soil and marine environments | [11] |
| Actinobacteria | In the Kineosporiaceae (*Kineosporia*) | New.CleanUp.ReferenceOTU17035 | Form spores/ Found in soil, desert sands, plant litter, stems of plants | [12] |
| Actinobacteria | In the Kineosporiaceae (*Kineosporia*) | New.CleanUp.ReferenceOTU10380 |  |  |
| Actinobacteria | In the Kineosporiaceae (*Kineosporia*) | New.CleanUp.ReferenceOTU16749 |  |  |
| Actinobacteria | In the Kineosporiaceae (*Kineosporia*) | 250148 |  |  |
| Actinobacteria | In the Kineosporiaceae (*Kineosporia*) | New.CleanUp.ReferenceOTU19427 |  |  |
| Actinobacteria | In the Kineosporiaceae (*Kineosporia*) | New.CleanUp.ReferenceOTU10429 |  |  |
| Actinobacteria | *Arthrobacter* | 1081815 | Oligotrophs/Ubiquitous/ Found in soils, predominant in non-rhizosphere samples | [13] |
| Actinobacteria | *Arthrobacter* | 929901 |  |  |
| Actinobacteria | *Arthrobacter* | 1101451 |  |  |
| Actinobacteria | In the Micromonosporaceae (*Asanoa*) | 248468 | Aerobic/ Widely distributed in soils and aquatic environments | [14] |
| Actinobacteria | In the Micromonosporaceae (*Dactylosporangium*) | New.CleanUp.ReferenceOTU19623 |  |  |
| Actinobacteria | In the Micromonosporaceae (*Dactylosporangium*) | 408093 |  |  |
| Actinobacteria | In the Micromonosporaceae (*Actinoplanes, Spirilliplanes*) | 580850 |  |  |
| Actinobacteria | In the Micromonosporaceae (*Actinoplanes,Couchioplanes*) | 688259 |  |  |
| Actinobacteria | In the Micromonosporaceae (*Actinoplanes,Couchioplanes*) | 357423 |  |  |
| Actinobacteria | In the Micromonosporaceae (*Actinoplanes,Couchioplanes*) | 249571 |  |  |
| Actinobacteria | In the Micromonosporaceae (*Actinoplanes, Micromonospora*) | 250572 |  |  |
| Actinobacteria | In the Micromonosporaceae (*Actinoplanes, Micromonospora*) | 265094 |  |  |
| Actinobacteria | In the Micromonosporaceae (*Actinoplanes, Micromonospora*) | 582813 |  |  |
| Actinobacteria | *Nocardioides* | 1126182 | Chemoorganotrophs/Aerobes/ They may adapt to oligotrophic conditions/ Found in soils and aquatic environments | [15] |
| Actinobacteria | *Nocardioides* | 919487 |  |  |
| Actinobacteria | In the Nocardioidaceae | 996116 | Chemoorganotrophs/Aerobes/ Found in soils and aquatic environments |  |
| Actinobacteria | In the Nocardioidaceae | 902698 |  |  |
| Actinobacteria | In the Nocardioidaceae | 954340 |  |  |
| Actinobacteria | In the Nocardioidaceae | 1142263 |  |  |
| Actinobacteria | In the Nocardioidaceae | New.CleanUp.ReferenceOTU16525 |  |  |
| Actinobacteria | In the Nocardioidaceae | 558911 |  |  |
| Actinobacteria | *Virgisporangium* | 564093 | Aerobes/Nitrate is reduced by all species/Found in soils and aquatic environments | [14] |
| Actinobacteria | In the Nakamurellaceae (*Nakamurella*) | New.CleanUp.ReferenceOTU4744 | Aerobes/non-spore forming/ Unclear niche occupation: possible soil or water origin organism | [16] |
| Actinobacteria | Frankiales | 254635 |  |  |
| Actinobacteria | Frankiales | 146122 |  |  |
| Actinobacteria | In the Euebyaceae | 2219500 | Aerobes/ Chemoorganotrophs | [17] |
| Actinobacteria | In the Euebyaceae | 4357799 |  |  |
| Actinobacteria | In the Solirubrobacteriales (*Solirubrobacter, Patulibacter, Conexibacter)* | New.CleanUp.ReferenceOTU10190 | Gram-positive/Mesophilic/Found in soils with generally low temperature and neutral pH | [18] |
| Actinobacteria | In the Solirubrobacteriales (*Solirubrobacter, Patulibacter, Conexibacter)* | 371783 |  |  |
| Actinobacteria | In the Solirubrobacteriales (*Solirubrobacter, Patulibacter, Conexibacter)* | New.CleanUp.ReferenceOTU20163 |  |  |
| Actinobacteria | In the Solirubrobacteriales | 873887 |  |  |
| Actinobacteria | In the Solirubrobacteriales | New.CleanUp.ReferenceOTU20865 |  |  |
| Actinobacteria | In the Solirubrobacteriales | New.CleanUp.ReferenceOTU16389 |  |  |
| Actinobacteria | In the Solirubrobacteriales | 243579 |  |  |
| Actinobacteria | In the Solirubrobacteriales | 205267 |  |  |
| Actinobacteria | In the Solirubrobacteriales | 217548 |  |  |
| Actinobacteria | In the Solirubrobacteriales | 4327844 |  |  |
| Actinobacteria | In the Solirubrobacteriales | New.ReferenceOTU32 |  |  |
| Actinobacteria | In the Solirubrobacteriales | 589372 |  |  |
| Actinobacteria | In the Solirubrobacteriales | 235943 |  |  |
| Actinobacteria | In the Solirubrobacteriales | 219818 |  |  |
| Actinobacteria | In the Solirubrobacteriales | 1132235 |  |  |
| Actinobacteria | In the Solirubrobacteriales | 217448 |  |  |
| Actinobacteria | In the Solirubrobacteriales | 946860 |  |  |
| Actinobacteria | In the Solirubrobacteriales | 2025460 |  |  |
| Actinobacteria | In the Solirubrobacteriales | New.CleanUp.ReferenceOTU13206 |  |  |
| Actinobacteria | In the Solirubrobacteriales | 925200 |  |  |
| Actinobacteria | In the Solirubrobacteriales | 111050 |  |  |
| Actinobacteria | In the Solirubrobacteriales | 824845 |  |  |
| Actinobacteria | In the Solirubrobacteriales | 1110625 |  |  |
| Actinobacteria | In the Solirubrobacteriales | 957362 |  |  |
| Actinobacteria | In the Solirubrobacteriales | 1044581 |  |  |
| Actinobacteria | In the Solirubrobacteriales | 927367 |  |  |
| Actinobacteria | In the Solirubrobacteriales | New.CleanUp.ReferenceOTU4154 |  |  |
| Actinobacteria | In the Solirubrobacteriales | New.CleanUp.ReferenceOTU13003 |  |  |
| Actinobacteria | In the Solirubrobacteriales | 864395 |  |  |
| Actinobacteria | In the Solirubrobacteriales | 799959 |  |  |
| Actinobacteria | In the Solirubrobacteriales | 864304 |  |  |
| Actinobacteria | In the Solirubrobacteriales | New.CleanUp.ReferenceOTU17467 |  |  |
| Actinobacteria | In the Solirubrobacteriales | New.CleanUp.ReferenceOTU9506 |  |  |
| Actinobacteria | In the Solirubrobacteriales | 203418 |  |  |
| Actinobacteria | In the Solirubrobacteriales | 837092 |  |  |
| Actinobacteria | In the Rubrobacterales | 653788 | *Rubrobacter* species tolerate high levels of ionizing radiation/moderately thermophilic or thermophilic/Halotolerant/ Found in hot springs, arid soils | [19] |
| Actinobacteria | In the Rubrobacterales | 814924 |  |  |
| Actinobacteria | In the Rubrobacteriales | 1032653 |  |  |
| Actinobacteria | In the Rubrobacterales | 4466061 |  |  |
| Actinobacteria | In the Rubrobacterales | New.CleanUp.ReferenceOTU4244 |  |  |
| Actinobacteria | In the Rubrobacterales | 1110235 |  |  |
| Actinobacteria | In the Rubrobacterales | 546371 |  |  |
| Actinobacteria | In the Rubrobacterales | New.CleanUp.ReferenceOTU11707 |  |  |
| Actinobacteria | In the Rubrobacterales | 562741 |  |  |
| Actinobacteria | In the Rubrobacterales | 151012 |  |  |
| Actinobacteria | In the Rubrobacterales | 257807 |  |  |
| Actinobacteria | In the Rubrobacterales (*Rubrobacter*) | New.CleanUp.ReferenceOTU6243 |  |  |
| Actinobacteria | In the Rubrobacterales (*Rubrobacter*) | New.CleanUp.ReferenceOTU19685 |  |  |
| Actinobacteria | In the Rubrobacterales (*Rubrobacter*) | New.CleanUp.ReferenceOTU10069 |  |  |
| Actinobacteria | In the Rubrobacterales (*Rubrobacter*) | 238700 |  |  |
| Actinobacteria | In the Rubrobacterales (*Rubrobacter*) | 1115272 |  |  |
| Actinobacteria | In the Rubrobacterales (*Rubrobacter*) | 256163 |  |  |
| Actinobacteria | In the Rubrobacterales (*Rubrobacter*) | New.CleanUp.ReferenceOTU1552 |  |  |
| Actinobacteria | In the Rubrobacterales (*Rubrobacter*) | 511366 |  |  |
| Actinobacteria | In the Rubrobacterales (*Rubrobacter*) | New.CleanUp.ReferenceOTU1105 |  |  |
| Actinobacteria | In the Rubrobacterales (*Rubrobacter*) | 1107601 |  |  |
| Actinobacteria | In the Rubrobacterales (*Rubrobacter*) | 673883 |  |  |
| Actinobacteria | In the Rubrobacterales (*Rubrobacter*) | 166076 |  |  |
| Actinobacteria | In the Rubrobacterales (*Rubrobacter*) | 825937 |  |  |
| Actinobacteria | In the Rubrobacterales (*Rubrobacter*) | 1117022 |  |  |
| Actinobacteria | In the Rubrobacterales (*Rubrobacter*) | 833324 |  |  |
| Actinobacteria | In the Rubrobacterales (*Rubrobacter*) | 511572 |  |  |
| Actinobacteria | In the Rubrobacterales (*Rubrobacter*) | 255018 |  |  |
| Actinobacteria | In the Rubrobacterales (*Rubrobacter*) | 587534 |  |  |
| Actinobacteria | Sister clade to *Gaiella occulta* | 3334373 | Strictly aerobes/Isolated from a very poor in mineral ions environment/Identified in soil, water distributions systems and shallow lakes | [20] |
| Actinobacteria | Sister clade to *Gaiella occulta* | 3334374 |  |  |
| Actinobacteria | Unassigned | New.CleanUp.ReferenceOTU15155 |  |  |
| Actinobacteria | Unassigned | New.CleanUp.ReferenceOTU20273 |  |  |
| Actinobacteria | Unassigned | New.CleanUp.ReferenceOTU23979 |  |  |
| Actinobacteria | Unassigned | 939546 |  |  |
| Armatimonadetes | *Fimbriimonas ginsengisoli* | New.CleanUp.ReferenceOTU21444 | Aerobic oligotrophs/Found in soils | [21,22] |
| Armatimonadetes | *Fimbriimonas ginsengisoli* | New.CleanUp.ReferenceOTU21405 |  |  |
| Armatimonadetes | Sister clade to *Chthonomonas calidirosea* | New.CleanUp.ReferenceOTU11267 | Aerobic oligotrophs/Found in soils | [22] |
| Armatimonadetes | Sister clade to *Chthonomonas calidirosea* | New.CleanUp.ReferenceOTU5198 |  |  |
| Armatimonadetes | In the Armatimonadaceae clade (*Armatimonas rosea*) | New.CleanUp.ReferenceOTU10796 | Aerobic oligotrophs/ Found in soils in close association with plant roots | [22] |
| Armatimonadetes | In the Armatimonadaceae clade (*Armatimonas rosea*) | 1112858 |  |  |
| Armatimonadetes | In the Armatimonadaceae clade (*Armatimonas rosea*) | 1113667 |  |  |
| Armatimonadetes | In the Armatimonadaceae clade (*Armatimonas rosea*) | 80475 |  |  |
| Armatimonadetes | In the Armatimonadaceae clade (*Armatimonas rosea*) | New.CleanUp.ReferenceOTU21874 |  |  |
| Armatimonadetes | In the Armatimonadaceae clade (*Armatimonas rosea*) | New.CleanUp.ReferenceOTU8905 |  |  |
| Armatimonadetes-Candidate class division FPB |  | New.CleanUp.ReferenceOTU13058 |  |  |
| Armatimonadetes-Candidate class division FPB |  | New.CleanUp.ReferenceOTU14215 |  |  |
| Armatimonadetes-Candidate class division FPB |  | New.CleanUp.ReferenceOTU17500 |  |  |
| Armatimonadetes-Candidate class division FPB |  | New.CleanUp.ReferenceOTU23745 |  |  |
| Armatimonadetes-Candidate class division FPB |  | New.CleanUp.ReferenceOTU6425 |  |  |
| Armatimonadetes-Candidate class division FPB |  | New.CleanUp.ReferenceOTU5536 |  |  |
| Armatimonadetes-Candidate class division FPB |  | 143458 |  |  |
| Armatimonadetes-Candidate class division FPB |  | New.CleanUp.ReferenceOTU11136 |  |  |
| Armatimonadetes-Candidate class division FPB |  | 1061059 |  |  |
| Armatimonadetes-Candidate class division FPB |  | New.CleanUp.ReferenceOTU4208 |  |  |
| Armatimonadetes-Candidate class division FPB |  | 3091900 |  |  |
| Armatimonadetes-Candidate class division FPB |  | New.CleanUp.ReferenceOTU8394 |  |  |
| Armatimonadetes-Candidate class division FPB |  | New.CleanUp.ReferenceOTU8264 |  |  |
| Armatimonadetes-Candidate class division FPB |  | New.CleanUp.ReferenceOTU5944 |  |  |
| Armatimonadetes-Candidate class division FPB |  | 866043 |  |  |
| Armatimonadetes-Candidate class division FPB |  | New.CleanUp.ReferenceOTU3853 |  |  |
| Armatimonadetes-Candidate class division FPB |  | 934094 |  |  |
| Armatimonadetes-Candidate class division FPB |  | New.CleanUp.ReferenceOTU12573 |  |  |
| Armatimonadetes-Candidate class division FPB |  | New.CleanUp.ReferenceOTU14484 |  |  |
| Armatimonadetes-Candidate class division FPB |  | 1067515 |  |  |
| Armatimonadetes-Candidate class division FPB |  | New.CleanUp.ReferenceOTU13707 |  |  |
| Armatimonadetes-Candidate class division FPB |  | New.CleanUp.ReferenceOTU17107 |  |  |
| Armatimonadetes-Candidate class division FPB |  | 4359064 |  |  |
| Armatimonadetes-Candidate class division FPB |  | 979102 |  |  |
| Armatimonadetes-Candidate class division FPB |  | 3489297 |  |  |
| Armatimonadetes-Candidate class division FPB |  | New.CleanUp.ReferenceOTU23794 |  |  |
| Armatimonadetes-Candidate class division FPB |  | New.CleanUp.ReferenceOTU6076 |  |  |
| Armatimonadetes-Candidate class division FPB |  | 1098195 |  |  |
| Armatimonadetes-Candidate class division FPB |  | 940662 |  |  |
| Armatimonadetes-Candidate class division FPB |  | New.CleanUp.ReferenceOTU22922 |  |  |
| Armatimonadetes-Candidate class division FPB |  | 4483288 |  |  |
| Armatimonadetes-Candidate class division FPB |  | 512884 |  |  |
| Armatimonadetes-Candidate class division FPB |  | 65686 |  |  |
| Armatimonadetes-Candidate class division FPB |  | New.CleanUp.ReferenceOTU20799 |  |  |
| Armatimonadetes-Candidate class division FPB |  | New.CleanUp.ReferenceOTU4261 |  |  |
| Armatimonadetes-Candidate class division FPB |  | 1061713 |  |  |
| Bacteroidetes | *Flavia esturariibacter* | 894024 | Non-motile/Form multicellular filaments/Isolated from an estuary | [23] |
| Bacteroidetes | Sister clade to *Flavia esturariibacter* | New.CleanUp.ReferenceOTU19104 |  |  |
| Bacteroidetes | In the Chitinophagaceae (*Cnuella, Chitinophaga, Flavihumibacter*) | New.CleanUp.ReferenceOTU3022 | Aerobes or facultative aerobes/Hydrolysis of cellulose is known in some species | [24] |
| Bacteroidetes | In the Chitinophagaceae (*Cnuella, Chitinophaga, Flavihumibacter*) | 725882 |  |  |
| Bacteroidetes | In the Chitinophagaceae (*Cnuella, Chitinophaga, Flavihumibacter*) | 1002658 |  |  |
| Bacteroidetes | In the Chitinophagaceae (*Cnuella, Chitinophaga, Flavihumibacter*) | New.CleanUp.ReferenceOTU4576 |  |  |
| Bacteroidetes | In the Chitinophagaceae (*Flavitalea*) | 4335431 |  |  |
| Bacteroidetes | In the Chitinophagaceae (*Flavitalea*) | 255448 |  |  |
| Bacteroidetes | *Flavisolibacter* | New.CleanUp.ReferenceOTU236 |  |  |
| Bacteroidetes | *Flavisolibacter* | New.CleanUp.ReferenceOTU1178 |  |  |
| Bacteroidetes | In the Chitinophagaceae (*Flavisolibacter*) | 4417921 |  |  |
| Bacteroidetes | In the Chitinophagaceae (*Flavisolibacter*) | 243118 |  |  |
| Bacteroidetes | In the Chitinophagaceae (*Flavisolibacter*) | New.CleanUp.ReferenceOTU24933 |  |  |
| Bacteroidetes | In the Chitinophagaceae (*Flavisolibacter*) | 311656 |  |  |
| Bacteroidetes | In the Chitinophagaceae (*Flavisolibacter*) | 141786 |  |  |
| Bacteroidetes | In the Chitinophagaceae (*Flavisolibacter*) | New.CleanUp.ReferenceOTU1999 |  |  |
| Bacteroidetes | In the Chitinophagaceae (*Flavisolibacter*) | New.CleanUp.ReferenceOTU10349 |  |  |
| Bacteroidetes | In the Chitinophagaceae (*Flavisolibacter*) | New.CleanUp.ReferenceOTU16973 |  |  |
| Bacteroidetes | In the Chitinophagaceae (*Flavisolibacter*) | 2525736 |  |  |
| Bacteroidetes | In the Chitinophagaceae (*Flavisolibacter*) | 4418133 |  |  |
| Bacteroidetes | In the Chitinophagaceae (*Flavisolibacter*) | 532743 |  |  |
| Bacteroidetes | In the Chitinophagaceae (*Flavisolibacter*) | 594040 |  |  |
| Bacteroidetes | In the Chitinophagaceae (*Flavisolibacter*) | 1071316 |  |  |
| Bacteroidetes | In the Chitinophagaceae (*Flavisolibacter*) | New.CleanUp.ReferenceOTU15377 |  |  |
| Bacteroidetes | In the Chitinophagaceae (*Flavisolibacter*) | 945733 |  |  |
| Bacteroidetes | In the Chitinophagaceae (*Flavisolibacter*) | 545436 |  |  |
| Bacteroidetes | In the Chitinophagaceae (*Flavisolibacter*) | 4411669 |  |  |
| Bacteroidetes | In the Chitinophagaceae (*Flavisolibacter*) | 1037111 |  |  |
| Bacteroidetes | In the Chitinophagaceae (*Flavisolibacter*) | 997544 |  |  |
| Bacteroidetes | In the Chitinophagaceae (*Flavisolibacter*) | New.CleanUp.ReferenceOTU21126 |  |  |
| Bacteroidetes | In the Chitinophagaceae (*Flavisolibacter*) | 1084705 |  |  |
| Bacteroidetes | In the Chitinophagaceae (*Flavisolibacter*) | 813272 |  |  |
| Bacteroidetes | In the Chitinophagaceae (*Segetibacter*) | New.CleanUp.ReferenceOTU1852 |  |  |
| Bacteroidetes | In the Chitinophagaceae (*Segetibacter*) | New.CleanUp.ReferenceOTU6786 |  |  |
| Bacteroidetes | In the Chitinophagaceae (*Segetibacter*) | 570693 |  |  |
| Bacteroidetes | In the Chitinophagaceae (*Segetibacter*) | New.CleanUp.ReferenceOTU10051 |  |  |
| Bacteroidetes | In the Chitinophagaceae (*Segetibacter*) | New.CleanUp.ReferenceOTU12383 |  |  |
| Bacteroidetes | In the Chitinophagaceae (*Segetibacter*) | 324629 |  |  |
| Bacteroidetes | In the Chitinophagaceae (*Segetibacter*) | New.CleanUp.ReferenceOTU10088 |  |  |
| Bacteroidetes | In the Chitinophagaceae (*Segetibacter*) | 702181 |  |  |
| Bacteroidetes | In the Chitinophagaceae | New.CleanUp.ReferenceOTU19093 |  |  |
| Bacteroidetes | In the Chitinophagaceae | New.ReferenceOTU34 |  |  |
| Bacteroidetes | In the Chitinophagaceae | 1038987 |  |  |
| Bacteroidetes | In the Chitinophagaceae | 4323607 |  |  |
| Bacteroidetes | In the Chitinophagaceae | New.CleanUp.ReferenceOTU24747 |  |  |
| Bacteroidetes | In the Chitinophagaceae | 4301516 |  |  |
| Bacteroidetes | In the Chitinophagaceae | 620656 |  |  |
| Bacteroidetes | In the Chitinophagaceae | New.CleanUp.ReferenceOTU11923 |  |  |
| Bacteroidetes | In the Chitinophagaceae | 4297733 |  |  |
| Bacteroidetes | In the Chitinophagaceae | New.CleanUp.ReferenceOTU8880 |  |  |
| Bacteroidetes | In the Chitinophagaceae | 1102554 |  |  |
| Bacteroidetes | In the Chitinophagaceae | 1104847 |  |  |
| Bacteroidetes | In the Chitinophagaceae | 4333673 |  |  |
| Bacteroidetes | In the Chitinophagaceae | 513398 |  |  |
| Bacteroidetes | In the Chitinophagaceae | 803240 |  |  |
| Bacteroidetes | In the Chitinophagaceae | 1091321 |  |  |
| Bacteroidetes | In the Chitinophagaceae | 1012195 |  |  |
| Bacteroidetes | In the Chitinophagaceae | 1110139 |  |  |
| Bacteroidetes | In the Chitinophagaceae | 1020262 |  |  |
| Bacteroidetes | In the Chitinophagaceae | 4298761 |  |  |
| Bacteroidetes | In the Chitinophagaceae | 1141864 |  |  |
| Bacteroidetes | In the Chitinophagaceae | New.CleanUp.ReferenceOTU4239 |  |  |
| Bacteroidetes | In the Chitinophagaceae | 4436960 |  |  |
| Bacteroidetes | In the Chitinophagaceae | 32581 |  |  |
| Bacteroidetes | In the Chitinophagaceae | New.CleanUp.ReferenceOTU22650 |  |  |
| Bacteroidetes | In the Chitinophagaceae | 4044060 |  |  |
| Bacteroidetes | In the Chitinophagaceae | New.CleanUp.ReferenceOTU9971 |  |  |
| Bacteroidetes | In the Chitinophagaceae | 912669 |  |  |
| Bacteroidetes | In the Chitinophagaceae | 824675 |  |  |
| Bacteroidetes | In the Chitinophagaceae | 933150 |  |  |
| Bacteroidetes | In the Chitinophagaceae | 220305 |  |  |
| Bacteroidetes | In the Chitinophagaceae | New.CleanUp.ReferenceOTU16006 |  |  |
| Bacteroidetes | In the Chitinophagaceae | New.CleanUp.ReferenceOTU23695 |  |  |
| Bacteroidetes | In the Chitinophagaceae | New.CleanUp.ReferenceOTU331 |  |  |
| Bacteroidetes | In the Chitinophagaceae | New.CleanUp.ReferenceOTU14542 |  |  |
| Bacteroidetes | In the Chitinophagaceae | 958571 |  |  |
| Bacteroidetes | In the Chitinophagaceae | New.CleanUp.ReferenceOTU5485 |  |  |
| Bacteroidetes | In the Chitinophagaceae | 1118654 |  |  |
| Bacteroidetes | In the Chitinophagaceae | New.CleanUp.ReferenceOTU15598 |  |  |
| Bacteroidetes | In the Chitinophagaceae | 1052435 |  |  |
| Bacteroidetes | In the Chitinophagaceae | New.ReferenceOTU24 |  |  |
| Bacteroidetes | In the Chitinophagaceae | New.CleanUp.ReferenceOTU1681 |  |  |
| Bacteroidetes | In the Chitinophagaceae | 3549384 |  |  |
| Bacteroidetes | In the Chitinophagaceae | 4323887 |  |  |
| Bacteroidetes | In the Chitinophagaceae | New.CleanUp.ReferenceOTU14507 |  |  |
| Bacteroidetes | In the Chitinophagaceae | New.ReferenceOTU91 |  |  |
| Bacteroidetes | In the Chitinophagaceae | 4301518 |  |  |
| Bacteroidetes | *Rufibacter* | 267557 |  |  |
| Bacteroidetes | *Rufibacter* | New.CleanUp.ReferenceOTU1645 |  |  |
| Bacteroidetes | *Rufibacter* | 1068698 |  |  |
| Bacteroidetes | *Rufibacter* | 4397932 |  |  |
| Bacteroidetes | *Flexibacter flexilis* | 4370712 |  |  |
| Bacteroidetes | sister clade to Ohtaekwangia kribbensis | 1135504 |  |  |
| Bacteroidetes | sister clade to Ohtaekwangia kribbensis | 4318357 |  |  |
| Bacteroidetes | sister clade to Ohtaekwangia kribbensis | 3511168 |  |  |
| Bacteroidetes | sister clade to Ohtaekwangia kribbensis | New.CleanUp.ReferenceOTU9084 |  |  |
| Bacteroidetes | *In the Cytophagaceae (Pontibacter)* | 154032 |  |  |
| Bacteroidetes | *In the Cytophagaceae (Pontibacter)* | 4364575 |  |  |
| Bacteroidetes | *In the Cytophagaceae (Adhaeribacter)* | 356181 |  |  |
| Bacteroidetes | *In the Cytophagaceae (Adhaeribacter)* | 4424717 |  |  |
| Bacteroidetes | *In the Cytophagaceae (Adhaeribacter)* | 816789 |  |  |
| Bacteroidetes | *In the Cytophagaceae (Adhaeribacter)* | 3230031 |  |  |
| Bacteroidetes | *In the Cytophagaceae (Adhaeribacter)* | 1113105 |  |  |
| Bacteroidetes | *In the Cytophagaceae (Adhaeribacter)* | 764312 |  |  |
| Bacteroidetes | *In the Cytophagaceae (Adhaeribacter)* | New.CleanUp.ReferenceOTU2522 |  |  |
| Bacteroidetes | *In the Cytophagaceae (Adhaeribacter)* | New.CleanUp.ReferenceOTU7834 |  |  |
| Bacteroidetes | *In the Cytophagaceae (Adhaeribacter)* | New.CleanUp.ReferenceOTU5735 |  |  |
| Bacteroidetes | In the Cytophagaceae (*Rhodocytophaga*) | New.CleanUp.ReferenceOTU6368 |  |  |
| Bacteroidetes | In the Cytophagaceae (*Rhodocytophaga*) | New.CleanUp.ReferenceOTU15399 |  |  |
| Bacteroidetes | In the Cytophagaceae (*Rhodocytophaga*) | 811954 |  |  |
| Bacteroidetes | In the Cytophagaceae (*Rhodocytophaga*) | New.ReferenceOTU68 |  |  |
| Bacteroidetes | In the Cytophagaceae (*Rhodocytophaga*) | 3040675 |  |  |
| Bacteroidetes | In the Cytophagaceae (*Rhodocytophaga*) | New.CleanUp.ReferenceOTU15483 |  |  |
| Bacteroidetes | In the Cytophagaceae (*Rhodocytophaga*) | 811673 |  |  |
| Bacteroidetes | In the Cytophagaceae (*Rhodocytophaga*) | 770226 |  |  |
| Bacteroidetes | In the Cytophagaceae (*Rhodocytophaga*) | New.CleanUp.ReferenceOTU13678 |  |  |
| Bacteroidetes | In the Cytophagaceae (*Rhodocytophaga*) | New.CleanUp.ReferenceOTU16410 |  |  |
| Bacteroidetes | In the Cytophagaceae (*Rhodocytophaga*) | New.CleanUp.ReferenceOTU22908 |  |  |
| Bacteroidetes | In the Cytophagaceae (*Rhodocytophaga*) | New.ReferenceOTU3 |  |  |
| Bacteroidetes | In the Cytophagaceae (*Rhodocytophaga*) | New.CleanUp.ReferenceOTU13091 |  |  |
| Bacteroidetes | In the Cytophagaceae (*Rhodocytophaga*) | New.CleanUp.ReferenceOTU23363 |  |  |
| Bacteroidetes | In the Cytophagaceae (*Rhodocytophaga*) | 1124709 |  |  |
| Bacteroidetes | In the Cytophagaceae (*Rhodocytophaga*) | New.CleanUp.ReferenceOTU3757 |  |  |
| Bacteroidetes | In the Cytophagaceae (*Rhodocytophaga*) | New.CleanUp.ReferenceOTU7142 |  |  |
| Bacteroidetes | In the Cytophagaceae (*Rhodocytophaga*) | New.CleanUp.ReferenceOTU6972 |  |  |
| Bacteroidetes | In the Cytophagaceae (*Rhodocytophaga*) | New.CleanUp.ReferenceOTU24893 |  |  |
| Bacteroidetes | In the Cytophagaceae (*Rhodocytophaga*) | 277776 |  |  |
| Bacteroidetes | In the Cytophagaceae (*Rhodocytophaga*) | 4326799 |  |  |
| Bacteroidetes | In the Cytophagaceae (*Rhodocytophaga*) | New.CleanUp.ReferenceOTU14510 |  |  |
| Bacteroidetes | In the Cytophagaceae (*Rhodocytophaga*) | 4379834 |  |  |
| Bacteroidetes | In the Cytophagaceae (*Rhodocytophaga*) | 1106318 |  |  |
| Bacteroidetes | In the Cytophagaceae (*Rhodocytophaga*) | New.ReferenceOTU50 |  |  |
| Bacteroidetes | In the Cytophagaceae (*Cytophaga*) | 4457944 |  |  |
| Bacteroidetes | In the Cytophagaceae | 1131830 |  |  |
| Bacteroidetes | In the Cytophagaceae | 1148341 |  |  |
| Bacteroidetes | In the Cytophagaceae | New.CleanUp.ReferenceOTU5360 |  |  |
| Bacteroidetes | In the Cytophagaceae | New.ReferenceOTU65 |  |  |
| Bacteroidetes | In the Cytophagaceae | New.CleanUp.ReferenceOTU8571 |  |  |
| Bacteroidetes | In the Cytophagaceae | New.CleanUp.ReferenceOTU23386 |  |  |
| Bacteroidetes | In the Cytophagaceae | New.CleanUp.ReferenceOTU14110 |  |  |
| Bacteroidetes | In the Cytophagaceae | 821788 |  |  |
| Bacteroidetes | In the Cytophagaceae | 317511 |  |  |
| Bacteroidetes | In the Cytophagaceae | New.CleanUp.ReferenceOTU23235 |  |  |
| Bacteroidetes | In the Cytophagaceae | 138309 |  |  |
| Bacteroidetes | In the Cytophagaceae | New.CleanUp.ReferenceOTU7889 |  |  |
| Bacteroidetes | In the Cytophagaceae | New.CleanUp.ReferenceOTU6899 |  |  |
| Bacteroidetes | In the Cytophagaceae | New.ReferenceOTU54 |  |  |
| Bacteroidetes | In the Cytophagaceae | 947849 |  |  |
| Bacteroidetes | In the Cytophagaceae | 697457 |  |  |
| Bacteroidetes | In the Cytophagaceae | New.CleanUp.ReferenceOTU23781 |  |  |
| Bacteroidetes | In the Cytophagaceae | New.CleanUp.ReferenceOTU10318 |  |  |
| Bacteroidetes | In the Cytophagaceae | New.CleanUp.ReferenceOTU19865 |  |  |
| Bacteroidetes | In the Cytophagaceae | New.CleanUp.ReferenceOTU581 |  |  |
| Bacteroidetes | In the Cytophagaceae | New.CleanUp.ReferenceOTU16741 |  |  |
| Bacteroidetes | In the Cytophagaceae | New.CleanUp.ReferenceOTU14621 |  |  |
| Bacteroidetes | In the Cytophagaceae | New.ReferenceOTU51 |  |  |
| Bacteroidetes | In the Cytophagaceae | New.CleanUp.ReferenceOTU337 |  |  |
| Bacteroidetes | In the Cytophagaceae | New.ReferenceOTU55 |  |  |
| Bacteroidetes | In the Cytophagaceae | 367415 |  |  |
| Bacteroidetes | In the Cytophagaceae | New.CleanUp.ReferenceOTU4268 |  |  |
| Bacteroidetes | In the Cytophagaceae | 357873 |  |  |
| Bacteroidetes | In the Cytophagaceae | New.CleanUp.ReferenceOTU5617 |  |  |
| Bacteroidetes | In the Cytophagaceae | 725240 |  |  |
| Bacteroidetes | In the Cytophagaceae | New.CleanUp.ReferenceOTU1773 |  |  |
| Bacteroidetes | In the Cytophagaceae | New.CleanUp.ReferenceOTU21344 |  |  |
| Bacteroidetes | In the Cytophagaceae | New.CleanUp.ReferenceOTU7241 |  |  |
| Bacteroidetes | In the Cytophagaceae | New.CleanUp.ReferenceOTU8828 |  |  |
| Bacteroidetes | In the Cytophagaceae | New.CleanUp.ReferenceOTU16481 |  |  |
| Bacteroidetes | In the Cytophagaceae | New.CleanUp.ReferenceOTU3410 |  |  |
| Bacteroidetes | In the Cytophagaceae | New.CleanUp.ReferenceOTU8918 |  |  |
| Bacteroidetes | In the Cytophagaceae | 1111968 |  |  |
| Bacteroidetes | In the Cytophagaceae | New.CleanUp.ReferenceOTU21370 |  |  |
| Bacteroidetes | In the Cytophagaceae | New.CleanUp.ReferenceOTU3951 |  |  |
| Bacteroidetes | In the Cytophagaceae | New.CleanUp.ReferenceOTU5449 |  |  |
| Bacteroidetes | In the Cytophagaceae | New.CleanUp.ReferenceOTU6082 |  |  |
| Bacteroidetes | In the Cytophagaceae | New.CleanUp.ReferenceOTU10775 |  |  |
| Bacteroidetes | In the Cytophagaceae | New.CleanUp.ReferenceOTU23785 |  |  |
| Bacteroidetes | In the Cytophagaceae | New.CleanUp.ReferenceOTU1376 |  |  |
| Bacteroidetes | In the Cytophagaceae | 175203 |  |  |
| Bacteroidetes | In the Cytophagaceae | New.CleanUp.ReferenceOTU12840 |  |  |
| Bacteroidetes | In the Cytophagaceae | New.CleanUp.ReferenceOTU5299 |  |  |
| Bacteroidetes | In the Cytophagaceae | 1103871 |  |  |
| Bacteroidetes | In the Cytophagaceae | 1138934 |  |  |
| Bacteroidetes | In the Cytophagaceae | 4256699 |  |  |
| Bacteroidetes | In the Cytophagaceae | 4480958 |  |  |
| Bacteroidetes | In the Cytophagaceae | 985339 |  |  |
| Bacteroidetes | In the Cytophagaceae | 249391 |  |  |
| Bacteroidetes | *Adhaeribacter* | 1069076 | Heterotrophs, aerobes or facultative aerobes non-spore forming rods/Cytophaga-like bacteria are known to lyse cyanobacteria/Found in marine actinians, seawater, desert soils, dust particles, and forest soil | [25,26] |
| Bacteroidetes | *Hymenobacter* | 824700 | Non-motile, Gram-negative/Isolated from sandstone, soil, irradiated pork, uranium mine wastes, freshwater, and air | [26] |
| Bacteroidetes | *Hymenobacter* | New.CleanUp.ReferenceOTU20920 |  |  |
| Bacteroidetes | *Hymenobacter* | New.CleanUp.ReferenceOTU14811 |  |  |
| Bacteroidetes | *Hymenobacter* | 687649 |  |  |
| Bacteroidetes | *Hymenobacter* | 541746 |  |  |
| Bacteroidetes | *Hymenobacter* | 1090273 |  |  |
| Bacteroidetes | *Hymenobacter* | 150955 |  |  |
| Bacteroidetes | *Hymenobacter* | 789806 |  |  |
| Bacteroidetes | *Hymenobacter* | 4404498 |  |  |
| Bacteroidetes | *Hymenobacter* | 2621271 |  |  |
| Bacteroidetes | *Hymenobacter* | 1981833 |  |  |
| Bacteroidetes | *Hymenobacter* | 1090978 |  |  |
| Bacteroidetes | *Hymenobacter* | 927623 |  |  |
| Bacteroidetes | *Hymenobacter* | New.ReferenceOTU105 |  |  |
| Bacteroidetes | *Hymenobacter* | 3406670 |  |  |
| Bacteroidetes | In the Flammeovirgaceae | New.CleanUp.ReferenceOTU20855 | Gram-negative/Found in soils and marine environments | [27] |
| Bacteroidetes | In the Flammeovirgaceae | 1105341 |  |  |
| Bacteroidetes | *Flavobacterium* | New.CleanUp.ReferenceOTU24487 | Chemoorganotrophs/Found in freshwater and in soil | [28] |
| Bacteroidetes | *Flavobacterium* | 1055322 |  |  |
| Bacteroidetes | In the Sphingobacteriales | New.CleanUp.ReferenceOTU11436 |  |  |
| Bacteroidetes | In the Sphingobacteriales | New.CleanUp.ReferenceOTU22392 | Gram-negative, non-spore forming bacilli/Isolated from soils and compost | [29] |
| Bacteroidetes | In the Sphingobacteriaceae | New.CleanUp.ReferenceOTU14970 |  |  |
| Bacteroidetes | In the Sphingobacteriaceae | New.CleanUp.ReferenceOTU14605 |  |  |
| Bacteroidetes | In the Sphingobacteriaceae | New.CleanUp.ReferenceOTU24872 |  |  |
| Bacteroidetes | In the Sphingobacteriaceae | New.CleanUp.ReferenceOTU2937 |  |  |
| Bacteroidetes | In the Sphingobacteriaceae | 586829 |  |  |
| Bacteroidetes | *Pedobacter* | New.CleanUp.ReferenceOTU2182 |  |  |
| Bacteroidetes | *Pedobacter* | New.CleanUp.ReferenceOTU23158 |  |  |
| Bacteroidetes | *Pedobacter* | 1088120 |  |  |
| Bacteroidetes | *Pedobacter* | 1098634 |  |  |
| Bacteroidetes | *Pedobacter* | 575305 |  |  |
| Bacteroidetes | *Pedobacter* | 718367 |  |  |
| Bacteroidetes | *Pedobacter* | 810109 |  |  |
| Bacteroidetes | *Pedobacter* | 635391 |  |  |
| Bacteroidetes | *Pedobacter* | New.CleanUp.ReferenceOTU24930 |  |  |
| Bacteroidetes | *Pedobacter* | 987696 |  |  |
| Bacteroidetes | Unassigned | New.CleanUp.ReferenceOTU8662 |  |  |
| Bacteroidetes | Unassigned | 235423 |  |  |
| Bacteroidetes | Unassigned | 4366956 |  |  |
| Bacteroidetes | Unassigned | New.CleanUp.ReferenceOTU7193 |  |  |
| Bacteroidetes | Unassigned | 4342317 |  |  |
| Bacteroidetes | Unassigned | New.CleanUp.ReferenceOTU21070 |  |  |
| Bacteroidetes | Unassigned | New.CleanUp.ReferenceOTU21661 |  |  |
| Bacteroidetes | Unassigned | 4471717 |  |  |
| Bacteroidetes | Unassigned | New.CleanUp.ReferenceOTU13943 |  |  |
| Bacteroidetes | Unassigned | New.CleanUp.ReferenceOTU9825 |  |  |
| BCR | Unassigned – closest culture *Aciditerrimonas ferrireducens* | New.CleanUp.ReferenceOTU6554 |  |  |
| BCR | Unassigned – closest culture *Aciditerrimonas ferrireducens* | New.CleanUp.ReferenceOTU12288 |  |  |
| Chlorobi | Sister clade with Ignavibacteriales | New.CleanUp.ReferenceOTU16337 | Facultative anaerobes/obligated heterotrophic bacteria/Found in terrestrial habitats | [30] |
| Chlorobi | Sister clade with Ignavibacteriales | New.CleanUp.ReferenceOTU11621 |  |  |
| Chlorobi | Sister clade with Ignavibacteriales | 107418 |  |  |
| Chloroflexi | Sister clade to Chloroflexaceae (*Chloroflexus auranticus/aggregans*) | 554361 | Anoxygenic phototrophic bacteria | [31] |
| Chloroflexi | In the Thermomicrobia | 1110592 | Thermophilic green non-sulfur bacteria |  |
| Chloroflexi | In the Thermomicrobia | 112867 |  |  |
| Chloroflexi | In the Thermomicrobia | 217746 |  |  |
| Chloroflexi | In the Thermomicrobia | New.CleanUp.ReferenceOTU3941 |  |  |
| Chloroflexi | In the Thermomicrobia | New.CleanUp.ReferenceOTU21477 |  |  |
| Chloroflexi | Sister clade to Thermomicrobia | New.ReferenceOTU100 |  |  |
| Chloroflexi | *Herpetosiphon* | New.CleanUp.ReferenceOTU10489 | Non-phototrophic bacteria/Isolated from slimy coated springs |  |
| Chloroflexi | In the Kallotenuaceae (*Kallotenue*) | New.CleanUp.ReferenceOTU21147 | Non-phototrophic bacteria/multicellular filaments |  |
| Chloroflexi | In the Kallotenuaceae (*Kallotenue*) | New.CleanUp.ReferenceOTU17768 |  |  |
| Chloroflexi | In the Caldilineaceae (*Caldilinea, Litorilinea*) | New.CleanUp.ReferenceOTU3507 |  |  |
| Chloroflexi | In the Caldilineaceae (*Caldilinea, Litorilinea*) | 3897233 |  |  |
| Chloroflexi | In the Caldilineaceae (*Caldilinea, Litorilinea*) | New.CleanUp.ReferenceOTU9864 |  |  |
| Chloroflexi | Unassigned Chloroflexi (*Kouleothrix*) | New.ReferenceOTU58 |  |  |
| Chloroflexi | Unassigned Chloroflexi (*Kouleothrix*) | New.CleanUp.ReferenceOTU21814 |  |  |
| Chloroflexi | Unassigned | New.CleanUp.ReferenceOTU4022 |  |  |
| Chloroflexi | Unassigned | 4328659 |  |  |
| Chloroflexi | Unassigned | New.CleanUp.ReferenceOTU18331 |  |  |
| Chloroflexi | Unassigned | New.CleanUp.ReferenceOTU7328 |  |  |
| Chloroflexi | Unassigned | New.CleanUp.ReferenceOTU2212 |  |  |
| Chloroflexi | Unassigned | New.CleanUp.ReferenceOTU23974 |  |  |
| Chloroflexi | Unassigned | New.CleanUp.ReferenceOTU24897 |  |  |
| Chloroflexi | Unassigned | New.CleanUp.ReferenceOTU14864 |  |  |
| Chloroflexi | Unassigned | New.CleanUp.ReferenceOTU17904 |  |  |
| Chloroflexi | Unassigned | New.CleanUp.ReferenceOTU20667 |  |  |
| Chloroflexi | Unassigned | New.CleanUp.ReferenceOTU6887 |  |  |
| Chloroflexi | Unassigned | 831877 |  |  |
| Chloroflexi | Unassigned | 185950 |  |  |
| Chloroflexi | Unassigned | New.CleanUp.ReferenceOTU2534 |  |  |
| Chloroflexi | Unassigned | New.CleanUp.ReferenceOTU2928 |  |  |
| Chloroflexi | Unassigned | 549954 |  |  |
| Chloroflexi | Unassigned | 247875 |  |  |
| Chloroflexi | Unassigned | 4482713 |  |  |
| Chloroflexi | Unassigned | New.CleanUp.ReferenceOTU2593 |  |  |
| Chloroflexi | Unassigned | New.CleanUp.ReferenceOTU11129 |  |  |
| Chloroflexi | Unassigned | 52036 |  |  |
| Chloroflexi | Unassigned | New.CleanUp.ReferenceOTU15163 |  |  |
| Chloroflexi | Unassigned | 1143895 |  |  |
| Chloroflexi | Unassigned | New.ReferenceOTU86 |  |  |
| Chloroflexi | Unassigned | New.CleanUp.ReferenceOTU16261 |  |  |
| Chloroflexi | Unassigned | New.CleanUp.ReferenceOTU18762 |  |  |
| Archaea | *Nitrososphaera* | 720511 | Autotrophic o mixotrophic, ammonia oxidizing organisms/Found in soils, marine environments, and hot springs | [32] |
| Archaea | *Nitrososphaera* | 748601 |  |  |
| Archaea | *Nitrososphaera* | 107234 |  |  |
| Cyanobacteria | *Oscillatoria* | New.CleanUp.ReferenceOTU21607 | Photoautotrophs/ubiquitous | [33] |
| Cyanobacteria | *Gleiterinema* | New.CleanUp.ReferenceOTU16228 |  |  |
| Cyanobacteria | *Lyngbya* | 819703 |  |  |
| Cyanobacteria | *Lyngbya* | 223377 |  |  |
| Cyanobacteria | *Lyngbya* | 278544 |  |  |
| Cyanobacteria | *Lyngbya* | New.CleanUp.ReferenceOTU15447 |  |  |
| Cyanobacteria | *Lyngbya* | New.CleanUp.ReferenceOTU2491 |  |  |
| Cyanobacteria | *Lyngbya* | 4342315 |  |  |
| Cyanobacteria | *Leptolyngybia* | 361839 |  |  |
| Cyanobacteria | *Leptolyngybia* | 575555 |  |  |
| Cyanobacteria | *Leptolyngybia* | New.CleanUp.ReferenceOTU12471 |  |  |
| Cyanobacteria | *Leptolyngybia* | New.CleanUp.ReferenceOTU14157 |  |  |
| Cyanobacteria | *Leptolyngybia* | 1552835 |  |  |
| Cyanobacteria | *Leptolyngybia* | 818188 |  |  |
| Cyanobacteria | *Leptolyngybia* | New.CleanUp.ReferenceOTU23046 |  |  |
| Cyanobacteria | *Leptolyngybia* | New.CleanUp.ReferenceOTU11872 |  |  |
| Cyanobacteria | *Leptolyngybia* | New.CleanUp.ReferenceOTU16449 |  |  |
| Cyanobacteria | *Leptolyngybia* | New.CleanUp.ReferenceOTU15934 |  |  |
| Cyanobacteria | *Leptolyngybia* | New.CleanUp.ReferenceOTU23168 |  |  |
| Cyanobacteria | *Leptolyngybia* | New.CleanUp.ReferenceOTU18462 |  |  |
| Cyanobacteria | *Leptolyngybia* | New.CleanUp.ReferenceOTU16266 |  |  |
| Cyanobacteria | *Leptolyngybia* | 153279 |  |  |
| Cyanobacteria | *Leptolyngybia* | 4432360 |  |  |
| Cyanobacteria | *Leptolyngybia* | 273195 |  |  |
| Cyanobacteria | *M. steenstrupii* | New.CleanUp.ReferenceOTU6677 |  |  |
| Cyanobacteria | *M. steenstrupii* | New.CleanUp.ReferenceOTU18702 |  |  |
| Cyanobacteria | *M. steenstrupii* | New.CleanUp.ReferenceOTU15091 |  |  |
| Cyanobacteria | *M. steenstrupii* | New.CleanUp.ReferenceOTU18757 |  |  |
| Cyanobacteria | *M. steenstrupii* | New.CleanUp.ReferenceOTU6947 |  |  |
| Cyanobacteria | *M. steenstrupii* | New.CleanUp.ReferenceOTU9250 |  |  |
| Cyanobacteria | *M. steenstrupii* | New.CleanUp.ReferenceOTU4651 |  |  |
| Cyanobacteria | *M. steenstrupii* | New.CleanUp.ReferenceOTU23807 |  |  |
| Cyanobacteria | *M. steenstrupii* | New.CleanUp.ReferenceOTU20390 |  |  |
| Cyanobacteria | *M. steenstrupii* | New.CleanUp.ReferenceOTU20883 |  |  |
| Cyanobacteria | *M. steenstrupii* | New.CleanUp.ReferenceOTU4505 |  |  |
| Cyanobacteria | *M. steenstrupii* | New.CleanUp.ReferenceOTU3491 |  |  |
| Cyanobacteria | *M. steenstrupii* | New.CleanUp.ReferenceOTU3019 |  |  |
| Cyanobacteria | *M. steenstrupii* | 278371 |  |  |
| Cyanobacteria | *M. steenstrupii* | New.CleanUp.ReferenceOTU1908 |  |  |
| Cyanobacteria | *M. steenstrupii* | New.CleanUp.ReferenceOTU18710 |  |  |
| Cyanobacteria | *M. steenstrupii* | New.CleanUp.ReferenceOTU10091 |  |  |
| Cyanobacteria | *M. steenstrupii* | 820606 |  |  |
| Cyanobacteria | *M. steenstrupii* | New.CleanUp.ReferenceOTU24007 |  |  |
| Cyanobacteria | *M. steenstrupii* | New.CleanUp.ReferenceOTU23087 |  |  |
| Cyanobacteria | *M. steenstrupii* | New.CleanUp.ReferenceOTU22975 |  |  |
| Cyanobacteria | *M. steenstrupii* | New.CleanUp.ReferenceOTU20444 |  |  |
| Cyanobacteria | *M. steenstrupii* | New.CleanUp.ReferenceOTU18539 |  |  |
| Cyanobacteria | *M. steenstrupii* | New.CleanUp.ReferenceOTU17896 |  |  |
| Cyanobacteria | *M. steenstrupii* | New.CleanUp.ReferenceOTU22902 |  |  |
| Cyanobacteria | *M. steenstrupii* | New.CleanUp.ReferenceOTU9950 |  |  |
| Cyanobacteria | *M. steenstrupii* | 649198 |  |  |
| Cyanobacteria | *M. steenstrupii* | New.CleanUp.ReferenceOTU14148 |  |  |
| Cyanobacteria | *M. steenstrupii* | 181039 |  |  |
| Cyanobacteria | *M. steenstrupii* | New.CleanUp.ReferenceOTU13162 |  |  |
| Cyanobacteria | *M. steenstrupii* | New.ReferenceOTU70 |  |  |
| Cyanobacteria | *M. steenstrupii* | New.ReferenceOTU81 |  |  |
| Cyanobacteria | *M. steenstrupii* | New.CleanUp.ReferenceOTU22202 |  |  |
| Cyanobacteria | *M. steenstrupii* | New.CleanUp.ReferenceOTU6847 |  |  |
| Cyanobacteria | *M. steenstrupii* | New.CleanUp.ReferenceOTU16628 |  |  |
| Cyanobacteria | *M. steenstrupii* | New.CleanUp.ReferenceOTU999 |  |  |
| Cyanobacteria | *M. steenstrupii* | New.CleanUp.ReferenceOTU4627 |  |  |
| Cyanobacteria | *M. steenstrupii* | New.CleanUp.ReferenceOTU22529 |  |  |
| Cyanobacteria | *M. steenstrupii* | New.CleanUp.ReferenceOTU21079 |  |  |
| Cyanobacteria | *M. steenstrupii* | New.CleanUp.ReferenceOTU21091 |  |  |
| Cyanobacteria | *M. steenstrupii* | New.CleanUp.ReferenceOTU20219 |  |  |
| Cyanobacteria | *M. steenstrupii* | New.CleanUp.ReferenceOTU1991 |  |  |
| Cyanobacteria | *M. chthonoplastes* | New.CleanUp.ReferenceOTU3218 |  |  |
| Cyanobacteria | In the Oscillatoriales (*Lyngbya/M. chthonoplastes)* | New.CleanUp.ReferenceOTU2541 |  |  |
| Cyanobacteria | In the Oscillatoriales (*Lyngbya/Oscillatoria*) | New.ReferenceOTU64 |  |  |
| Cyanobacteria | In the Oscillatoriales | 4322506 |  |  |
| Cyanobacteria | In the Oscillatoriales | New.CleanUp.ReferenceOTU17032 |  |  |
| Cyanobacteria | In the Oscillatoriales | New.CleanUp.ReferenceOTU7367 |  |  |
| Cyanobacteria | In the Oscillatoriales | New.CleanUp.ReferenceOTU15551 |  |  |
| Cyanobacteria | In the Oscillatoriales | New.CleanUp.ReferenceOTU19772 |  |  |
| Cyanobacteria | In the Oscillatoriales | New.CleanUp.ReferenceOTU14156 |  |  |
| Cyanobacteria | In the Oscillatoriales | New.CleanUp.ReferenceOTU12647 |  |  |
| Cyanobacteria | In the Oscillatoriales | New.CleanUp.ReferenceOTU10687 |  |  |
| Cyanobacteria | In the Oscillatoriales | 769222 |  |  |
| Cyanobacteria | In the Oscillatoriales | New.CleanUp.ReferenceOTU8630 |  |  |
| Cyanobacteria | In the Oscillatoriales | New.CleanUp.ReferenceOTU17523 |  |  |
| Cyanobacteria | In the Oscillatoriales | New.CleanUp.ReferenceOTU15920 |  |  |
| Cyanobacteria | In the Oscillatoriales | New.CleanUp.ReferenceOTU24665 |  |  |
| Cyanobacteria | In the Oscillatoriales | New.CleanUp.ReferenceOTU13676 |  |  |
| Cyanobacteria | In the Oscillatoriales | New.CleanUp.ReferenceOTU3319 |  |  |
| Cyanobacteria | In the Oscillatoriales | New.CleanUp.ReferenceOTU3322 |  |  |
| Cyanobacteria | In the Oscillatoriales | New.CleanUp.ReferenceOTU3813 |  |  |
| Cyanobacteria | In the Oscillatoriales | New.CleanUp.ReferenceOTU4436 |  |  |
| Cyanobacteria | In the Oscillatoriales | New.CleanUp.ReferenceOTU3834 |  |  |
| Cyanobacteria | In the Oscillatoriales | New.CleanUp.ReferenceOTU9960 |  |  |
| Cyanobacteria | In the Oscillatoriales | New.CleanUp.ReferenceOTU20819 |  |  |
| Cyanobacteria | In the Oscillatoriales | New.ReferenceOTU76 |  |  |
| Cyanobacteria | In the Oscillatoriales | New.CleanUp.ReferenceOTU17603 |  |  |
| Cyanobacteria | In the Oscillatoriales | New.CleanUp.ReferenceOTU20406 |  |  |
| Cyanobacteria | In the Oscillatoriales | New.CleanUp.ReferenceOTU2934 |  |  |
| Cyanobacteria | In the Oscillatoriales | New.CleanUp.ReferenceOTU11834 |  |  |
| Cyanobacteria | In the Oscillatoriales | New.CleanUp.ReferenceOTU12774 |  |  |
| Cyanobacteria | In the Oscillatoriales | New.CleanUp.ReferenceOTU14426 |  |  |
| Cyanobacteria | In the Oscillatoriales | New.CleanUp.ReferenceOTU1759 |  |  |
| Cyanobacteria | In the Oscillatoriales | New.CleanUp.ReferenceOTU20821 |  |  |
| Cyanobacteria | In the Oscillatoriales | New.CleanUp.ReferenceOTU19525 |  |  |
| Cyanobacteria | In the Oscillatoriales | 3544 |  |  |
| Cyanobacteria | In the Oscillatoriales | New.ReferenceOTU17 |  |  |
| Cyanobacteria | In the Oscillatoriales | New.CleanUp.ReferenceOTU16459 |  |  |
| Cyanobacteria | In the Oscillatoriales | 4466028 |  |  |
| Cyanobacteria | *Aphanocapsa* | 702683 |  |  |
| Cyanobacteria | *Aphanocapsa* | New.CleanUp.ReferenceOTU23642 |  |  |
| Cyanobacteria | *Chlorogloea* | New.CleanUp.ReferenceOTU5391 |  |  |
| Cyanobacteria | *Chlorogloea* | 203466 |  |  |
| Cyanobacteria | *Chlorogloea* | New.CleanUp.ReferenceOTU19232 |  |  |
| Cyanobacteria | *Chroococcidiopsis* | 4466932 |  |  |
| Cyanobacteria | *Chroococcidiopsis* | 649507 |  |  |
| Cyanobacteria | *Chroococcidiopsis* | New.CleanUp.ReferenceOTU19838 |  |  |
| Cyanobacteria | *Chroococcidiopsis* | New.ReferenceOTU75 |  |  |
| Cyanobacteria | *Chroococcidiopsis* | New.CleanUp.ReferenceOTU6003 |  |  |
| Cyanobacteria | *Chroococcidiopsis* | New.CleanUp.ReferenceOTU19966 |  |  |
| Cyanobacteria | *Chroococcidiopsis* | 505954 |  |  |
| Cyanobacteria | *Chroococcidiopsis* | 396285 |  |  |
| Cyanobacteria | *Chroococcidiopsis* | 71326 |  |  |
| Cyanobacteria | *Chroococcidiopsis* | 224486 |  |  |
| Cyanobacteria | *Chroococcidiopsis* | New.CleanUp.ReferenceOTU24557 |  |  |
| Cyanobacteria | *Chroococcidiopsis* | New.CleanUp.ReferenceOTU24021 |  |  |
| Cyanobacteria | *Chroococcidiopsis* | New.ReferenceOTU74 |  |  |
| Cyanobacteria | *Chroococcidiopsis* | 810188 |  |  |
| Cyanobacteria | *Chroococcidiopsis* | 818439 |  |  |
| Cyanobacteria | *Chroococcidiopsis* | 813107 |  |  |
| Cyanobacteria | *Chroococcidiopsis* | New.CleanUp.ReferenceOTU13892 |  |  |
| Cyanobacteria | In the Chroococcales | New.CleanUp.ReferenceOTU10402 |  |  |
| Cyanobacteria | In the Chroococcales | New.CleanUp.ReferenceOTU20772 |  |  |
| Cyanobacteria | In the Chroococcales | New.ReferenceOTU18 |  |  |
| Cyanobacteria | In the Chroococcales | 808252 |  |  |
| Cyanobacteria | In the Chroococcales | New.CleanUp.ReferenceOTU6679 |  |  |
| Cyanobacteria | In the Chroococcales | New.CleanUp.ReferenceOTU14429 |  |  |
| Cyanobacteria | *Loriellopsis* | 129048 | Photoautotrophs/Nitrogen fixers/ubiquitous | [33,34] |
| Cyanobacteria | *Loriellopsis* | 35330 |  |  |
| Cyanobacteria | *Loriellopsis* | 2307137 |  |  |
| Cyanobacteria | *Nostoc* | 221674 |  |  |
| Cyanobacteria | *Nostoc* | 312035 |  |  |
| Cyanobacteria | *Nostoc* | 99364 |  |  |
| Cyanobacteria | *Tolypothrix* | 198952 |  |  |
| Cyanobacteria | *Tolypothrix* | New.CleanUp.ReferenceOTU16262 |  |  |
| Cyanobacteria | *Tolypothrix* | 178178 |  |  |
| Cyanobacteria | *Tolypothrix* | New.CleanUp.ReferenceOTU10337 |  |  |
| Cyanobacteria | *Tolypothrix* | New.CleanUp.ReferenceOTU12634 |  |  |
| Cyanobacteria | *Tolypothrix* | 106317 |  |  |
| Cyanobacteria | *Tolypothrix* | 221130 |  |  |
| Cyanobacteria | *Tolypothrix* | New.CleanUp.ReferenceOTU18974 |  |  |
| Cyanobacteria | *Tolypothrix* | New.CleanUp.ReferenceOTU20972 |  |  |
| Cyanobacteria | *Tolypothrix* | 813352 |  |  |
| Cyanobacteria | *Scytonema* | 716611 |  |  |
| Cyanobacteria | *Scytonema* | New.CleanUp.ReferenceOTU5012 |  |  |
| Cyanobacteria | *Scytonema* | 277671 |  |  |
| Cyanobacteria | *Fischerella* | New.CleanUp.ReferenceOTU20746 |  |  |
| Cyanobacteria | *Fischerella* | New.ReferenceOTU85 |  |  |
| Cyanobacteria | In the Nostocales | New.CleanUp.ReferenceOTU17179 |  |  |
| Cyanobacteria | In the Nostocales | New.CleanUp.ReferenceOTU19428 |  |  |
| Cyanobacteria | In the Nostocales | New.CleanUp.ReferenceOTU11205 |  |  |
| Cyanobacteria | In the Nostocales | New.CleanUp.ReferenceOTU9276 |  |  |
| Cyanobacteria | Unassigned | New.CleanUp.ReferenceOTU9410 | Photoautotrophs/ubiquitous | [33] |
| Cyanobacteria | Unassigned | New.CleanUp.ReferenceOTU9898 |  |  |
| Cyanobacteria | Unassigned | New.CleanUp.ReferenceOTU22839 |  |  |
| Cyanobacteria | Unassigned | New.CleanUp.ReferenceOTU20384 |  |  |
| Cyanobacteria | Unassigned | New.CleanUp.ReferenceOTU12747 |  |  |
| Cyanobacteria | Unassigned | New.CleanUp.ReferenceOTU21785 |  |  |
| Cyanobacteria | Unassigned | New.CleanUp.ReferenceOTU83 |  |  |
| Cyanobacteria | Unassigned | New.CleanUp.ReferenceOTU13613 |  |  |
| Cyanobacteria | Unassigned | 327421 |  |  |
| Cyanobacteria | Unassigned | New.CleanUp.ReferenceOTU4762 |  |  |
| Cyanobacteria | Unassigned | New.CleanUp.ReferenceOTU10299 |  |  |
| Cyanobacteria | Unassigned | New.CleanUp.ReferenceOTU16982 |  |  |
| Cyanobacteria | Unassigned | 808657 |  |  |
| Cyanobacteria | Unassigned | New.CleanUp.ReferenceOTU24793 |  |  |
| Cyanobacteria | Unassigned | New.CleanUp.ReferenceOTU15657 |  |  |
| Cyanobacteria | Unassigned | New.CleanUp.ReferenceOTU5708 |  |  |
| Cyanobacteria | Unassigned | New.CleanUp.ReferenceOTU9358 |  |  |
| Firmicutes | *Bacillus* | 319982 | Endospore forming bacteria/Resistant to desiccation/can survive extreme conditions/Found in terrestrial and aquatic environments | [35] |
| Firmicutes | *Bacillus* | New.ReferenceOTU63 |  |  |
| Firmicutes | In the Bacillaceae (*Bacillus*) | 1078248 |  |  |
| Firmicutes | In the Bacillaceae (*Bacillus*) | 823024 |  |  |
| Firmicutes | In the Bacillaceae (*Bacillus*) | New.CleanUp.ReferenceOTU1624 |  |  |
| Firmicutes | In the Bacillaceae (*Bacillus*) | 827089 |  |  |
| Firmicutes | In the Bacillaceae (*Bacillus*) | 954381 |  |  |
| Firmicutes | In the Bacillaceae (*Bacillus*) | 854050 |  |  |
| Firmicutes | In the Bacilli (*Bacillus, Sporosarcina*) | 1051517 |  |  |
| Firmicutes | In the Bacilli (*Bacillus, Sporosarcina*) | 833645 |  |  |
| Firmicutes | In the Bacilli (*Bacillus, Sporosarcina*) | 833317 |  |  |
| Firmicutes | In the Bacilli (*Planococcus, Planomicrobium*) | 540737 |  |  |
| Firmicutes | *Brevibacillus* | 640652 |  |  |
| Firmicutes | *Brevibacillus* | New.CleanUp.ReferenceOTU4908 |  |  |
| Firmicutes | *Brevibacillus* | New.CleanUp.ReferenceOTU17284 |  |  |
| Firmicutes | *Brevibacillus* | 95847 |  |  |
| Firmicutes | *Brevibacillus* | New.CleanUp.ReferenceOTU9143 |  |  |
| Firmicutes | *Brevibacillus* | 307934 |  |  |
| Firmicutes | *Paenibacillus* | 240501 |  |  |
| Firmicutes | *Paenibacillus* | 809744 |  |  |
| Firmicutes | *Paenibacillus* | 4339146 |  |  |
| Firmicutes | *Paenibacillus* | 4310348 |  |  |
| Firmicutes | *Paenibacillus* | New.CleanUp.ReferenceOTU13214 |  |  |
| Firmicutes | *Paenibacillus* | 589407 |  |  |
| Firmicutes | *Paenibacillus* | 553697 |  |  |
| Firmicutes | *Paenibacillus* | New.CleanUp.ReferenceOTU23826 |  |  |
| Firmicutes | *Paenibacillus* | New.CleanUp.ReferenceOTU467 |  |  |
| Firmicutes | *Paenibacillus* | 583979 |  |  |
| Firmicutes | *Paenibacillus* | 592043 |  |  |
| Firmicutes | *Sporacetigenium* | New.CleanUp.ReferenceOTU16354 |  |  |
| Firmicutes | *Sporacetigenium* | 4336569 |  |  |
| Firmicutes | *Anaerosolibacter* | 170026 |  |  |
| Firmicutes | *Clostridium* | New.CleanUp.ReferenceOTU18018 |  |  |
| Firmicutes | *Clostridium* | New.CleanUp.ReferenceOTU21937 |  |  |
| Firmicutes | *Clostridium* | 70947 |  |  |
| Firmicutes | *Clostridium* | 580518 |  |  |
| Firmicutes | *Clostridium* | 587789 |  |  |
| Firmicutes | *Clostridium* | 591223 |  |  |
| Firmicutes | *Clostridium* | 4483035 |  |  |
| Firmicutes | In the Clostridiaceae (*Clostridium, Fervidicella*) | 4322535 |  |  |
| Firmicutes | In the Clostridiaceae (*Anaerosolibacter, Thermotalea*) | New.CleanUp.ReferenceOTU15058 |  |  |
| Firmicutes | In the Clostridiaceae (*Anaerosolibacter, Thermotalea*) | 918577 |  |  |
| Gemmatimonadetes | In the Longimicrobiaceae clade (*Longimicrobium terrae)* | New.CleanUp.ReferenceOTU9456 | Aerobic chemoorganoheterotrophs/Slowly growing bacteria able to grow under low nutrient concentrations | [36] |
| Gemmatimonadetes | In the Longimicrobiaceae clade (*Longimicrobium terrae)* | New.CleanUp.ReferenceOTU371 |  |  |
| Gemmatimonadetes | In the Longimicrobiaceae clade (*Longimicrobium terrae)* | New.CleanUp.ReferenceOTU16764 |  |  |
| Gemmatimonadetes | In the Longimicrobiaceae clade (*Longimicrobium terrae)* | New.CleanUp.ReferenceOTU6912 |  |  |
| Gemmatimonadetes | In the Longimicrobiaceae clade (*Longimicrobium terrae)* | New.CleanUp.ReferenceOTU5046 |  |  |
| Gemmatimonadetes | In the Longimicrobiaceae clade (*Longimicrobium terrae)* | 1111500 |  |  |
| Gemmatimonadetes | In the Longimicrobiaceae clade (*Longimicrobium terrae)* | 804877 |  |  |
| Gemmatimonadetes | In the Longimicrobiaceae clade (*Longimicrobium terrae)* | 1108030 |  |  |
| Gemmatimonadetes | In the Longimicrobiaceae clade (*Longimicrobium terrae)* | New.CleanUp.ReferenceOTU3097 |  |  |
| Gemmatimonadetes | In the Longimicrobiaceae clade (*Longimicrobium terrae)* | 254895 |  |  |
| Gemmatimonadetes | In the Longimicrobiaceae clade (*Longimicrobium terrae)* | New.CleanUp.ReferenceOTU23585 |  |  |
| Gemmatimonadetes | In the Longimicrobiaceae clade (*Longimicrobium terrae)* | New.CleanUp.ReferenceOTU24127 |  |  |
| Gemmatimonadetes | In the Longimicrobiaceae clade (*Longimicrobium terrae)* | 4335435 |  |  |
| Gemmatimonadetes | In the Longimicrobiaceae clade (*Longimicrobium terrae)* | New.CleanUp.ReferenceOTU2153 |  |  |
| Gemmatimonadetes | In the Longimicrobiaceae clade (*Longimicrobium terrae)* | New.ReferenceOTU101 |  |  |
| Gemmatimonadetes | In the Longimicrobiaceae clade (*Longimicrobium terrae)* | New.CleanUp.ReferenceOTU11011 |  |  |
| Gemmatimonadetes | In the Longimicrobiaceae clade (*Longimicrobium terrae)* | 3312248 |  |  |
| Gemmatimonadetes | In the Longimicrobiaceae clade (*Longimicrobium terrae)* | 993930 |  |  |
| Gemmatimonadetes | In the Longimicrobiaceae clade (*Longimicrobium terrae)* | New.CleanUp.ReferenceOTU14916 |  |  |
| Gemmatimonadetes | In the Longimicrobiaceae clade (*Longimicrobium terrae)* | 257737 |  |  |
| Gemmatimonadetes | In the Longimicrobiaceae clade (*Longimicrobium terrae)* | 1007278 |  |  |
| Gemmatimonadetes | In the Longimicrobiaceae clade (*Longimicrobium terrae)* | 4461505 |  |  |
| Gemmatimonadetes | In the Longimicrobiaceae clade (*Longimicrobium terrae)* | 1075351 |  |  |
| Gemmatimonadetes | In the Longimicrobiaceae clade (*Longimicrobium terrae)* | New.CleanUp.ReferenceOTU8742 |  |  |
| Gemmatimonadetes | In the Gemmatimonadaceae clade (*Gemmatimonas phototrophica/G. Aurantica)* | 4427616 | Found in a variety of arid soils/Due to their biogeography and seasonal quantification in soils, an adaptation to low soil moisture has been suggested | [37] |
| Gemmatimonadetes | In the Gemmatimonadaceae clade (*Gemmatimonas phototrophica/G. Aurantica)* | 2738701 |  |  |
| Gemmatimonadetes | In the Gemmatimonadaceae clade (*Gemmatimonas phototrophica/G. Aurantica)* | New.CleanUp.ReferenceOTU23863 |  |  |
| Gemmatimonadetes | In the Gemmatimonadaceae clade (*Gemmatimonas phototrophica/G. Aurantica)* | 1104970 |  |  |
| Gemmatimonadetes | In the Gemmatimonadaceae clade (*Gemmatimonas phototrophica/G. Aurantica)* | 806026 |  |  |
| Gemmatimonadetes | In the Gemmatimonadaceae clade (*Gemmatimonas phototrophica/G. Aurantica)* | 4393102 |  |  |
| Gemmatimonadetes | In the Gemmatimonadaceae clade (*Gemmatimonas phototrophica/G. Aurantica)* | New.CleanUp.ReferenceOTU6151 |  |  |
| Gemmatimonadetes | In the Gemmatimonadaceae clade (*Gemmatimonas phototrophica/G. Aurantica)* | New.CleanUp.ReferenceOTU11566 |  |  |
| Gemmatimonadetes | In the Gemmatimonadaceae clade (*Gemmatimonas phototrophica/G. Aurantica)* | New.CleanUp.ReferenceOTU9835 |  |  |
| Gemmatimonadetes | In the Gemmatimonadaceae clade (*Gemmatimonas phototrophica/G. Aurantica)* | 4112169 |  |  |
| Gemmatimonadetes | In the Gemmatimonadaceae clade (*Gemmatimonas phototrophica/G. Aurantica)* | New.CleanUp.ReferenceOTU14650 |  |  |
| Gemmatimonadetes | In the Gemmatimonadaceae clade (*Gemmatimonas phototrophica/G. Aurantica)* | New.CleanUp.ReferenceOTU3345 |  |  |
| Gemmatimonadetes | In the Gemmatimonadaceae clade (*Gemmatimonas phototrophica/G. Aurantica)* | 909173 |  |  |
| Gemmatimonadetes | In the Gemmatimonadaceae clade (*Gemmatimonas phototrophica/G. Aurantica)* | New.CleanUp.ReferenceOTU16974 |  |  |
| Gemmatimonadetes | In the Gemmatimonadaceae clade (*Gemmatimonas phototrophica/G. Aurantica)* | 1103604 |  |  |
| Gemmatimonadetes | In the Gemmatimonadaceae clade (*Gemmatimonas phototrophica/G. Aurantica)* | New.CleanUp.ReferenceOTU8226 |  |  |
| Gemmatimonadetes | In the Gemmatimonadaceae clade (*Gemmatimonas phototrophica/G. Aurantica)* | New.CleanUp.ReferenceOTU18198 |  |  |
| Gemmatimonadetes | In the Gemmatimonadaceae clade (*Gemmatimonas phototrophica/G. Aurantica)* | 379634 |  |  |
| Gemmatimonadetes | In the Gemmatimonadaceae clade (*Gemmatimonas phototrophica/G. Aurantica)* | New.CleanUp.ReferenceOTU19383 |  |  |
| Gemmatimonadetes | In the Gemmatimonadaceae clade (*Gemmatimonas phototrophica/G. Aurantica)* | New.ReferenceOTU102 |  |  |
| Gemmatimonadetes | In the Gemmatimonadaceae clade (*Gemmatimonas phototrophica/G. Aurantica)* | 512952 |  |  |
| Gemmatimonadetes | In the Gemmatimonadaceae clade (*Gemmatimonas phototrophica/G. Aurantica)* | New.CleanUp.ReferenceOTU15842 |  |  |
| Gemmatimonadetes | In the Gemmatimonadaceae clade (*Gemmatimonas phototrophica/G. Aurantica)* | 959195 |  |  |
| Gemmatimonadetes | In the Gemmatimonadaceae clade (*Gemmatimonas phototrophica/G. Aurantica)* | 557467 |  |  |
| Gemmatimonadetes | In the Gemmatimonadaceae clade (*Gemmatimonas phototrophica/G. Aurantica)* | 1044938 |  |  |
| Gemmatimonadetes | In the Gemmatimonadaceae clade (*Gemmatimonas phototrophica/G. Aurantica)* | New.CleanUp.ReferenceOTU23369 |  |  |
| Gemmatimonadetes | In the Gemmatimonadaceae clade (*Gemmatimonas phototrophica/G. Aurantica)* | 855996 |  |  |
| Gemmatimonadetes | Unclassified | New.CleanUp.ReferenceOTU19441 |  |  |
| Nitrospirae | *Nitrospira* | 264343 | Chemolithoautotrophic nitrite-oxidizing bacteria/ *Nitrospira*-like bacteria take up inorganic carbon (HCO_3_^-^ and CO_2_) as well as pyruvate under aerobic conditions. | [38] |
| Planctomycetes | In the Phycisphaeraceae clade (*Algisphaera*/*Phycisphaera*) | New.CleanUp.ReferenceOTU17353 | Strictly aerobes, heterotrophs/Found in freshwater ponds and lakes, marine habitats, soils, wetlands | [39] |
| Planctomycetes | In the Phycisphaeraceae clade (*Algisphaera*/*Phycisphaera*) | New.CleanUp.ReferenceOTU977 |  |  |
| Planctomycetes | In the Phycisphaeraceae clade (*Algisphaera*/*Phycisphaera*) | 2649117 |  |  |
| Planctomycetes | In the Phycisphaeraceae clade (*Algisphaera*/*Phycisphaera*) | New.CleanUp.ReferenceOTU3936 |  |  |
| Planctomycetes | In the Phycisphaeraceae clade (*Algisphaera*/*Phycisphaera*) | 857776 |  |  |
| Planctomycetes | In the Phycisphaeraceae clade (*Algisphaera*/*Phycisphaera*) | New.CleanUp.ReferenceOTU11270 |  |  |
| Planctomycetes | In the Phycisphaeraceae clade (*Algisphaera*/*Phycisphaera*) | New.CleanUp.ReferenceOTU17520 |  |  |
| Planctomycetes | In the Phycisphaeraceae clade (*Algisphaera*/*Phycisphaera*) | 223655 |  |  |
| Planctomycetes | In the Phycisphaeraceae clade (*Algisphaera*/*Phycisphaera*) | 862250 |  |  |
| Planctomycetes | In the Phycisphaeraceae clade (*Algisphaera*/*Phycisphaera*) | New.CleanUp.ReferenceOTU907 |  |  |
| Planctomycetes | In the Phycisphaeraceae clade (*Algisphaera*/*Phycisphaera*) | New.CleanUp.ReferenceOTU20730 |  |  |
| Planctomycetes | In the Phycisphaeraceae clade (*Algisphaera*/*Phycisphaera*) | New.ReferenceOTU78 |  |  |
| Planctomycetes | In the Phycisphaeraceae clade (*Algisphaera*/*Phycisphaera*) | 4128584 |  |  |
| Planctomycetes | In the *Aquisphaera* clade | New.CleanUp.ReferenceOTU3721 |  |  |
| Planctomycetes | In the *Aquisphaera* clade | 801268 |  |  |
| Planctomycetes | *Pirellula staleyi* | 900959 |  |  |
| Proteobacteria | In the Alphaproteobacteria | New.CleanUp.ReferenceOTU15423 |  |  |
| Proteobacteria | In the Alphaproteobacteria | 151914 |  |  |
| Proteobacteria | In the Alphaproteobacteria | 806201 |  |  |
| Proteobacteria | In the Alphaproteobacteria | 332714 |  |  |
| Proteobacteria | In the Alphaproteobacteria | 4360812 |  |  |
| Proteobacteria | In the Alphaproteobacteria | New.CleanUp.ReferenceOTU16760 |  |  |
| Proteobacteria | In the Alphaproteobacteria | 985216 |  |  |
| Proteobacteria | In the Alphaproteobacteria | New.CleanUp.ReferenceOTU8617 |  |  |
| Proteobacteria | In the Alphaproteobacteria | 1109385 |  |  |
| Proteobacteria | In the Alphaproteobacteria | 3077248 |  |  |
| Proteobacteria | In the Alphaproteobacteria | New.CleanUp.ReferenceOTU20718 |  |  |
| Proteobacteria | In the Alphaproteobacteria | 813522 |  |  |
| Proteobacteria | In the Alphaproteobacteria | 308836 |  |  |
| Proteobacteria | In the Alphaproteobacteria | 223020 |  |  |
| Proteobacteria | In the Alphaproteobacteria | New.CleanUp.ReferenceOTU23837 |  |  |
| Proteobacteria | In the Alphaproteobacteria | New.ReferenceOTU90 |  |  |
| Proteobacteria | Geminicoccus | New.CleanUp.ReferenceOTU18861 | Aerobes/heterotrophic phototroph/Marine environments | [40] |
| Proteobacteria | Geminicoccus | 253754 |  |  |
| Proteobacteria | Geminicoccus | 154063 |  |  |
| Proteobacteria | Asticcacaulis | 1089812 | Aerobes or facultative anaerobes/Adapted to oligotrophic habitats | [41] |
| Proteobacteria | In the Caulobacteraceae | 817706 |  |  |
| Proteobacteria | In the Caulobacteraceae | 171288 |  |  |
| Proteobacteria | In the Caulobacteraceae | New.CleanUp.ReferenceOTU18760 |  |  |
| Proteobacteria | In the Caulobacteraceae | 560770 |  |  |
| Proteobacteria | In the Caulobacteraceae | New.CleanUp.ReferenceOTU13556 |  |  |
| Proteobacteria | *Microvirga* | New.CleanUp.ReferenceOTU16834 | Strictly aerobes/Reduces nitrate to nitrite/Found in soils, hot springs, and N_2_-fixing nodules of *Listia* and *Lupinus* | [42,43] |
| Proteobacteria | In the Rhizobiales (*Microvirga, Bosea*) | New.CleanUp.ReferenceOTU22677 | *Microvirga*: Strictly aerobes/Reduce nitrate to nitrite/Found in soils, hot springs, and N_2_-fixing nodules of *Listia* and *Lupinus*. *Bosea*: Isolated from agricultural soils, also found as a commensal inhabitants of legume nodules | [43,44] |
| Proteobacteria | In the Rhizobiales (*Microvirga, Bosea*) | New.CleanUp.ReferenceOTU22810 |  |  |
| Proteobacteria | In the Rhizobiales (*Microvirga, Bosea*) | New.CleanUp.ReferenceOTU7503 |  |  |
| Proteobacteria | In the Rhizobiales (*Microvirga, Bosea*) | 238412 |  |  |
| Proteobacteria | In the Rhizobiales (*Microvirga, Bosea*) | New.CleanUp.ReferenceOTU15783 |  |  |
| Proteobacteria | In the Rhizobiales (*Microvirga, Bosea*) | New.CleanUp.ReferenceOTU19694 |  |  |
| Proteobacteria | *Nitrobacter* | 107036 | *Nitrobacter f*ixes carbon dioxide via Calvin Cycle for their carbon requirements | [45] |
| Proteobacteria | In the Rhizobiales | 1090290 |  |  |
| Proteobacteria | In the Rhizobiales | 1977617 |  |  |
| Proteobacteria | In the Rhizobiales | 804156 |  |  |
| Proteobacteria | In the Rhizobiales | 137916 |  |  |
| Proteobacteria | In the Rhizobiales | 2984012 |  |  |
| Proteobacteria | In the Rhizobiales | 274150 |  |  |
| Proteobacteria | In the Rhizobiales | 1111551 |  |  |
| Proteobacteria | In the Rhizobiales | New.CleanUp.ReferenceOTU17090 |  |  |
| Proteobacteria | In the Rhizobiales | New.CleanUp.ReferenceOTU3195 |  |  |
| Proteobacteria | In the Rhizobiales | New.CleanUp.ReferenceOTU1010 |  |  |
| Proteobacteria | In the Rhizobiales | 247879 |  |  |
| Proteobacteria | In the Rhizobiales | 4371349 |  |  |
| Proteobacteria | In the Rhizobiales | 226516 |  |  |
| Proteobacteria | In the Rhizobiales | 764838 |  |  |
| Proteobacteria | In the Rhizobiales | 434250 |  |  |
| Proteobacteria | In the Rhizobiales | 2545365 |  |  |
| Proteobacteria | In the Rhizobiales | 362293 |  |  |
| Proteobacteria | In the Rhizobiales | 567776 |  |  |
| Proteobacteria | In the Rhizobiales | 681987 |  |  |
| Proteobacteria | In the Rhizobiales | New.ReferenceOTU98 |  |  |
| Proteobacteria | In the Rhizobiales | New.ReferenceOTU82 |  |  |
| Proteobacteria | In the Rhizobiales | New.CleanUp.ReferenceOTU6332 |  |  |
| Proteobacteria | In the Rhizobiales | 827636 |  |  |
| Proteobacteria | In the Rhizobiales | New.CleanUp.ReferenceOTU22633 |  |  |
| Proteobacteria | In the Rhizobiales | New.CleanUp.ReferenceOTU1020 |  |  |
| Proteobacteria | In the Rhizobiales | 589975 |  |  |
| Proteobacteria | In the Rhizobiales | 835594 |  |  |
| Proteobacteria | In the Rhizobiales | New.ReferenceOTU15 |  |  |
| Proteobacteria | In the Rhizobiales | 831289 |  |  |
| Proteobacteria | In the Rhizobiales | New.CleanUp.ReferenceOTU5261 |  |  |
| Proteobacteria | In the Rhizobiales | 142261 |  |  |
| Proteobacteria | *Rubellimicrobium* | New.ReferenceOTU25 | Strict aerobes, chemoorganotrophs/Found non-agricultural soils, air samples | [46] |
| Proteobacteria | *Rubellimicrobium* | 952388 |  |  |
| Proteobacteria | In the Rhodobacteraceae (*Rubellimicrobium*) | New.CleanUp.ReferenceOTU2092 |  |  |
| Proteobacteria | In the Rhodobacteraceae (*Rubellimicrobium*) | New.CleanUp.ReferenceOTU18421 |  |  |
| Proteobacteria | In the Rhodobacteraceae (Rubellimicrobium) | New.CleanUp.ReferenceOTU22269 |  |  |
| Proteobacteria | In the Rhodobacteraceae (Rubellimicrobium) | New.CleanUp.ReferenceOTU910 |  |  |
| Proteobacteria | In the Rhodobacteraceae (Rubellimicrobium) | New.CleanUp.ReferenceOTU17869 |  |  |
| Proteobacteria | In the Rhodobacteraceae (Rubellimicrobium) | 165827 |  |  |
| Proteobacteria | In the Rhodobacteraceae (Rubellimicrobium) | 151172 |  |  |
| Proteobacteria | In the Rhodobacteraceae (Rubellimicrobium) | 4348101 |  |  |
| Proteobacteria | In the Rhodobacteraceae (Rubellimicrobium) | 688714 |  |  |
| Proteobacteria | In the Rhodobacteraceae (*Rhodobacter*) | 1109246 | Purple non-sulfur photosynthetic bacteria/Found in soils and freshwater environments | [46] |
| Proteobacteria | In the Rhodobacteraceae (*Amaricoccus, Oceanicella*) | New.CleanUp.ReferenceOTU23462 | Aerobic chemoheterotrophs/ Found in soils and freshwater environments | [46] |
| Proteobacteria | In the Rhodobacteraceae (*Amaricoccus, Oceanicella*) | New.CleanUp.ReferenceOTU9107 |  |  |
| Proteobacteria | In the Rhodobacteraceae (*Amaricoccus, Oceanicella*) | New.CleanUp.ReferenceOTU12259 |  |  |
| Proteobacteria | In the Rhodospirillales (*Azospirillum, Skermanella*) | 241204 |  |  |
| Proteobacteria | In the Rhodospirillales (*Azospirillum, Skermanella*) | 828320 |  |  |
| Proteobacteria | In the Rhodospirillales (*Azospirillum, Skermanella*) | 906820 |  |  |
| Proteobacteria | In the Rhodospirillales (*Azospirillum, Skermanella*) | 4322410 |  |  |
| Proteobacteria | In the Rhodospirillales (*Azospirillum, Skermanella*) | 622731 |  |  |
| Proteobacteria | In the Rhodospirillales | 194558 |  |  |
| Proteobacteria | In the Rhodospirillales | 246217 |  |  |
| Proteobacteria | In the Rhodospirillales | New.CleanUp.ReferenceOTU6809 |  |  |
| Proteobacteria | In the Rhodospirillales | New.CleanUp.ReferenceOTU302 |  |  |
| Proteobacteria | In the Rhodospirillales | New.CleanUp.ReferenceOTU17584 |  |  |
| Proteobacteria | In the Rhodospirillales | 221365 |  |  |
| Proteobacteria | In the Rhodospirillales | 88754 |  |  |
| Proteobacteria | In the Rhodospirillales | New.CleanUp.ReferenceOTU24763 |  |  |
| Proteobacteria | In the Rhodospirillales | New.CleanUp.ReferenceOTU10460 |  |  |
| Proteobacteria | In the Rhodospirillales | 2693227 |  |  |
| Proteobacteria | In the Rhodospirillales | 882616 |  |  |
| Proteobacteria | In the Rhodospirillales | New.ReferenceOTU83 |  |  |
| Proteobacteria | In the Rhodospirillales | 370301 |  |  |
| Proteobacteria | In the Rhodospirillales | 348570 |  |  |
| Proteobacteria | In the Rhodospirillales | 701738 |  |  |
| Proteobacteria | In the Rhodospirillales | 197174 |  |  |
| Proteobacteria | In the Rhodospirillales | New.CleanUp.ReferenceOTU246 |  |  |
| Proteobacteria | In the Rhodospirillales | New.CleanUp.ReferenceOTU16878 |  |  |
| Proteobacteria | In the Rhodospirillales | 169755 |  |  |
| Proteobacteria | In the Rhodospirillales | 909097 |  |  |
| Proteobacteria | In the Rhodospirillales | New.CleanUp.ReferenceOTU19233 |  |  |
| Proteobacteria | In the Rhodospirillales | 1107148 |  |  |
| Proteobacteria | In the Rhodospirillales | 677964 |  |  |
| Proteobacteria | In the Rhodospirillales | 4562 |  |  |
| Proteobacteria | In the Rhodospirillales | New.CleanUp.ReferenceOTU21956 |  |  |
| Proteobacteria | In the Rhodospirillales | New.CleanUp.ReferenceOTU19807 |  |  |
| Proteobacteria | In the Sphingomonadaceae (*Sphingomonas*) | New.CleanUp.ReferenceOTU17671 | Chemoorganotrophs/ Its widespread distribution in the environment is due to its ability to utilize a wide range of organic compounds and to grow and survive under low nutrient conditions | [47] |
| Proteobacteria | In the Sphingomonadaceae (*Sphingomonas*) | New.ReferenceOTU6 |  |  |
| Proteobacteria | In the Sphingomonadaceae (*Sphingomonas*) | 674742 |  |  |
| Proteobacteria | In the Sphingomonadaceae (*Sphingomonas*) | 2324042 |  |  |
| Proteobacteria | In the Sphingomonadaceae (*Sphingomonas*) | 559317 |  |  |
| Proteobacteria | In the Sphingomonadaceae (*Sphingomonas*) | New.CleanUp.ReferenceOTU17989 |  |  |
| Proteobacteria | In the Sphingomonadaceae (*Sphingomonas*) | 329512 |  |  |
| Proteobacteria | In the Sphingomonadaceae (*Sphingomonas*) | New.CleanUp.ReferenceOTU17772 |  |  |
| Proteobacteria | In the Sphingomonadaceae (*Sphingomonas*) | 1003206 |  |  |
| Proteobacteria | In the Sphingomonadaceae (*Sphingomonas*) | New.CleanUp.ReferenceOTU12602 |  |  |
| Proteobacteria | In the Sphingomonadaceae (*Sphingomonas*) | New.CleanUp.ReferenceOTU3922 |  |  |
| Proteobacteria | In the Sphingomonadaceae (*Sphingomonas*) | 240087 |  |  |
| Proteobacteria | In the Sphingomonadaceae (*Sphingomonas*) | New.CleanUp.ReferenceOTU4783 |  |  |
| Proteobacteria | In the Sphingomonadaceae (*Sphingomonas*) | 699318 |  |  |
| Proteobacteria | In the Sphingomonadaceae (*Sphingomonas*) | 1052559 |  |  |
| Proteobacteria | In the Sphingomonadaceae (*Sphingomonas*) | 3723650 |  |  |
| Proteobacteria | In the Sphingomonadaceae (*Sphingomonas*) | 137881 |  |  |
| Proteobacteria | In the Sphingomonadaceae (*Sphingomonas*) | 113180 |  |  |
| Proteobacteria | In the Sphingomonadaceae (*Sphingomonas*) | 143392 |  |  |
| Proteobacteria | In the Sphingomonadaceae (*Sphingomonas*) | 343503 |  |  |
| Proteobacteria | In the Sphingomonadaceae (*Sphingomonas*) | 989109 |  |  |
| Proteobacteria | In the Sphingomonadaceae (*Sphingomonas*) | New.CleanUp.ReferenceOTU8851 |  |  |
| Proteobacteria | In the Sphingomonadaceae (*Sphingomonas*) | New.CleanUp.ReferenceOTU15767 |  |  |
| Proteobacteria | In the Sphingomonadaceae (*Sphingomonas*) | 222183 |  |  |
| Proteobacteria | In the Sphingomonadaceae (*Sphingomonas*) | 364155 |  |  |
| Proteobacteria | In the Sphingomonadaceae (*Sphingomonas*) | 494339 |  |  |
| Proteobacteria | In the Sphingomonadaceae (*Sphingomonas*) | 822489 |  |  |
| Proteobacteria | In the Sphingomonadaceae (*Sphingomonas*) | 810096 |  |  |
| Proteobacteria | In the Sphingomonadaceae (*Sphingomonas*) | 878663 |  |  |
| Proteobacteria | In the Sphingomonadaceae (*Sphingomonas*) | 552687 |  |  |
| Proteobacteria | In the Sphingomonadaceae (E*rythrobacter*) | 112754 |  |  |
| Proteobacteria | In the Betaproteobacteria | 4301666 |  |  |
| Proteobacteria | In the Betaproteobacteria | New.CleanUp.ReferenceOTU24192 |  |  |
| Proteobacteria | In the Betaproteobacteria | 567333 |  |  |
| Proteobacteria | In the Betaproteobacteria | 558494 |  |  |
| Proteobacteria | In the Betaproteobacteria | New.CleanUp.ReferenceOTU1622 |  |  |
| Proteobacteria | In the Oxalobacteaceae (*Herbaspirillum, Niviherbaspirillum*) | New.CleanUp.ReferenceOTU20068 | Aerobes, microaerobes to facultative anaerobes/ Found in soil, water and associated with plants | [48] |
| Proteobacteria | In the Oxalobacteaceae (*Herbaspirillum, Niviherbaspirillum*) | 111868 |  |  |
| Proteobacteria | In the Oxalobacteaceae (*Herbaspirillum, Niviherbaspirillum*) | 334185 |  |  |
| Proteobacteria | In the Oxalobacteaceae (*Herbaspirillum, Niviherbaspirillum*) | 553957 |  |  |
| Proteobacteria | In the Oxalobacteaceae (*Herbaspirillum, Niviherbaspirillum*) | 573270 |  |  |
| Proteobacteria | In the Oxalobacteaceae (*Herbaspirillum, Niviherbaspirillum*) | New.CleanUp.ReferenceOTU4490 |  |  |
| Proteobacteria | In the Oxalobacteaceae (*Herbaspirillum, Niviherbaspirillum*) | 533198 |  |  |
| Proteobacteria | In the Oxalobacteaceae (*Herbaspirillum, Niviherbaspirillum*) | 7346 |  |  |
| Proteobacteria | In the Oxalobacteaceae (*Herbaspirillum, Niviherbaspirillum*) | 759916 |  |  |
| Proteobacteria | In the Oxalobacteaceae (*Herbaspirillum, Niviherbaspirillum*) | 566578 |  |  |
| Proteobacteria | In the Oxalobacteaceae (*Herbaspirillum, Niviherbaspirillum*) | 256121 |  |  |
| Proteobacteria | In the Oxalobacteaceae (*Herbaspirillum, Niviherbaspirillum*) | 792868 |  |  |
| Proteobacteria | In the Oxalobacteaceae (*Herbaspirillum, Niviherbaspirillum*) | New.CleanUp.ReferenceOTU11316 |  |  |
| Proteobacteria | In the Oxalobacteaceae (*Herbaspirillum, Niviherbaspirillum*) | 586230 |  |  |
| Proteobacteria | In the Oxalobacteraceae (*Massilia*) | 1105574 |  |  |
| Proteobacteria | In the Oxalobacteraceae (*Massilia*) | 589123 |  |  |
| Proteobacteria | In the Oxalobacteraceae (*Massilia*) | New.ReferenceOTU60 |  |  |
| Proteobacteria | In the Oxalobacteraceae (*Massilia*) | 539915 |  |  |
| Proteobacteria | In the Oxalobacteraceae (*Massilia*) | New.CleanUp.ReferenceOTU1626 |  |  |
| Proteobacteria | In the Oxalobacteraceae (*Massilia*) | 849156 |  |  |
| Proteobacteria | In the Oxalobacteraceae (*Massilia*) | 941487 |  |  |
| Proteobacteria | In the Oxalobacteraceae (*Massilia*) | 210201 |  |  |
| Proteobacteria | In the Oxalobacteraceae (*Massilia*) | 822419 |  |  |
| Proteobacteria | In the Oxalobacteraceae (*Massilia, Janthinobacterium*) | 1033018 |  |  |
| Proteobacteria | In the Oxalobacteraceae (*Massilia, Janthinobacterium*) | 510182 |  |  |
| Proteobacteria | In the Comamonadaceae | New.CleanUp.ReferenceOTU5566 | Found in soil and water habitats | [49] |
| Proteobacteria | *Caenimonas* | 895220 | Chemoorganotrophs, strictly aerobes/Found in soils and sludge | [49] |
| Proteobacteria | *Piscinibacter* | 810167 | Chemoroganotrophs and facultative aerobes | [50] |
| Proteobacteria | In the Bdellovibrionales (*Bdellovibrio, Peredibacter, Bacteriovoraz*) | New.CleanUp.ReferenceOTU24305 | Gram-negative obligate predator of other gram-negative bacteria | [51] |
| Proteobacteria | In the Bdellovibrionales (*Bdellovibrio, Peredibacter, Bacteriovoraz*) | New.CleanUp.ReferenceOTU20697 |  |  |
| Proteobacteria | In the Bdellovibrionales (*Bdellovibrio, Peredibacter, Bacteriovoraz*) | 4455981 |  |  |
| Proteobacteria | In the Bdellovibrionales | New.CleanUp.ReferenceOTU22326 |  |  |
| Proteobacteria | In the Bdellovibrionales | New.CleanUp.ReferenceOTU14933 |  |  |
| Proteobacteria | In the Bdellovibrionales | New.CleanUp.ReferenceOTU12590 |  |  |
| Proteobacteria | In the Bdellovibrionales | 554390 |  |  |
| Proteobacteria | In the Bdellovibrionales | New.CleanUp.ReferenceOTU1597 |  |  |
| Proteobacteria | In the Bdellovibrionales | 185100 |  |  |
| Proteobacteria | In the Deltaproteobacteria | New.CleanUp.ReferenceOTU24860 |  |  |
| Proteobacteria | In the Deltaproteobacteria | New.CleanUp.ReferenceOTU19985 |  |  |
| Proteobacteria | In the Deltaproteobacteria | New.CleanUp.ReferenceOTU8091 |  |  |
| Proteobacteria | In the Deltaproteobacteria | New.CleanUp.ReferenceOTU882 |  |  |
| Proteobacteria | In the Deltaproteobacteria | New.CleanUp.ReferenceOTU10609 |  |  |
| Proteobacteria | In the Deltaproteobacteria | 541209 |  |  |
| Proteobacteria | In the Deltaproteobacteria | New.CleanUp.ReferenceOTU376 |  |  |
| Proteobacteria | In the Deltaproteobacteria | 817141 |  |  |
| Proteobacteria | In the Deltaproteobacteria | New.CleanUp.ReferenceOTU13553 |  |  |
| Proteobacteria | In the Deltaproteobacteria | New.CleanUp.ReferenceOTU14472 |  |  |
| Proteobacteria | In the Deltaproteobacteria | New.CleanUp.ReferenceOTU18196 |  |  |
| Proteobacteria | In the Deltaproteobacteria | 958375 |  |  |
| Proteobacteria | *Oligoflexus* | 852722 | Aerobes, non-motile and non-spore forming | [52] |
| Proteobacteria | Sister clade to *Oligoflexus* | New.CleanUp.ReferenceOTU9482 |  |  |
| Proteobacteria | In the Oligoflexales | New.ReferenceOTU28 |  |  |
| Proteobacteria | In the Myxococcales | New.CleanUp.ReferenceOTU15168 |  |  |
| Proteobacteria | In the Myxococcales | New.CleanUp.ReferenceOTU8231 |  |  |
| Proteobacteria | In the Myxococcales | 841077 |  |  |
| Proteobacteria | In the Myxococcales | 1107143 |  |  |
| Proteobacteria | In the Myxococcales | 336745 |  |  |
| Proteobacteria | In the Myxococcales | New.CleanUp.ReferenceOTU18084 |  |  |
| Proteobacteria | In the Myxococcales | New.CleanUp.ReferenceOTU15594 |  |  |
| Proteobacteria | In the Myxococcales | New.CleanUp.ReferenceOTU19587 |  |  |
| Proteobacteria | In the Myxococcales | New.CleanUp.ReferenceOTU2758 |  |  |
| Proteobacteria | In the Myxococcales | 4353063 |  |  |
| Proteobacteria | In the Myxococcales | New.CleanUp.ReferenceOTU2732 |  |  |
| Proteobacteria | In the Myxococcales | 993373 |  |  |
| Proteobacteria | In the Myxococcales | 2963709 |  |  |
| Proteobacteria | In the Myxococcales | New.CleanUp.ReferenceOTU5897 |  |  |
| Proteobacteria | In the Myxococcales | 4366579 |  |  |
| Proteobacteria | In the Myxococcales | New.CleanUp.ReferenceOTU17390 |  |  |
| Proteobacteria | In the Myxococcales | New.CleanUp.ReferenceOTU21396 |  |  |
| Proteobacteria | In the Myxococcales | New.CleanUp.ReferenceOTU20960 |  |  |
| Proteobacteria | In the Myxococcales | 113261 |  |  |
| Proteobacteria | In the Myxococcales | New.CleanUp.ReferenceOTU23678 |  |  |
| Proteobacteria | In the Myxococcales | New.ReferenceOTU10 |  |  |
| Proteobacteria | In the Myxococcales | 1131498 |  |  |
| Proteobacteria | In the Myxococcales | 4432545 |  |  |
| Proteobacteria | In the Myxococcales | New.CleanUp.ReferenceOTU24375 |  |  |
| Proteobacteria | In the Myxococcales | 237206 |  |  |
| Proteobacteria | In the Myxococcales | New.CleanUp.ReferenceOTU16687 |  |  |
| Proteobacteria | In the Myxococcales | 254949 |  |  |
| Proteobacteria | In the Myxococcales | New.CleanUp.ReferenceOTU4810 |  |  |
| Proteobacteria | In the Myxococcales | 4302753 |  |  |
| Proteobacteria | In the Myxococcales | 501684 |  |  |
| Proteobacteria | In the Myxococcales | 1021984 |  |  |
| Proteobacteria | In the Myxococcales | 808319 |  |  |
| Proteobacteria | In the Myxococcales | New.CleanUp.ReferenceOTU8715 |  |  |
| Proteobacteria | In the Myxococcales | 240506 |  |  |
| Proteobacteria | In the Myxococcales | New.CleanUp.ReferenceOTU16645 |  |  |
| Proteobacteria | In the Myxococcales | 279206 |  |  |
| Proteobacteria | In the Myxococcales | 313833 |  |  |
| Proteobacteria | In the Myxococcales | New.CleanUp.ReferenceOTU23156 |  |  |
| Proteobacteria | In the Myxococcales | New.CleanUp.ReferenceOTU11165 |  |  |
| Proteobacteria | In the Myxococcales | 4299497 |  |  |
| Proteobacteria | In the Myxococcales | New.CleanUp.ReferenceOTU3193 |  |  |
| Proteobacteria | In the Myxococcales | 1023267 |  |  |
| Proteobacteria | In the Myxococcales | 1017063 |  |  |
| Proteobacteria | In the Myxococcales | 4461509 |  |  |
| Proteobacteria | In the Myxococcales | 2441354 |  |  |
| Proteobacteria | In the Myxococcales | 824043 |  |  |
| Proteobacteria | In the Myxococcales | New.CleanUp.ReferenceOTU16846 |  |  |
| Proteobacteria | In the Myxococcales | New.CleanUp.ReferenceOTU5142 |  |  |
| Proteobacteria | In the Myxococcales | New.CleanUp.ReferenceOTU1612 |  |  |
| Proteobacteria | In the Myxococcales | New.CleanUp.ReferenceOTU3192 |  |  |
| Proteobacteria | In the Myxococcales | 259044 |  |  |
| Proteobacteria | In the Myxococcales | 803166 |  |  |
| Proteobacteria | In the Myxococcales | New.CleanUp.ReferenceOTU8504 |  |  |
| Proteobacteria | In the Myxococcales | 559177 |  |  |
| Proteobacteria | In the Myxococcales | New.CleanUp.ReferenceOTU2855 |  |  |
| Proteobacteria | In the Myxococcales | New.CleanUp.ReferenceOTU1041 |  |  |
| Proteobacteria | In the Gammaproteobacteria | New.CleanUp.ReferenceOTU13139 |  |  |
| Proteobacteria | In the Gammaproteobacteria | 1118948 |  |  |
| Proteobacteria | Sister clade to *Haliea/Halioglobus* | 3038080 |  |  |
| Proteobacteria | Sister clade to *Haliea/Halioglobus* | 931708 |  |  |
| Proteobacteria | *Lysobacter* | 751138 |  |  |
| Proteobacteria | Unassigned | 253724 |  |  |
| Proteobacteria | Unassigned | 369436 |  |  |
| Proteobacteria | Unassigned | 865778 |  |  |
| Proteobacteria | Unassigned | 1524233 |  |  |
| Proteobacteria | Unassigned | 256515 |  |  |
| Proteobacteria | Unassigned | New.CleanUp.ReferenceOTU1861 |  |  |
| Proteobacteria | Unassigned | New.CleanUp.ReferenceOTU16225 |  |  |
| Proteobacteria | Unassigned | New.CleanUp.ReferenceOTU19976 |  |  |
| Proteobacteria | Unassigned | New.CleanUp.ReferenceOTU23963 |  |  |
| Proteobacteria | Unassigned | 830015 |  |  |
| Proteobacteria | Unassigned | New.CleanUp.ReferenceOTU12552 |  |  |
| Proteobacteria | Unassigned | New.CleanUp.ReferenceOTU481 |  |  |
| Verrucomicrobia | In the Chthoniobacterales clade | New.CleanUp.ReferenceOTU7500 | Aerobes, non-motile | [53] |
| Verrucomicrobia | In the Chthoniobacterales clade | New.CleanUp.ReferenceOTU14345 |  |  |
| Verrucomicrobia | In the Chthoniobacterales clade | New.CleanUp.ReferenceOTU20422 |  |  |
| Verrucomicrobia | In the Chthoniobacterales clade | New.CleanUp.ReferenceOTU4737 |  |  |
| Verrucomicrobia | In the Chthoniobacterales clade | 219498 |  |  |
| Verrucomicrobia | In the Chthoniobacterales clade | 142335 |  |  |
| Verrucomicrobia | In the Chthoniobacterales clade | New.CleanUp.ReferenceOTU7883 |  |  |
| Verrucomicrobia | In the Chthoniobacterales clade | New.CleanUp.ReferenceOTU4765 |  |  |
| Verrucomicrobia | In the Chthoniobacterales clade | 624312 |  |  |
| Verrucomicrobia | In the Chthoniobacterales clade | 538238 |  |  |
| Verrucomicrobia | In the Chthoniobacterales clade | New.CleanUp.ReferenceOTU21867 |  |  |
| Verrucomicrobia | In the Chthoniobacterales clade | 553562 |  |  |
| Verrucomicrobia | In the Chthoniobacterales clade | New.CleanUp.ReferenceOTU17962 |  |  |
| Verrucomicrobia | In the Chthoniobacterales clade | 922698 |  |  |
| Verrucomicrobia | In the Chthoniobacterales clade | New.ReferenceOTU13 |  |  |
| Verrucomicrobia | In the Chthoniobacterales clade | New.CleanUp.ReferenceOTU543 |  |  |
| Verrucomicrobia | In the Chthoniobacterales clade | 251499 |  |  |
| Verrucomicrobia | In the Chthoniobacterales clade | 1049393 |  |  |
| Verrucomicrobia | In the Chthoniobacterales clade | New.CleanUp.ReferenceOTU9264 |  |  |
| Verrucomicrobia | In the Chthoniobacterales clade | 544067 |  |  |
| Verrucomicrobia | In the Chthoniobacterales clade | 352632 |  |  |
| Verrucomicrobia | In the Chthoniobacterales clade | 564262 |  |  |
| Verrucomicrobia | In the Chthoniobacterales clade | New.ReferenceOTU16 |  |  |
| Verrucomicrobia | In the Chthoniobacterales clade | 547960 |  |  |
| Verrucomicrobia | In the Chthoniobacterales clade | 1108624 |  |  |
| Verrucomicrobia | In the Chthoniobacterales clade | 559200 |  |  |
| Verrucomicrobia | In the Chthoniobacterales clade | New.CleanUp.ReferenceOTU8439 |  |  |
| Verrucomicrobia | In the Chthoniobacterales clade | New.CleanUp.ReferenceOTU8199 |  |  |
| Verrucomicrobia | In the Chthoniobacterales clade | 1078065 |  |  |
| Verrucomicrobia | In the Chthoniobacterales clade | New.CleanUp.ReferenceOTU10655 |  |  |
| Verrucomicrobia | In the Chthoniobacterales clade | 1028297 |  |  |
| Verrucomicrobia | In the Chthoniobacterales clade | New.CleanUp.ReferenceOTU14191 |  |  |
| Verrucomicrobia | In the Chthoniobacterales clade | 4480292 |  |  |
| Verrucomicrobia | In the Chthoniobacterales clade | 971170 |  |  |
| Verrucomicrobia | In the Chthoniobacterales clade | 807473 |  |  |
| Verrucomicrobia | In the Chthoniobacterales clade | New.ReferenceOTU57 |  |  |
| Verrucomicrobia | In the Chthoniobacterales clade | 586320 |  |  |
| Verrucomicrobia | *Opitutus* | 142010 | Obligate anaerobes/Found in soil | [54] |
| Verrucomicrobia | In the Verrucomicrobiaceae clade | 3426090 |  |  |
| Verrucomicrobia | In the Verrucomicrobiaceae clade (*Luteolibacter*) | 540464 |  |  |
| Unassigned | Unassigned | New.CleanUp.ReferenceOTU13671 |  |  |
| Unassigned | Unassigned | NewReferenceOTU97 |  |  |
| Unassigned | Unassigned | 542433 |  |  |
| Unassigned | Unassigned | 509980 |  |  |
| Unassigned | Unassigned | 586275 |  |  |
| Unassigned | Unassigned | 1128021 |  |  |
| Unassigned | Unassigned | 274632 |  |  |
| Unassigned | Unassigned | 509899 |  |  |
| Unassigned | Unassigned | 2834426 |  |  |
| Unassigned | Unassigned | New.CleanUp.ReferenceOTU20553 |  |  |
| Unassigned | Unassigned | New.CleanUp.ReferenceOTU22663 |  |  |
| Unassigned | Unassigned | New.CleanUp.ReferenceOTU7002 |  |  |
| Unassigned | Unassigned | New.CleanUp.ReferenceOTU9775 |  |  |
| Unassigned | Unassigned | New.CleanUp.ReferenceOTU10725 |  |  |
| Unassigned | Unassigned | New.CleanUp.ReferenceOTU17710 |  |  |
| Unassigned | Unassigned | New.CleanUp.ReferenceOTU24680 |  |  |
| Unassigned | Unassigned | New.CleanUp.ReferenceOTU8452 |  |  |
| Unassigned | Unassigned | New.CleanUp.ReferenceOTU14422 |  |  |
| Unassigned | Unassigned | 205635 |  |  |
| Unassigned | Unassigned | 205900 |  |  |
| Unassigned | Unassigned | 356083 |  |  |
| Unassigned | Unassigned | 587047 |  |  |
| Unassigned | Unassigned | 819659 |  |  |
| Unassigned | Unassigned | New.CleanUp.ReferenceOTU13513 |  |  |
| Unassigned | Unassigned | New.CleanUp.ReferenceOTU22946 |  |  |
| Unassigned | Unassigned | New.CleanUp.ReferenceOTU2691 |  |  |
| Unassigned | Unassigned | New.CleanUp.ReferenceOTU7950 |  |  |
| Unassigned | Unassigned | New.CleanUp.ReferenceOTU9079 |  |  |
| Unassigned | Unassigned | New.CleanUp.ReferenceOTU16975 |  |  |
| Unassigned | Unassigned | 344495 |  |  |
| Unassigned | Unassigned | 4311457 |  |  |
| Unassigned | Unassigned | New.CleanUp.ReferenceOTU23333 |  |  |
| Unassigned | Unassigned | 547148 |  |  |
| Unassigned | Unassigned | New.CleanUp.ReferenceOTU7999 |  |  |
| Unassigned | Unassigned | New.CleanUp.ReferenceOTU5586 |  |  |

**References**

1. Pascual J, Wüst PK, Geppert A, Foesel BU, Huber KJ. Novel isolates double the number of chemotrophic species and allow the first description of higher taxa in Acidobacteria subdivision 4. 2015;38:534–44.

2. Huber KJ, Geppert AM, Wanner G. The first representative of the globally widespread subdivision 6 Acidobacteria , Vicinamibacter silvestris gen . nov ., sp . nov ., isolated from subtropical savannah soil. 2016;2971–9.

3. Fukunaga Y, Ichikawa N. The Class Holophagaceae. In: Rosenberg E, DeLong EF, Lory S, Stackebrandt E, Thompson F, editors. Prokaryotes Other Major Lineages Bact Archaea. 4th ed. London: Springer-Verlag Berlin Heidelberg; 2014. p. 683–8. Available from: http://www.springerlink.com/index/10.1007/0-387-30742-7

4. Rosenberg E. The family Deinococcaceae. In: Rosenberg E, DeLong EF, Lory S, Stackebrandt E, Thompson F, editors. Prokaryotes Other Major Lineages Bact Archaea. 4th ed. London: Springer-Verlag Berlin Heidelberg; 2014. p. 613–6.

5. Stackebrandt E. The Familily Acidimicrobiaceae. In: Rosenberg E, DeLong EF, Lory S, Stackebrandt E, Thompson F, editors. The Prokaryotes: Actinobacteria. 4th ed. London: Springer-Verlag Berlin Heidelberg; 2014. p. 5–12.

6. Tamura T, Ishida Y, Otoguro M, Yamamura H, Hayakawa M, Suzuki KI. Angustibacter luteus gen. nov., sp. nov., isolated from subarctic forest soil. Int J Syst Evol Microbiol. 2010;60:2441–5.

7. Labeda D, Testa R. Saccharothrix: a new genus of the Actinomycetales related to Nocardiopsis. Int J Syst Bacteriol. 1984;34:426–31. Available from: http://ijs.sgmjournals.org/content/34/4/426.short

8. Stackebrandt E, Schumann P. The Family Cellulomonadaceae. In: Rosenberg E, DeLong EF, Lory S, Stackebrandt E, Thompson F, editors. The Prokaryotes: Actinobacteria. 4th ed. London: Springer-Verlag Berlin Heidelberg; 2014. p. 163–80.

9. Franco MMC, Labeda DP. The Order Pseudonocardiales. In: Rosenberg E, DeLong EF, Lory S, Stackebrandt E, Thompson F, editors. The Prokaryotes: Actinobacteria. 4th ed. London: Springer-Verlag Berlin Heidelberg; 2014. p. 745–850.

10. Normand P, Daffonchio D, Gtari M. The familiy Geodermatophilaceae. In: Rosenberg E, DeLong EF, Lory S, Stackebrandt E, Thompson F, editors. The Prokaryotes: Actinobacteria. 4th ed. London: Springer-Verlag Berlin Heidelberg; 2014. p. 361–77.

11. Stackebrandt E, Scheuner C, Goker M, Schumann P. The Family Intraspoangiaceae. In: Rosenberg E, DeLong EF, Lory S, Stackebrandt E, Thompson F, editors. The Prokaryotes: Actinobacteria. 4th ed. London: Springer-Verlag Berlin Heidelberg; 2014. p. 397–420.

12. Tamura T, Suzuki K-I. The Suborder Kineosporiineae. In: Rosenberg E, DeLong EF, Lory S, Stackebrandt E, Thompson F, editors. The Prokaryotes: Actinobacteria. 4th ed. London: Springer-Verlag Berlin Heidelberg; 2014. p. 443–52.

13. Busse H-J, Wieser M. The Genus Arthrobacter. In: Rosenberg E, DeLong EF, Lory S, Stackebrandt E, Thompson F, editors. The Prokaryotes: Actinobacteria. 4th ed. London: Springer-Verlag Berlin Heidelberg; 2014. p. 105–26.

14. Trujillo ME, Hong K, Genilloud O. The Family Micromonosporaceae. In: Rosenberg E, DeLong EF, Lory S, Stackebrandt E, Thompson F, editors. The Prokaryotes: Actinobacteria. 4th ed. London: Springer-Verlag Berlin Heidelberg; 2014. p. 500–61.

15. Tohn EM, Borsodi A. The Family Nocardioidaceae. In: Rosenberg E, DeLong EF, Lory S, Stackebrandt E, Thompson F, editors. The Prokaryotes: Actinobacteria. 4th ed. London: Springer-Verlag Berlin Heidelberg; 2014. p. 651–90.

16. Kim KK, Lee J-S. The Family Nakamurellaceae. In: Rosenberg E, DeLong EF, Lory S, Stackebrandt E, Thompson F, editors. The Prokaryotes: Actinobacteria. 4th ed. London: Springer-Verlag Berlin Heidelberg; 2014. p. 577–84.

17. Stackebrandt E, Otten LG. The Class Nitriliruptoria. In: Rosenberg E, DeLong EF, Lory S, Stackebrandt E, Thompson F, editors. The Prokaryotes: Actinobacteria. 4th ed. London: Springer-Verlag Berlin Heidelberg; 2014. p. 587–93.

18. Alburquerque L, da Costa MS. The Families Conexibacteraceae, Patulibacteraceae and Solirubrobacteraceae. In: Rosenberg E, DeLong EF, Lory S, Stackebrandt E, Thompson F, editors. The Prokaryotes: Actinobacteria. 4th ed. London: Springer-Verlag Berlin Heidelberg; 2014. p. 185–99.

19. Alburquerque L, da Costa MS. The Family Rubrobacteraceae. In: Rosenberg E, DeLong EF, Lory S, Stackebrandt E, Thompson F, editors. The Prokaryotes: Actinobacteria. 4th ed. London: Springer-Verlag Berlin Heidelberg; 2014. p. 861–5.

20. Alburquerque L, da Costa MS. The Family Gaiellaceae. In: Rosenberg E, DeLong EF, Lory S, Stackebrandt E, Thompson F, editors. The Prokaryotes: Actinobacteria. 4th ed. London: Springer-Verlag Berlin Heidelberg; 2014. p. 357–60.

21. Im W-T, Hu Z-Y, Kim K-H, Rhee S-K, Meng H, Lee S-T, et al. Description of Fimbriimonas ginsengisoli gen. nov., sp. nov. within the Fimbriimonadia class nov., of the phylum Armatimonadetes. Antonie Van Leeuwenhoek. 2012;102:307–17. Available from: https://doi.org/10.1007/s10482-012-9739-6

22. Lee KCY, Dunfield PF, B. SM. The Phylum Armatimonadetes. In: Rosenberg E, DeLong EF, Lory S, Stackebrandt E, Thompson F, editors. Prokaryotes Other Major Lineages Bact Archaea. 4th ed. London: Springer-Verlag Berlin Heidelberg; 2014. p. 447–57.

23. Kang JY, Chun J, Seo JW, Kim CH, Jahng KY. Flaviaesturariibacter amylovorans gen. nov., sp. nov., a starch-hydrolysing bacterium, isolated from estuarine water. Int J Syst Evol Microbiol. 2015;65:2209–14.

24. Rosenberg E. The Family Chitinophagaceae. In: Rosenberg E, DeLong EF, Lory S, Stackebrandt E, Thompson F, editors. Prokaryotes Other Major Lineages Bact Archaea. 4th ed. London: Springer-Verlag Berlin Heidelberg; 2014. p. 493–5.

25. Rashidan KK, Bird DF. Role of predatory bacteria in the termination of a cyanobacterial bloom. Microb Ecol . 2001;41:97–105. Available from: https://doi.org/10.1007/s002480000074

26. Mcbride MJ, Liu W, Xuemei L, Zhu Y, Zhang W, Lu X, et al. The Family Cytophagaceae. In: Rosenberg E, DeLong EF, Lory S, Stackebrandt E, Thompson F, editors. Prokaryotes Other Major Lineages Bact Archaea. 4th ed. London: Springer-Verlag Berlin Heidelberg; 2014. p. 577–93. Available from: http://link.springer.com/10.1007/978-3-642-38954-2

27. Yoon J, Adachi K, Park S, Kasai H, Yokota A. Aureibacter tunicatorum gen. nov., sp. nov., a marine bacterium isolated from a coral reef sea squirt, and description of flammeovirgaceae fam. nov. Int J Syst Evol Microbiol. 2011;61:2342–7.

28. McBride MJ. The Family Flavobacteriaceae. In: Rosenberg E, DeLong EF, Lory S, Stackebrandt E, Thompson F, editors. Prokaryotes Other Major Lineages Bact Archaea. 4th ed. London: Springer-Verlag Berlin Heidelberg; 2014. p. 643–77.

29. Lambiase A. The Family Sphingobacteriaceae. In: Rosenberg E, DeLong EF, Lory S, Stackebrandt E, Thompson F, editors. Prokaryotes Other Major Lineages Bact Archaea. 4th ed. London: Springer-Verlag Berlin Heidelberg; 2014. p. 907–12.

30. Iino T. The Family Ignavibacteriaceae. In: Rosenberg E, DeLong EF, Lory S, Stackebrandt E, Thompson F, editors. Prokaryotes Other Major Lineages Bact Archaea. 4th ed. London: Springer-Verlag Berlin Heidelberg; 2014. p. 701–2.

31. Hanada S. The Phylum Chloroflexi, the Family Chloroflexaceae, and the Related Phototrophic Families Oscillochloridaceae and Roseiflexaceae. In: Rosenberg E, DeLong EF, Lory S, Stackebrandt E, Thompson F, editors. Prokaryotes Other Major Lineages Bact Archaea. Fourth. London: Springer-Verlag Berlin Heidelberg; 2014. p. 515–29.

32. Stieglmeier M, Alves RJE, Schleper C. The Phylum Thaumarchaeota. In: Rosenberg E, DeLong EF, Lory S, Stackebrandt E, Thompson F, editors. Prokaryotes Other Major Lineages Bact Archaea. 4th ed. London: Springer-Verlag Berlin Heidelberg; 2014. p. 347–58.

33. Raven JA. Carbon. In: Whitton BA, editor. Ecol Cyanobacteria II. London: Springer-Verlag Berlin Heidelberg; 2012. p. 443–60.

34. Komárek J, Kaštovský J, Mareš J, Johansen JR. Taxonomic classification of cyanoprokaryotes (cyanobacterial genera) 2014, using a polyphasic approach. Preslia. 2014;86:295–335.

35. Ludwing W, Schleifer K-H, Whitman WB. Volume 3: Firmicutes. In: Vos P, Garrity G, Jones D, Krieg NR, Ludwing W, F.A. R, et al., editors. Bergey’s Man Syst Bacteriol Vol 3 Firmicutes. New York: Springer; 2009. p. 1–13.

36. Pascual J, García-López M, Bills GF, Genilloud O. Longimicrobium terrae gen. Nov., sp. Nov., an oligotrophic bacterium of the under-represented phylum gemmatimonadetes isolated through a system of miniaturized diffusion chambers. Int J Syst Evol Microbiol. 2016;66:1976–85.

37. DeBruyn JM, Nixon LT, Fawaz MN, Johnson AM, Radosevich M. Global biogeography and quantitative seasonal dynamics of Gemmatimonadetes in soil. Appl Environ Microbiol. 2011;77:6295–300.

38. Daims H, Nielsen JL, Nielsen PHERH, Wagner M, Schleifer K, Nielsen PHERH, et al. In Situ Characterization of Nitrospira -Like Nitrite-Oxidizing Bacteria Active in Wastewater Treatment Plants. Appl Environ Microbiol. 2001;67:5273–84.

39. Youssef NH, Elshahed MS. The Phylum Planctomycetes. In: Rosenberg E, DeLong EF, Lory S, Stackebrandt E, Thompson F, editors. Prokaryotes Other Major Lineages Bact Archaea. 4th ed. London: Springer-Verlag Berlin Heidelberg; 2014. p. 759–804.

40. Foesel BU, Gößner AS, Drake HL, Schramm A. Geminicoccus roseus gen. nov., sp. nov., an aerobic phototrophic Alphaproteobacterium isolated from a marine aquaculture biofilter. Syst Appl Microbiol. 2007;30:581–6.

41. Abraham W-R, Rohde M, Bennasar A. The Family Caulobacteraceae. In: Rosenberg E, DeLong EF, Lory S, Stackebrandt E, Thompson F, editors. The Prokaryotes: Alphaproteobacteria and Betaproteobacteria. 4th ed. London: Springer-Verlag Berlin Heidelberg; 2012. p. 179–200.

42. Bailey AC, Kellom M, Poret-Peterson AT, Noonan K, Hartnett HE, Raymond J. Draft Genome Sequence of Microvirga sp. Strain BSC265, Isolated from Biological Soil Crust of Moab, Utah. Genome Announc. 2014;2:e01199-14-e01199-14.

43. Kelly DP, McDonald IR, Wood AP. The Family Methylobacteriaceae. In: Rosenberg E, DeLong EF, Lory S, Stackebrandt E, Thompson F, editors. The Prokaryotes: Alphaproteobacteria and Betaproteobacteria. 4th ed. Londond: Springer-Verlag Berlin Heidelberg; 2014. p. 313–33.

44. Marin I, Ruiz Arahal D. The Family Beijerinckiaceae. In: Rosenberg E, DeLong EF, Lory S, Stackebrandt E, Thompson F, editors. The Prokaryotes: Alphaproteobacteria and Betaproteobacteria. 4th ed. London: Springer-Verlag Berlin Heidelberg; 2014. p. 114–29.

45. Marcondes de Souza JA, Carareto Alves LM, de Mello Varani A, de Macedo Lemos EG. the Family Bradyrhizobiaceae. In: Rosenberg E, DeLong EF, Lory S, Stackebrandt E, Thompson F, editors. The Prokaryotes: Alphaproteobacteria and Betaproteobacteria. 4th ed. London: Springer-Verlag Berlin Heidelberg; 2014. p. 135–50.

46. Pujalte M, Lucena T, Ruvira M, Ruiz Arahal D, Macian MC. The Family Rhodobacteraceae. In: Rosenberg E, DeLong EF, Lory S, Stackebrandt E, Thompson F, editors. The Prokaryotes: Alphaproteobacteria and Betaproteobacteria. 4th ed. London: Springer-Verlag Berlin Heidelberg; 2014. p. 439–512.

47. Glaeser S, Kampfer P. The Family Sphingomonadaceae. In: Rosenberg E, DeLong EF, Lory S, Stackebrandt E, Thompson F, editors. The Prokaryotes: Alphaproteobacteria and Betaproteobacteria. 4th ed. London: Springer-Verlag Berlin Heidelberg; 2014. p. 642–93.

48. Baldani JI, Rouws L, Magalhaes Cruz L, Lopes Olivares F. The Family Oxalobacteraceae. In: Rosenberg E, DeLong EF, Lory S, Stackebrandt E, Thompson F, editors. The Prokaryotes: Alphaproteobacteria and Betaproteobacteria. 4th ed. London: Springer-Verlag Berlin Heidelberg; 2014. p. 920–68.

49. Willems A. The Family Comamonadaceae. In: Rosenberg E, DeLong EF, Lory S, Stackebrandt E, Thompson F, editors. The Prokaryotes: Alphaproteobacteria and Betaproteobacteria. 4th ed. London: Springer-Verlag Berlin Heidelberg; 2014. p. 777–845.

50. Stackebrandt E, Verbarg S, Frühling A, Busse HJ, Tindall BJ. Dissection of the genus Methylibium: Reclassification of Methylibium fulvum as Rhizobacter fulvus comb. nov., Methylibium aquaticum as Piscinibacter aquaticus gen. nov., comb. nov. and Methylibium subsaxonicum as Rivibacter subsaxonicus gen. nov., comb. n. Int J Syst Evol Microbiol. 2009;59:2552–60.

51. Rotem O, Pasternak Z, Jurkevitch E. Bdellovibrio and Like Organisms. In: Rosenberg E, DeLong EF, Lory S, Stackebrandt E, Thompson F, editors. The Prokaryotes: Deltaproteobacteria and Epsilonproteobacteria. 4th ed. London: Springer-Verlag Berlin Heidelberg; 2014. p. 3–13.

52. Nakai R, Nishijima M, Tazato N, Handa Y, Karray F, Sayadi S, et al. Oligoflexus tunisiensis gen. nov., sp. nov., a Gram-negative, aerobic, filamentous bacterium of a novel proteobacterial lineage, and description of Oligoflexaceae fam. nov., Oligoflexales ord. nov. and Oligoflexia classis nov. Int J Syst Evol Microbiol. 2014;64:3353–9. Available from: http://www.pubmedcentral.nih.gov/articlerender.fcgi?artid=4179278&tool=pmcentrez&rendertype=abstract

53. Sangwan P, Chen X, Hugenholtz P, Janssen PH. Chthoniobacter flavus gen. nov., sp. nov., the first pure-culture representative of subdivision two, Spartobacteria classis nov., of the phylum Verrucomicrobia. Appl Environ Microbiol. 2004;70:5875–81.

54. Chin K, Liesack W, Janssen PH. Opitutus terrae gen. nov., sp. nov., to accommodate novel strains of the division ‘ Verrucomicrobia ’ isolated from rice paddy soil. Int J Syst Evol Microbiol. 2001;51:1965–8.
