# Supplementary material for: Spatial segregation of the biological soil crust microbiome around its foundational cyanobacterium, Microcoleus vaginatus, and the formation of a nitrogen-fixing cyanosphere
Source: Microbiome. 2019 Apr 3;7:55. doi: 10.1186/s40168-019-0661-2 (PMC6448292; doi:10.1186/s40168-019-0661-2)
Supplement: Supplementary file 5 — Table S5. Potential contaminants. Responding OTUs detected after amplification and sequencing of negative controls (n = 5) without target Microcoleus bundles. (DOCX 17 kb) [file 40168_2019_661_MOESM4_ESM.docx]

**Supplementary Table 5. Potential contaminants.**  Responding OTUs detected after amplification and sequencing of negative controls (n=5) without target *Microcoleus* bundles.

| **Phylum** | **Deepest Taxonomic Assignment** | **OTU ID** | **Presence in controls** |
| --- | --- | --- | --- |
| Firmicutes | *Staphylococcus* | 1084865 | 1 out of 5 |
| Betaproteobacteria | *Pelomonas saccharophila* | 1108275 | 3 out of 5 |
| Gammaproteobacteria | *Moraxella* | 990864 | 2 out of 5 |
| Gammaproteobacteria | *Escherichia/Shigella* | 1111294 | 2 out of 5 |
