# Supplementary material for: Spatial segregation of the biological soil crust microbiome around its foundational cyanobacterium, Microcoleus vaginatus, and the formation of a nitrogen-fixing cyanosphere
Source: Microbiome. 2019 Apr 3;7:55. doi: 10.1186/s40168-019-0661-2 (PMC6448292; doi:10.1186/s40168-019-0661-2)
Supplement: Supplementary file 6 — Table S6. NMDS plot coordinates. The corresponding plot is displayed in Fig. 3a (DOCX 18 kb) [file 40168_2019_661_MOESM6_ESM.docx]

**Table S6: NMDS plot coordinates, stress = 0.19**

| **Samples ID** | **Sampling Location** | **Sample type** | **NMDS1** | **NMDS2** |
| --- | --- | --- | --- | --- |
| FBsample26 | Chihuahuan - hot desert - FB | Bundle | 0.541067955 | 0.03270142 |
| FBsample2 | Chihuahuan - hot desert - FB | Bundle | -0.095036701 | 0.756269337 |
| FBsample04 | Chihuahuan - hot desert - FB | Bundle | 0.089537598 | 0.550864033 |
| FBsample06 | Chihuahuan - hot desert - FB | Bundle | 1.03828475 | 1.041164708 |
| FBsample22 | Chihuahuan - hot desert - FB | Bundle | 1.279165465 | 0.782020429 |
| FBsample28 | Chihuahuan - hot desert - FB | Bundle | -0.093173843 | 0.434975513 |
| FBsample30 | Chihuahuan - hot desert - FB | Bundle | 0.368640495 | 0.699881219 |
| FBsample33 | Chihuahuan - hot desert - FB | Bundle | 0.39116946 | 0.240863986 |
| FBsample14 | Chihuahuan - hot desert - FB | Bundle | 0.857078838 | 0.313375232 |
| FBsample17 | Chihuahuan - hot desert - FB | Bundle | 0.350617238 | 0.325977733 |
| FBsample24 | Chihuahuan - hot desert - FB | Bundle | 0.078014451 | 0.201483853 |
| FBsample3 | Chihuahuan - hot desert - FB | Bundle | 0.010570804 | 0.947836735 |
| FBsample01 | Chihuahuan - hot desert - FB | Bundle | 1.004377068 | 0.428131656 |
| FBsample4 | Chihuahuan - hot desert - FB | Bundle | 0.628859432 | 1.217023785 |
| FBsample15 | Chihuahuan - hot desert - FB | Bundle | 0.223300481 | 0.500795325 |
| FBsample25 | Chihuahuan - hot desert - FB | Bundle | 0.757776145 | 0.721729906 |
| FBsample29 | Chihuahuan - hot desert - FB | Bundle | 0.555508803 | 0.580861082 |
| FBsample35 | Chihuahuan - hot desert - FB | Bundle | 0.132647339 | 0.329376862 |
| FBsample09 | Chihuahuan - hot desert - FB | Bundle | 0.036816431 | 0.76823846 |
| FBsample5 | Chihuahuan - hot desert - FB | Bundle | 0.340596489 | 0.951804148 |
| FBsample19 | Chihuahuan - hot desert - FB | Bundle | 1.444075236 | -0.272307778 |
| FBsample08 | Chihuahuan - hot desert - FB | Bundle | 0.887249898 | -0.055735098 |
| FBsample07 | Chihuahuan - hot desert - FB | Bundle | 1.19805035 | -0.032623922 |
| FBsample11 | Chihuahuan - hot desert - FB | Bundle | 1.602799759 | -0.444358491 |
| FBsampleA | Chihuahuan - hot desert - FB | Soil | -1.454068723 | 0.943452081 |
| FBsampleC | Chihuahuan - hot desert - FB | Soil | -1.446388466 | 0.920811909 |
| FBsampleB | Chihuahuan - hot desert - FB | Soil | -1.355312391 | 0.891972516 |
| HSNsample34 | Great Basin - cold desert -HSN | Bundle | -0.442206851 | -0.380843598 |
| HSNsample43 | Great Basin - cold desert -HSN | Bundle | -0.918000381 | -0.596413104 |
| HSNsample27 | Great Basin - cold desert -HSN | Bundle | -0.028137068 | -0.625175139 |
| HSNsample01 | Great Basin - cold desert -HSN | Bundle | -0.398857528 | -0.515091999 |
| HSNsample11 | Great Basin - cold desert -HSN | Bundle | -0.886308924 | -0.112102963 |
| HSNsample37 | Great Basin - cold desert -HSN | Bundle | -0.623156242 | -0.426091468 |
| HSNsample42 | Great Basin - cold desert -HSN | Bundle | -0.758333496 | -0.34888961 |
| HSNsample23 | Great Basin - cold desert -HSN | Bundle | -0.320728124 | -0.318598607 |
| HSNsample25 | Great Basin - cold desert -HSN | Bundle | -0.008081234 | -1.59577054 |
| HSNsample46 | Great Basin - cold desert -HSN | Bundle | 0.296683137 | -1.064262085 |
| HSNsample44 | Great Basin - cold desert -HSN | Bundle | -0.374461854 | -0.778164577 |
| HSNsample29 | Great Basin - cold desert -HSN | Bundle | 0.43294007 | -0.67671792 |
| HSNsample45 | Great Basin - cold desert -HSN | Bundle | -0.262530325 | -1.097825993 |
| HSNsample22 | Great Basin - cold desert -HSN | Bundle | -0.222568743 | -0.822046931 |
| HSNsample15 | Great Basin - cold desert -HSN | Bundle | 0.000163404 | -0.478522493 |
| HSNsample18 | Great Basin - cold desert -HSN | Bundle | -0.53667243 | -0.248053222 |
| HSNsample28 | Great Basin - cold desert -HSN | Bundle | -0.711532025 | -0.747696882 |
| HSNsample24 | Great Basin - cold desert -HSN | Bundle | 0.545069157 | -0.708929132 |
| HSNsample10 | Great Basin - cold desert -HSN | Bundle | 0.561490446 | -0.991746836 |
| HSNsample20 | Great Basin - cold desert -HSN | Bundle | 0.776936944 | -1.571495814 |
| HSNsampleB | Great Basin - cold desert -HSN | Soil | -1.829414637 | 0.09839824 |
| HSNsampleC | Great Basin - cold desert -HSN | Soil | -1.796675536 | 0.102452126 |
| HSNsampleA | Great Basin - cold desert -HSN | Soil | -1.867842122 | 0.127001906 |
